# Nanotechnology in healthcare, and its safety and environmental risks

**DOI:** 10.1186/s12951-024-02901-x

**Published:** 2024-11-15

**Authors:** Xiaohan Ma, Yaxin Tian, Ren Yang, Haowei Wang, Latifa W. Allahou, Jinke Chang, Gareth Williams, Jonathan C. Knowles, Alessandro Poma

**Affiliations:** 1 Division of Biomaterials and Tissue Engineering, Eastman Dental Institute, Royal Free Hospital, University College London, Rowland Hill Street, London, NW3 2PF UK; 2 United InnoMed (Shanghai) Limited, F/2, E-1, No.299, Kangwei Rd, Pudong District, Shanghai, China; 3 Centre for Precision Healthcare, UCL Division of Medicine, University College London, London, WC1E 6JF UK; 4 UCL School of Pharmacy, University College London, 29-39 Brunswick Square, London, WC1N 1AX UK; 5 UCL Centre for Biomaterials in Surgical Reconstruction and Regeneration, Division of Surgery & Interventional Science, University College London, London, NW3 2PF UK; 6 Department of Nanobiomedical Science and BK21 PLUS NBM Global Research Center for Regenerative Med-Icine, Dankook University, Cheonan, 31116 South Korea; 7 UCL Eastman-Korea Dental Medicine Innovation Centre, Dankook University, Cheonan, 31116 South Korea

**Keywords:** Nanotechnology, Healthcare, Safety, Environmental risks, Regulatory policies

## Abstract

Nanotechnology holds immense promise in revolutionising healthcare, offering unprecedented opportunities in diagnostics, drug delivery, cancer therapy, and combating infectious diseases. This review explores the multifaceted landscape of nanotechnology in healthcare while addressing the critical aspects of safety and environmental risks associated with its widespread application. Beginning with an introduction to the integration of nanotechnology in healthcare, we first delved into its categorisation and various materials employed, setting the stage for a comprehensive understanding of its potential. We then proceeded to elucidate the diverse healthcare applications of nanotechnology, spanning medical diagnostics, tissue engineering, targeted drug delivery, gene delivery, cancer therapy, and the development of antimicrobial agents. The discussion extended to the current situation surrounding the clinical translation and commercialisation of these cutting-edge technologies, focusing on the nanotechnology-based healthcare products that have been approved globally to date. We also discussed the safety considerations of nanomaterials, both in terms of human health and environmental impact. We presented the in vivo health risks associated with nanomaterial exposure, in relation with transport mechanisms, oxidative stress, and physical interactions. Moreover, we highlighted the environmental risks, acknowledging the potential implications on ecosystems and biodiversity. Lastly, we strived to offer insights into the current regulatory landscape governing nanotechnology in healthcare across different regions globally. By synthesising these diverse perspectives, we underscore the imperative of balancing innovation with safety and environmental stewardship, while charting a path forward for the responsible integration of nanotechnology in healthcare.

**Graphical abstract:**

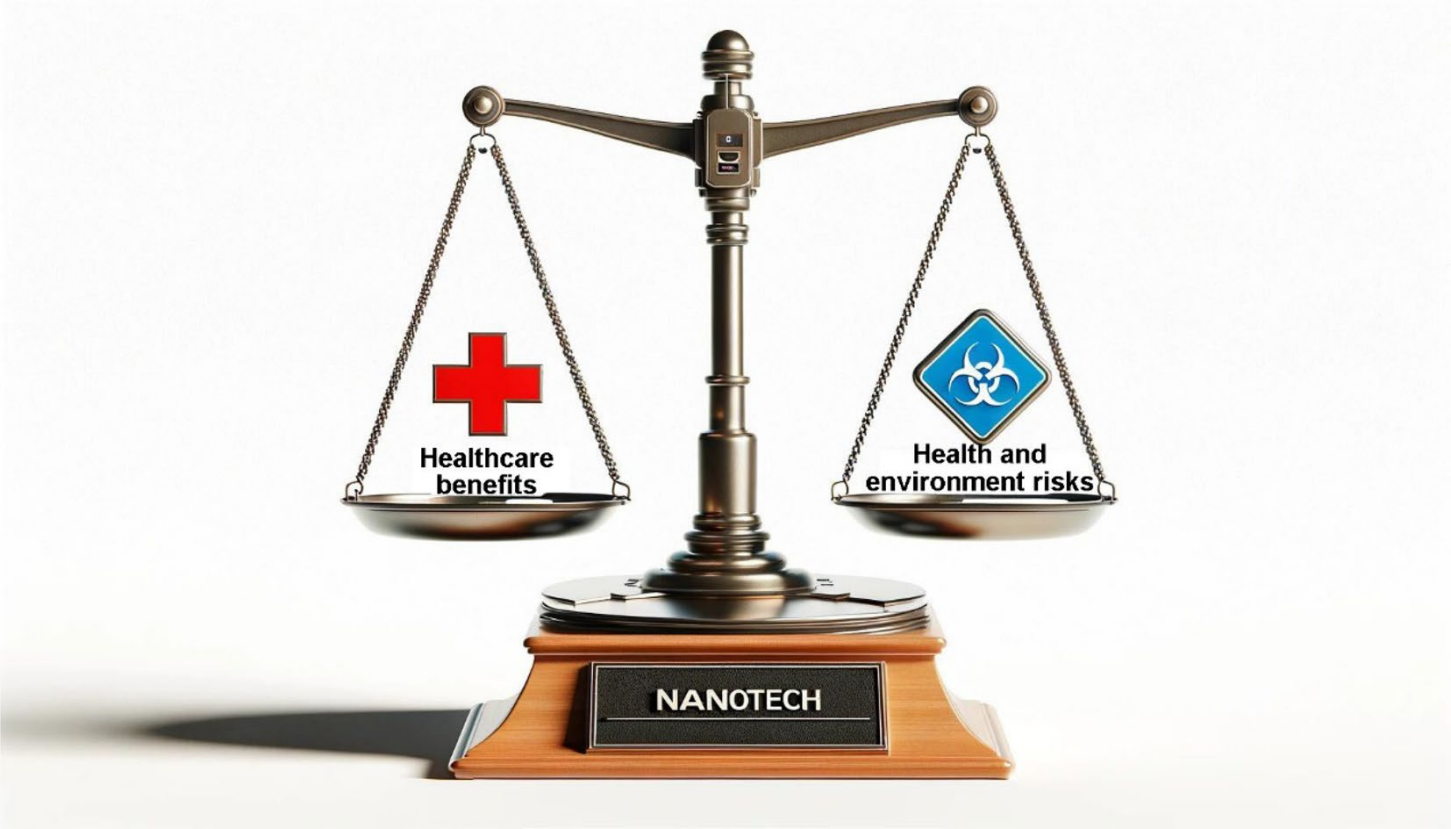

## Introduction

Nanomaterials are defined as materials that possess one or more peripheral nanoscale dimensions (in the range 1–100 nm). Nanotechnology, the field dedicated to the science and applications of these nanomaterials, is experiencing rapid and continuous growth. At this scale, the properties of materials undergo significant alterations. Characteristics such as solubility, reactivity, spectroscopy, electrical and magnetic attributes, as well as membrane transport, typically diverge from those exhibited by the same materials at larger scales [1]. The unique properties exhibited by nanomaterials open up avenues for diverse applications and hold great promise for transformative advancements in various scientific and technological domains.

In recent years, nanotechnology has emerged as a transformative force in the field of healthcare, offering continued innovation in medicine development and unprecedented possibilities for enhancing the performance and applications of medical devices. The ability to manipulate matter at the nanoscale has paved the way for groundbreaking innovations, promising revolutionary breakthroughs in diagnostics, treatment modalities, and overall patient care. Specific areas demonstrating the potential of nanotechnology in healthcare include medical diagnostics, where nanomaterials are used to improve the sensitivity and accuracy of imaging and biosensing techniques, enabling earlier and more precise detection of diseases [2, 3]. In tissue engineering, nanomaterials enhance cell interactions and tissue regeneration, offering promising advancements in areas like neural, dental, bone, and skin repair [4]. Furthermore, nanomaterials are being developed for drug and gene delivery systems, where their ability to target specific cells or tissues can significantly enhance the efficacy of treatments while minimising side effects [5]. In cancer therapies, nanomaterials can enhance the efficacy of traditional treatments such as surgery, radiotherapy, and chemotherapy, while also enabling the development of novel therapeutic approaches including biotherapy, photothermal therapy, and photodynamic therapy [6]. Additionally, nanomaterials are being employed in antimicrobial and antiviral applications, including the creation of coatings and materials that can prevent infections and combat resistant pathogens [7].

Despite the immense potential, however, the current application of nanotechnology in medicines and medical devices faces substantial challenges, both of a technical nature and within the complex realm of regulatory policies [8, 9]. This review aims to provide a comprehensive overview of the present status of nanotechnology in the medical domain, focusing on three critical dimensions: clinical applications, safety and environmental considerations, and the global regulatory landscape. By delving into the intricacies of these key aspects, we seek to elucidate the current achievements, gaps, and hurdles in the integration of nanotechnology into medicines and medical devices. Our exploration will shed light on the diverse facets of this dynamic field, addressing the promising clinical advancements, potential safety concerns, and the evolving regulatory frameworks that shape the landscape of nanotechnology in healthcare.

## Advanced nanotechnology in healthcare

### Categories and classification of nanotechnology materials

**Fig. 1:**
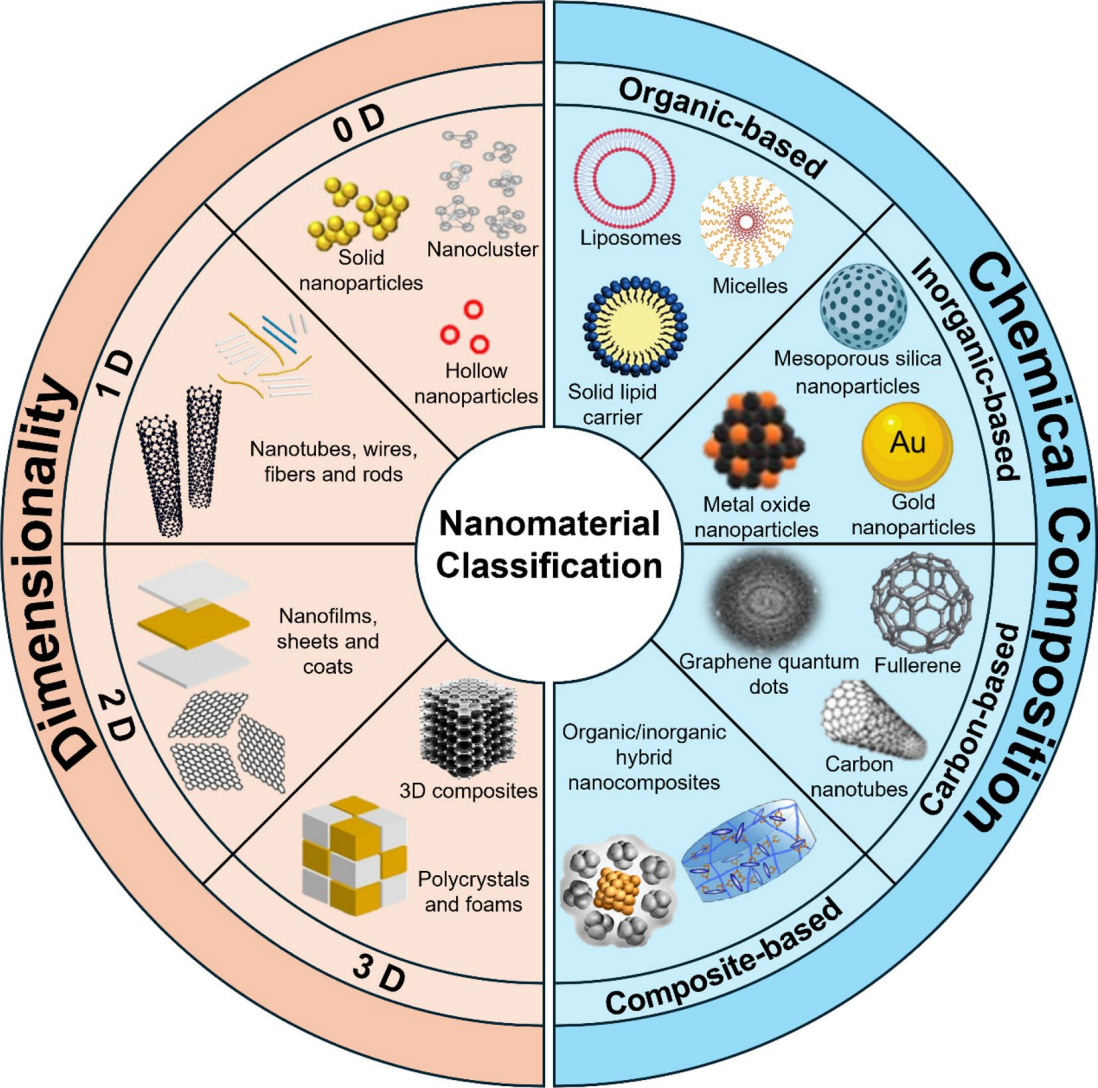
Classifications of nanomaterials based on dimensionality and chemical composition

Nanomaterials can be categorised into four primary types based on the degree of spatial confinement [10] (Fig. 1). These include (i) zero-dimensional nanomaterials, where all dimensions are on the nanometer scale (*e.g.*, nanoparticles), (ii) one-dimensional nanomaterials, where any one of the three dimensions is in the nanometer range (e.g., nanorods, nanowires, etc.), (iii) two-dimensional nanomaterials, where any two of the three dimensions are nanometer-sized (e.g., nanosheets, nanoplates, and nanocoatings), and (iv) three-dimensional nanomaterials, with each dimension in nanometer scale, thus allowing electrons to move freely without being confined in any direction. Examples include nanoflowers, nanocubes, nanocages, nanowire bundles, and various self-assemblies of lower-dimensional nanomaterials.

In addition to spatial confinement, nanomaterials can also be classified based on origin, porosity, phase and dispersion [11]. The multitude of classifications reflects the abundance of nanotechnology materials, which are playing an increasingly important role in healthcare applications. However, a more common method of classifying nanomaterials is based on their chemical composition [12]. According to this, they can be classified into four material-based categories: organic-based, inorganic-based, carbon-based and composite-based nanomaterials (Fig. 1).

#### Organic-based nanomaterials

**Fig. 2:**
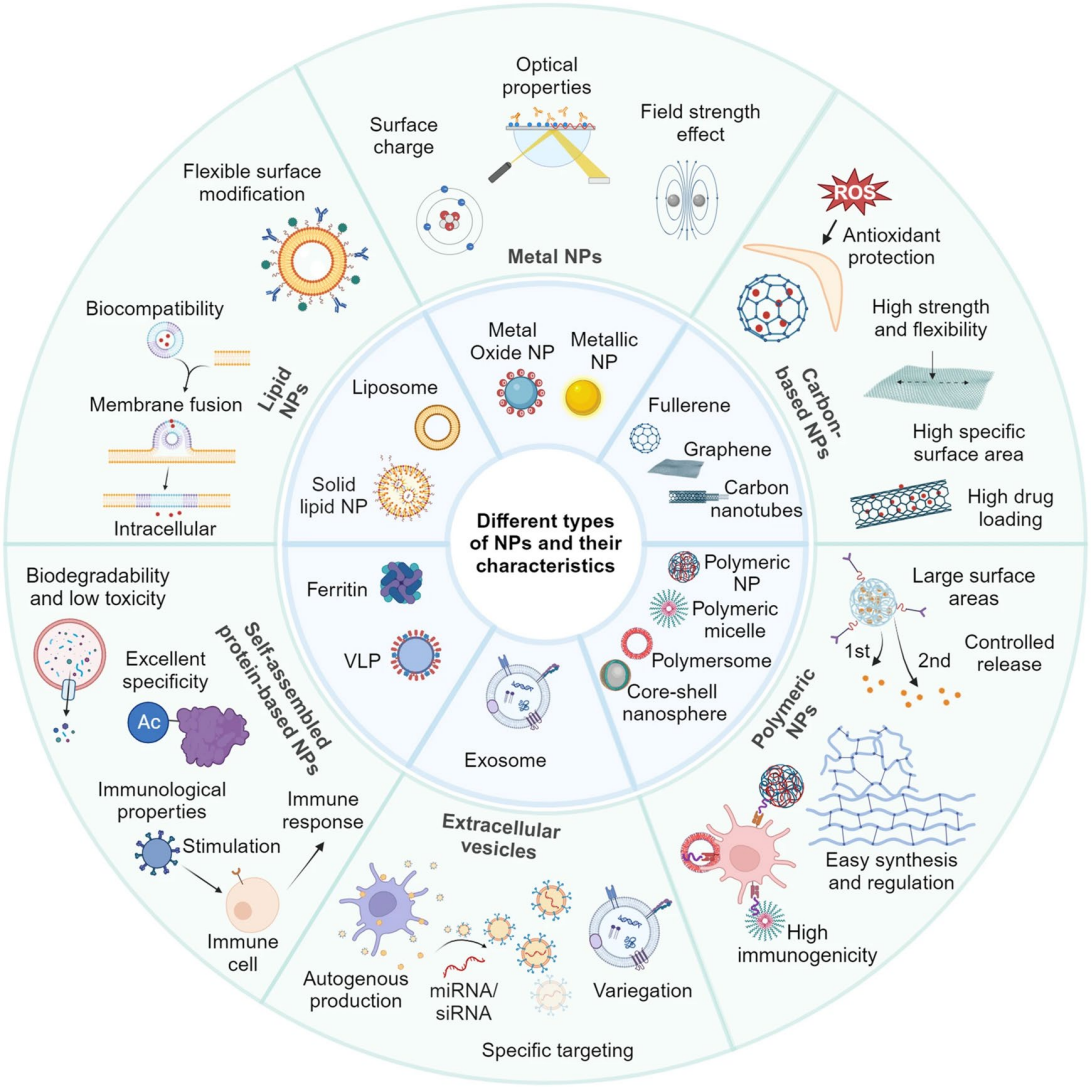
Characteristics of six common nanomaterials. Lipid NPs, composed of lipids like phospholipids, offer excellent biocompatibility and flexible surface modification capabilities. Metal NPs, including metals such as gold, silver, and copper, as well as their oxides, exhibit outstanding optical, electronic, and magnetic properties, making them ideal for biological imaging, photothermal therapy (PTT), and sensing applications. Carbon-based nanomaterials, such as CNTs, graphene, and fullerenes, feature a large surface area, high drug-loading capacity, and chemical stability, providing resistance to oxidative environments. Polymeric NPs, made from various polymers, display diverse structures and properties suitable for multiple biomedical applications. Self-assembled NPs, including ferritin family proteins and virus-like particles (VLPs), offer good biodegradability in the case of ferritin proteins and the ability to mimic viral stimuli to trigger immune responses in the case of VLPs. Exosomes, small vesicles secreted by cells, are rich in proteins, nucleic acids, and signaling molecules, playing vital roles in cellular communication and regulation. These nanomaterials have broad applications in drug delivery, molecular imaging, biosensing, tissue engineering, and disease diagnosis. Adapted with permission from Shen et al*.* [37]

Organic-based nanomaterials, which are formed via covalent or non-covalent assemblies of organic molecules, encompass a wide range of types used in healthcare applications, mainly including micelles, dendrimers, liposomes, nanogels, polymeric nanoparticles (NPs), extracellular vesicles, and nanoscale covalent-organic frameworks (COFs) [13, 14]. The majority of them, with the exception of a limited number of modernist molecular machines, are polymeric in nature [15]. These materials are highly regarded for their biocompatibility, making them ideal for in vivo applications, with certain types, such as aliphatic polyesters, being particularly renowned for their exceptional biodegradability [16]. Additionally, organic-based nanomaterials can be easily functionalised, allowing for precise control over their chemical compositions, shape, size, and surface properties, making them highly adaptable to a variety of biomedical applications, such as bioimaging, drug delivery, and therapy (Fig. 2) [17, 18]. For instance, semiconducting polymeric NPs are prized for their high extinction coefficients, photostability, and tunable emission profiles in the near-infrared (NIR) spectrum, making them ideal for deep-tissue imaging, including NIR-II fluorescence and photoacoustic imaging [19, 20]. Additionally, COFs have emerged as promising candidates in drug delivery and phototherapeutics due to their large porosity and superior photoelectric properties [21, 22]. Liposomes, vesicles, and micelles, which are extensively used for drug encapsulation, play a crucial role in minimising the off-target toxicity of potent therapeutic agents [23–26]. The core composition, dimensions, and surface characteristics of these organic NPs are critical factors that determine their biocompatibility and functional efficacy in vivo.

However, despite their advantages, organic-based nanomaterials also present certain limitations, including lower mechanical strength and stability compared to inorganic counterparts, which can restrict their use in applications requiring high structural integrity and thermal stability [27]. Additionally, batch-to-batch variability in synthesis can affect reproducibility and scalability [28]. Nonetheless, ongoing research aims to address these challenges, further enhancing the potential of organic nanomaterials in healthcare.

#### Inorganic-based nanomaterials

Inorganic-based nanomaterials not only exhibit better chemical and mechanical stability compared to their organic counterparts, but also possess unique physicochemical advantages, such as optical, electrical, magnetic, ultrasonic, and catalytic properties [29, 30]. These advantages make them highly promising for biomedical applications, particularly in cancer imaging and therapy. However, their potential toxicity and poor biocompatibility, which can trigger adverse immune responses, induce inflammation, immunogenicity, and long-term toxicity, complicating their clinical translation [31]. In addition, inorganic-based nanomaterials often suffer from limited biodegradability, leading to accumulation in tissues and organs, which can pose significant risks over extended periods [32]. Nonetheless, progress in design and surface modification is steadily enhancing the safety and clinical potential of inorganic-based nanomaterials.

Generally, inorganic-based nanomaterials in healthcare applications include different metal and metal oxide nanomaterials. Examples of metal-based inorganic nanomaterials are silver (Ag), gold (Au), aluminum (Al), cadmium (Cd), copper (Cu), iron (Fe), zinc (Zn), and lead (Pb) nanomaterials, whereas examples of metal oxide-based inorganic nanomaterials are zinc oxide (ZnO), copper oxide (CuO), magnesium aluminum oxide (MgAl_2_O_4_), TiO_2_, cerium oxide (CeO_2_), and iron oxide (Fe_3_O_4_), etc. Figure 2 highlights the advantages of metal and metal oxide NPs in healthcare applications. In addition, silicon-based nanomaterials, including porous silicon nanoparticles (pSiNPs), mesoporous silica nanoparticles (MSNs) and periodic mesoporous silica nanoparticles (PMONPs), have been intensively investigated for therapeutic and diagnostic applications [33]. Moreover, layered double hydroxides (LDHs), metal carbides and nitrides (MXenes), and 2D transition metal borides (MBenes) are emerging as promising inorganic-based two-dimensional nanomaterials due to their exceptional surface area, tunable chemistry, and potential for drug delivery, biosensing, and theranostics [34, 35].

#### Carbon-based nanomaterials

Carbon-based nanomaterials are considered as a separate class of nanomaterial with features such as diversity in their structures and facile functionalisation [36]. Due to the unique properties, carbon can form covalent bonds with other carbons in different hybridisation states such as sp, sp^2^, and sp^3^ in order to form a variety of structures of small molecules and longer chains [12]. Graphene (Gr), graphene oxide (GO), carbon nanotubes (CNTs), fullerenes (C_60_), carbon dots (CDs), graphene quantum dots (GQDs), carbon nanofibers (CFs), carbon onions, and carbon black are the different categories of carbon-based nanomaterials. These materials are highly valued in healthcare for their exceptional surface area, electrical conductivity, and mechanical strength. However, like inorganic nanomaterials, they face challenges in biocompatibility that need to be addressed. Their potential applications span drug delivery, bioimaging, tissue engineering, and diagnostic sensing.

#### Composite-based nanomaterials

Composite-based nanomaterials, also known as hybrid nanomaterials, are any combination of metal-based, metal oxide-based, carbon-based, and/or organic-based nanomaterials, which often have complicated structures. Although many biomaterials use organic and inorganic materials individually, the increased demand for highly functionalised biomaterials has necessitated the development of organic/inorganic composite materials that not only integrate the advantages of each component, but also provide synergistic properties to meet new demands [38–41]. For example, water non-dispersible inorganic NPs, such as gold nanoparticles (Au NPs), Fe_3_O_4_ NPs, semiconductor QDs, CDs, etc., can be rendered dispersible in water through surface modification with organic polymers like polyethylene glycol (PEG) or polydopamine (PDA) [42]. This modification enables precise control over their size, surface charge, and functionalisation, while maintaining desirable physical properties of the inorganic core such as magnetic, optical or catalytic activity. As a result, the presence of a organic polymer shell not only improves the dispersion of inorganic nanomaterials in biological fluids, but can also significantly enhance the biocompatibility of the inorganic core, acting as anchor sites for molecular linkages or protecting them from oxidation [43]. Appropriately designed shell thicknesses can improve the chemical and thermal stability of NPs, while also regulating and controlling the release of molecules from the core [44].

Such core–shell NPs have been widely investigated for cancer therapy, specific delivery of therapeutics, drug delivery monitoring, biosensors, bioimaging and antimicrobial activity. For example, a biosensor for detecting influenza A viral HA proteins (H1 and H5) was developed by modifying the surface of a field effect transistor (FET) with an aminooxy-terminated silane coupling reagent, highlighting the utility of surface-modified inorganic nanomaterials in biosensing applications [45]. Moreover, A dual pH- and light-controlled drug delivery system was developed using surface-modified mesoporous silica NPs, demonstrating precise drug release and enhanced therapeutic efficacy in cancer treatment [46]. Beyond surface modification, organic–inorganic NPs can be engineered through the self-assembly of block copolymers. Furthermore, inorganic NPs can be incorporated into polymer matrices to create bulk composite nanomaterials, such as functional hydrogels. A previous review provided a systematic analysis of hybrid nanomaterials [42].

Another category of composite-based nanomaterials is metal–organic frameworks (MOFs), which are constructed from coordination bonds between metal-containing nodes and multidentate organic bridging ligands. MOFs offer customizable compositions and topologies, as well as high porosity and larger Brunauer–Emmett–Teller (BET) surface areas compared to single-component nanostructures. These properties make MOFs highly suitable for applications in cancer therapy, drug delivery, and bioimaging, positioning them as promising platforms in the field of biomedicine [47, 48]. For instance, a recently reported hierarchical and size-adaptable MOF nanovehicle was designed to effectively cross biological barriers at the tissue, cellular, and nuclear levels, enabling efficient nucleus-targeted delivery of doxorubicin (DOX) [49]. Additionally, a newly developed MOF/sulfonated hyaluronic acid composite coating was applied to the surface of magnesium alloy vascular stents, enhancing their biocompatibility and corrosion resistance, which is essential for improving the performance and longevity of vascular implants [48].

Given their unique physicochemical properties, composite-based nanomaterials have become essential in advancing pharmaceuticals, targeted drug delivery, biosensors, medical imaging, and next-generation medical devices, driving innovation in more effective and personalised therapies.

### Healthcare applications of nanotechnology

**Fig. 3:**
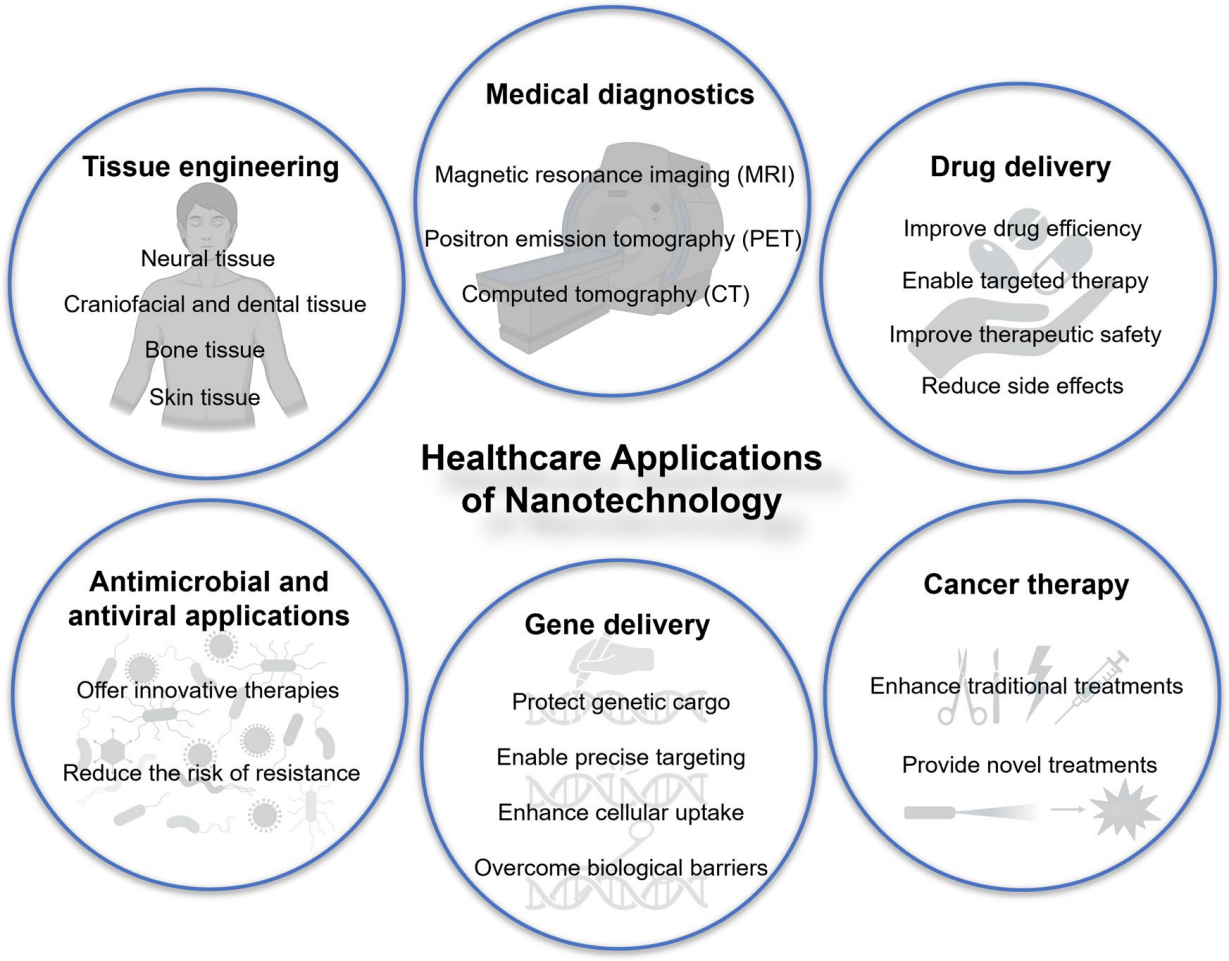
Applications of nanotechnology in healthcare. These applications involve a number of medical fields such as diagnostics, tissue engineering, drug delivery, gene delivery, cancer therapy, antimicrobial and antiviral applications

Nanotechnology has emerged as a promising field with significant potential for advancements in various healthcare and biomedical applications. Considering the extensive research conducted in this field and the numerous comprehensive reviews available, our focus will primarily be on providing concise descriptions of several mainstream applications of nanotechnology in recent years (Fig. 3).

#### Medical diagnostics

The progress of nanotechnology has significantly influenced the improvement of imaging techniques, early detection, diagnosis, and prognosis of diseases, enhancing existing clinically relevant technologies [2]. The field of diagnostic sciences has incorporated nanodevices to achieve early and swift disease identification, leading to subsequent medical procedural recommendations [5]. The potential of nanotechnology to transform healthcare diagnostics lies in its ability to enhance the precision, sensitivity, and speed of medical tests. One of the profound applications includes nanoparticle-based diagnostic imaging. The unique biophysical properties of NPs enable their attachment to specific biomarkers, thereby improving imaging modalities like magnetic resonance imaging (MRI), computed tomography (CT) scans, and positron emission tomography (PET) scans [2].

##### Magnetic resonance imaging (MRI)

**Fig. 4:**
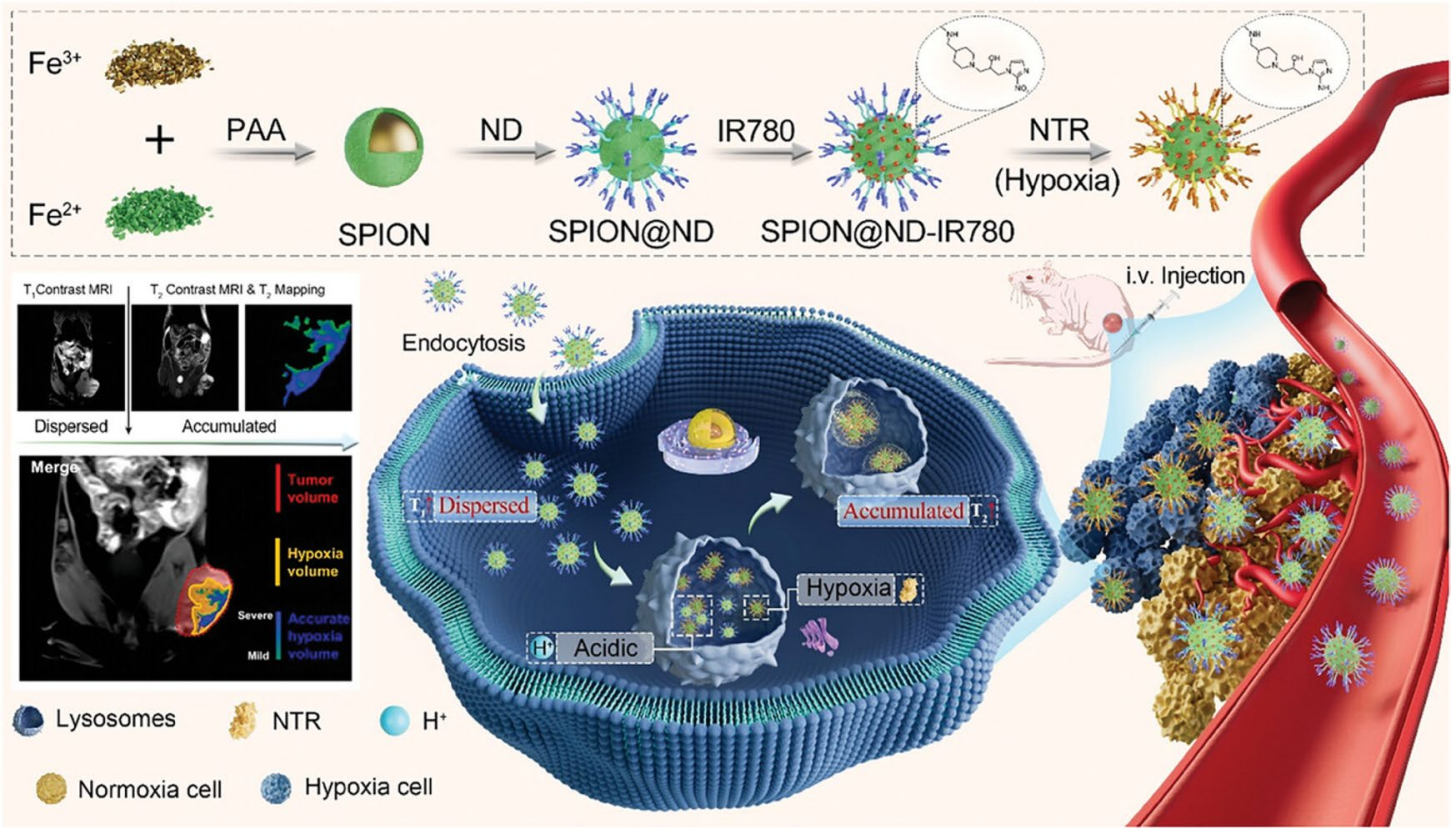
Schematic representation of SPION@ND-IR780, a tri-modal hypoxia imaging nanoprobe designed for precise tumour hypoxia imaging and the streamlined construction of hypoxia-guided biological target volumes (BTVs). The nanoprobe exhibits dual-stimuli responsive characteristics: hypoxia sensitivity, which facilitates nanoprobe accumulation, and acidic pH sensitivity, which induces nanoprobe aggregation. Adapted with permission from Wu et al*.* [54]

MRI uses strong magnetic fields and radio waves to create detailed images of soft tissues by exploiting the behaviour of hydrogen atoms in the body. However, traditional MRI has limitations in contrast resolution, making it challenging to distinguish between different soft tissue types and detect small or early-stage lesions [50]. Paramagnetic or superparamagnetic nanomaterials enhance MRI contrast by increasing interactions between water protons and paramagnetic centers, accelerating energy transfer and shortening spin–lattice (T1) or spin–spin (T2) relaxation times, thereby improving signal intensity and image clarity [51]. These nanomaterials encompass paramagnetic complexes based on gadolinium (Gd) ions or manganese (Mn) ions, as well as superparamagnetic iron oxide NPs (SPIONs), all of which are widely used as contrast agents in MRI [50]. Surface functionalisation can further improve their biocompatibility, prolongs circulation time, and enables targeted imaging, increasing both specificity and sensitivity [52]. Recent advancements include the development of pH-activatable manganese carbonate (MnCO_3_) NPs that enhance T1-weighted MRI specifically in acidic tumour environments, significantly improving the ability to distinguish malignant from benign tissues and enabling more accurate detection of tumour malignancy and metastasis [53]. Furthermore, a novel hypoxia-responsive tri-modal MRI nanoprobe (SPION@ND-IR780) encompassing T1, T2, and T2 mapping capabilities has been recently developed for enhanced imaging of hypoxic tumour regions in breast cancer [54]. This nanoprobe undergoes structural transformations under hypoxic conditions, thereby significantly improving imaging accuracy and resolution. The precise visualisation of hypoxic areas facilitated by this system enables more targeted and effective radiotherapy interventions, addressing issues of tumour radioresistance (Fig. 4).

##### Computed tomography (CT)

CT imaging relies on X-ray attenuation to produce detailed images, making it crucial for diagnosing various medical conditions. Traditional small-molecule iodine-based contrast agents, such as iohexol and iodixanol, while effective, are constrained by rapid metabolic clearance, suboptimal targeting specificity, and the risk of adverse effects, including nephrotoxicity and allergic reactions, which limit their efficacy, especially in enhancing soft tissue contrast. Nanotechnology has introduced advanced contrast agents, including NPs like Au NPs, which offer improved stability, targeting, and biocompatibility. These nano-contrast agents can enhance imaging precision, reduce side effects, and improve the overall diagnostic capability of CT imaging by leveraging properties such as enhanced permeability and retention (EPR) and surface modifiability [55].

**Fig. 5:**
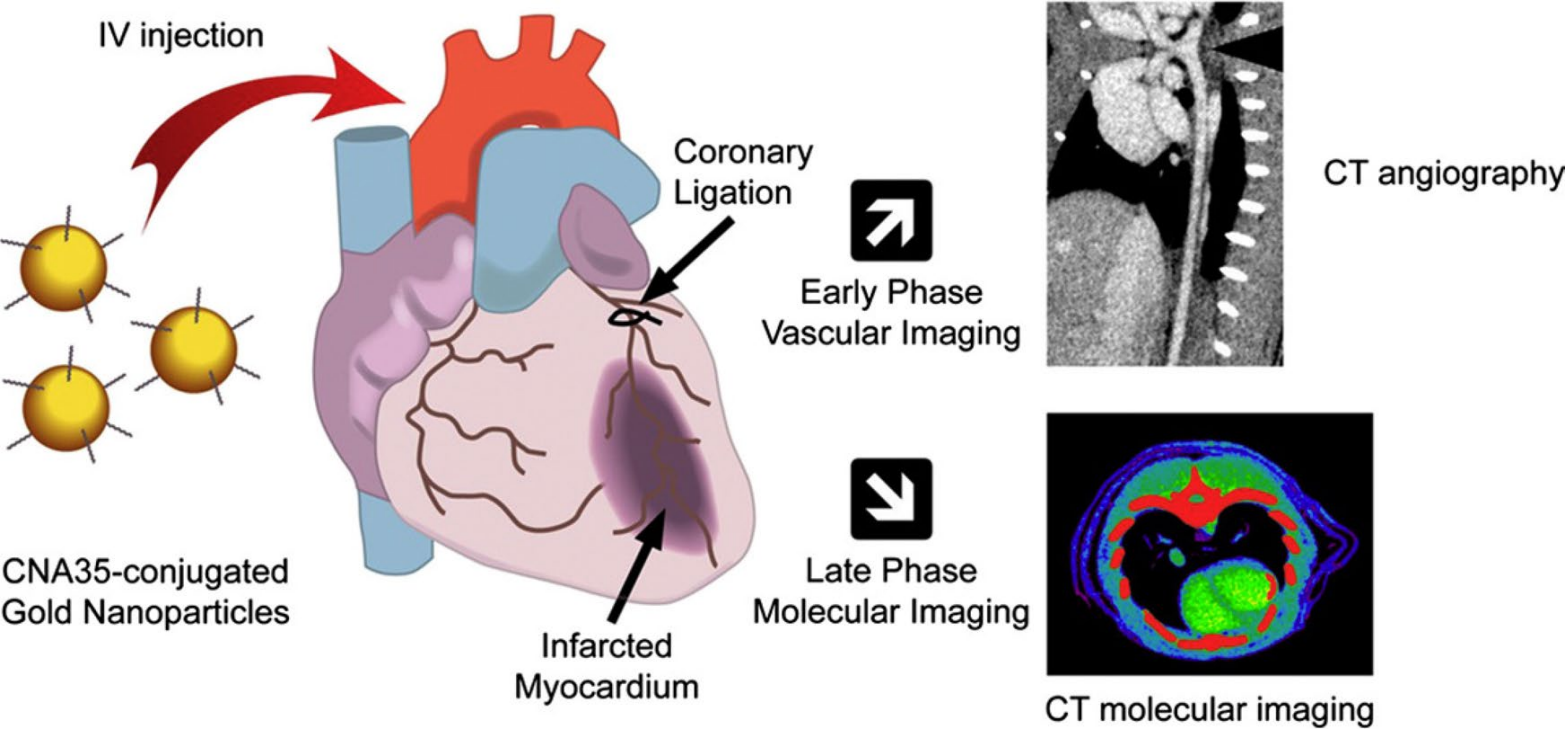
CNA35-conjugated Au NPs were explored as CT contrast agents for vascular and molecular imaging. Early phase imaging, within the first hour post-injection, showed uniform and stable blood pool enhancement. By six hours, after clearance of circulating NPs, late phase imaging revealed specific localisation and enhancement of myocardial scars. This provides essential diagnostic information on vascular lesions and scar burden, aiding in risk stratification and management of coronary artery disease. Adapted with permission from Kee and Danila [56]

For example, Au NPs functionalised with collagen-binding adhesion protein 35 (CNA35) have shown significant potential in improving CT imaging of myocardial scars [56]. This approach allows for prolonged blood pool enhancement and specific targeting of collagen within scar tissue, providing clearer and more detailed images compared to conventional iodine-based agents. In a study involving a rat model of myocardial infarction, CNA35-conjugated AuNPs provided substantial signal enhancement in the scar tissue up to six hours post-injection, highlighting their potential for more precise cardiovascular diagnostics (Fig. 5).

Additionally, a recent study introduced a novel self-assembled CT contrast agent, BioDHU-CT NPs, designed to leverage size aggregation for improved tumour imaging [57]. They are composed of a biotin polyethylene glycol (Biotin-PEG) segment for hydrophilic properties and active targeting, an ROS-responsive group for rapid reactivity to reactive oxygen species, and an iodine-containing component modified with tetraphenylethene (TPE) for enhanced imaging capabilities. These NPs remain small for effective tumour penetration but rapidly aggregate upon activation by ROS in the tumour microenvironment. This size transformation enhances retention and extends the imaging window, significantly improving the contrast and clarity of tumour images in CT scans. The study demonstrated that these NPs provide more sustained and targeted imaging, offering a promising alternative to conventional contrast agents for tumour diagnostics.

##### Positron emission tomography (PET)

PET imaging is a diagnostic tool that enables the visualisation of metabolic processes within the body. By detecting pairs of gamma rays emitted indirectly by a positron-emitting radiotracer, PET provides detailed images of the function of tissues and organs. Nevertheless, traditional PET imaging faces certain limitations, including low spatial resolution and sensitivity, which hinder its ability to detect small or early-stage tumours. Moreover, the radiotracers commonly used in conventional PET tend to accumulate non-specifically in tissues, resulting in background noise that can obscure critical details.

**Fig. 6:**
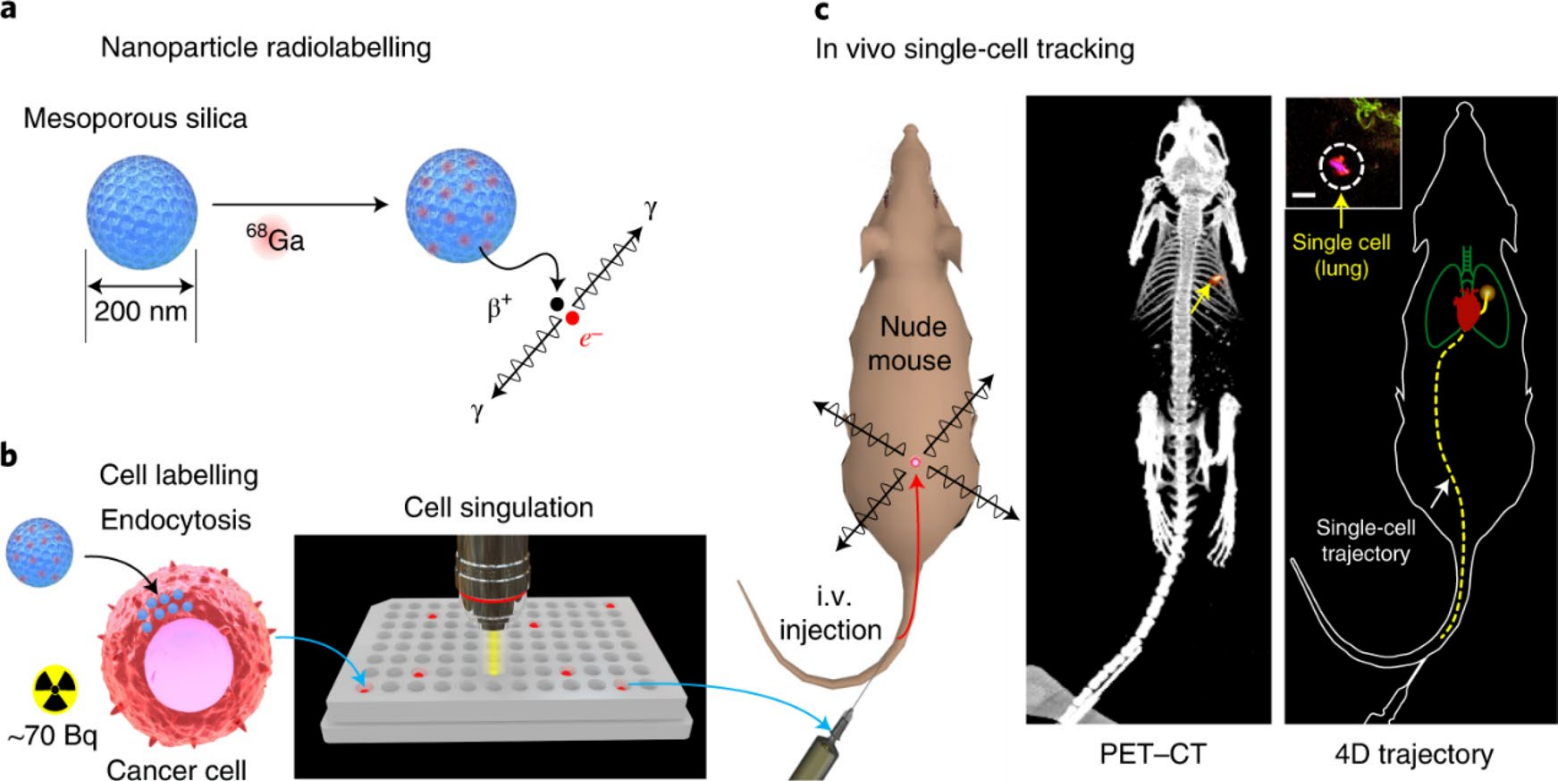
**a** Mesoporous silica nanoparticles (MSNs) concentrate ⁶⁸Ga from a clinical PET generator. **b** The NPs are then loaded into cells, achieving up to 100 Bq per cell, a fraction of a standard PET dose. **c** These isolated single cells are administered into mice, with gamma rays emitted from each cell detected by a small-animal PET scanner. The captured data is processed to estimate the cell's location in real time. In this example, single human breast cancer cells, administered intravenously, were detected in the lungs (yellow arrows), as confirmed by ex vivo analysis (inset; scale bar, 20 µm). Adapted with permission from Pratx et al*.* [61]

Similarly, PET imaging has seen significant advancements with the use of nanomaterials. NPs can be engineered to carry PET radiotracers, which accumulate in specific tissues, providing high-contrast images that are crucial for early detection of tumours and monitoring of metastatic spread [58]. By addressing the limitations of traditional PET, NPs enable more precise and sensitive imaging. For instance, nanoscale MOFs have been developed that are intrinsically radioactive, such as the UiO-66 MOF radiolabeled with zirconium-89 [59]. It combines PET isotope zirconium-89 with 1,4-benzenedicarboxylate (BDC) and benzoic acid (BA) linkers, forming a highly porous structure. This porosity allows effective drug loading, while surface functionalisation with PEG and tumour-targeting peptides enhances stability, biocompatibility, and enables targeted imaging and drug delivery. In addition, nano-coordination polymers (NCPs) offer another promising example in PET imaging. Zr-P1 NCPs, composed of zirconium ions and tetrakis (4-carboxyphenyl)ethylene ligands, are radiolabeled with zirconium-89. Functionalised with PEG and cyclo(Arg-Gly-Asp-d-Phe-Cys) peptides, these NCPs target integrin αvβ3 in tumours, enhancing biocompatibility and specificity [60]. This design improves PET imaging by reducing background noise and providing clearer, more precise tumour visualisation. Moreover, in the domain of single-cell tracking, radiolabeled NPs, such as ^68^Ga radioisotope labeled mesoporous silica NPs, have been used to track individual cancer cells though PET (Fig. 6). This technique helps study cell trafficking, immune cell movement in cancer immunotherapy, and cell distribution post-transplantation [61].

Beyond these key medical imaging techniques (MRI, CT, and PET), nanomaterials are also being increasingly used in other advanced imaging modalities such as fluorescence imaging, photoacoustic imaging (PAI), and ultrasound imaging. A recent review provides a systematic study of nanomaterials used in these technologies, highlighting their potential to significantly enhance diagnostic precision and expand the capabilities of medical imaging [62]. As research advances, these innovative nanomaterials are poised to revolutionise healthcare by enabling more accurate and personalised diagnostic solutions.

Moreover, the impact of nanotechnology extends beyond imaging, notably enhancing the performance of biosensors. By increasing sensitivity and dramatically lowering detection limits by several orders of magnitude, nanomaterials facilitate the detection of trace biomolecules in bodily fluids such as blood and urine, which is critical for early diagnosis and effective disease management. These materials are integral in the immobilisation of biomolecules, signal amplification, and serving as mediators, electroactive species, and detection nanoprobes. Diverse nanomaterials, including NPs, nanowires, nanofilms, QDs, nanocrystals, nanorods, nanobelts, nanotubes, embedded nanostructures, and self-assembled nanomaterials, have been successfully integrated into biosensors [3]. Their use is tailored to the specific conduction mechanisms of the biosensors, whether electrochemical, optical, or thermoelectric, further advancing the field of biomedical diagnostics and treatment strategies [63, 64]. The field is vast and continually evolving, with numerous studies and reviews offering more comprehensive insights into the latest developments [65–70].

#### Tissue engineering

**Fig. 7:**
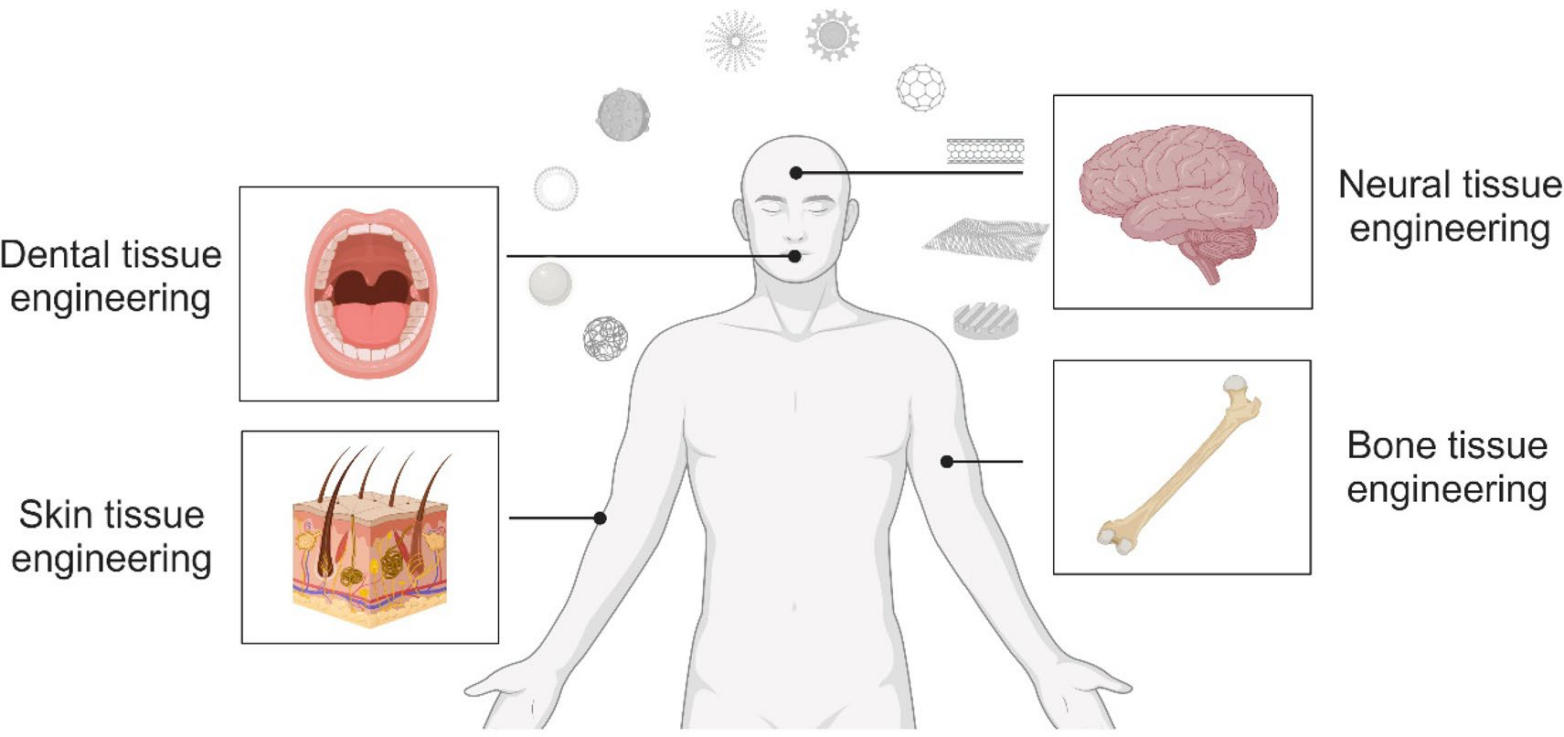
Applications of nanomaterials in tissue engineering

Tissue engineering combines biology, engineering, and materials science to develop substitutes that restore or enhance tissue function. This involves using scaffolds, cells, and bioactive molecules to create functional tissues for medical applications, aiming to repair damaged tissues and reduce the need for organ transplants [71]. Key research areas include neural, dental, bone, and skin tissue engineering (Fig. 7) due to the high incidence of injuries and diseases affecting these tissues, as well as their complex structures and functions [72]. These areas have significant clinical demand and potential for improving patients' quality of life. Advancements in nanotechnology have significantly improved therapeutic outcomes in these fields by enhancing biomaterials properties, cell interactions, and tissue regeneration processes.

##### Neural tissue engineering

**Fig. 8:**
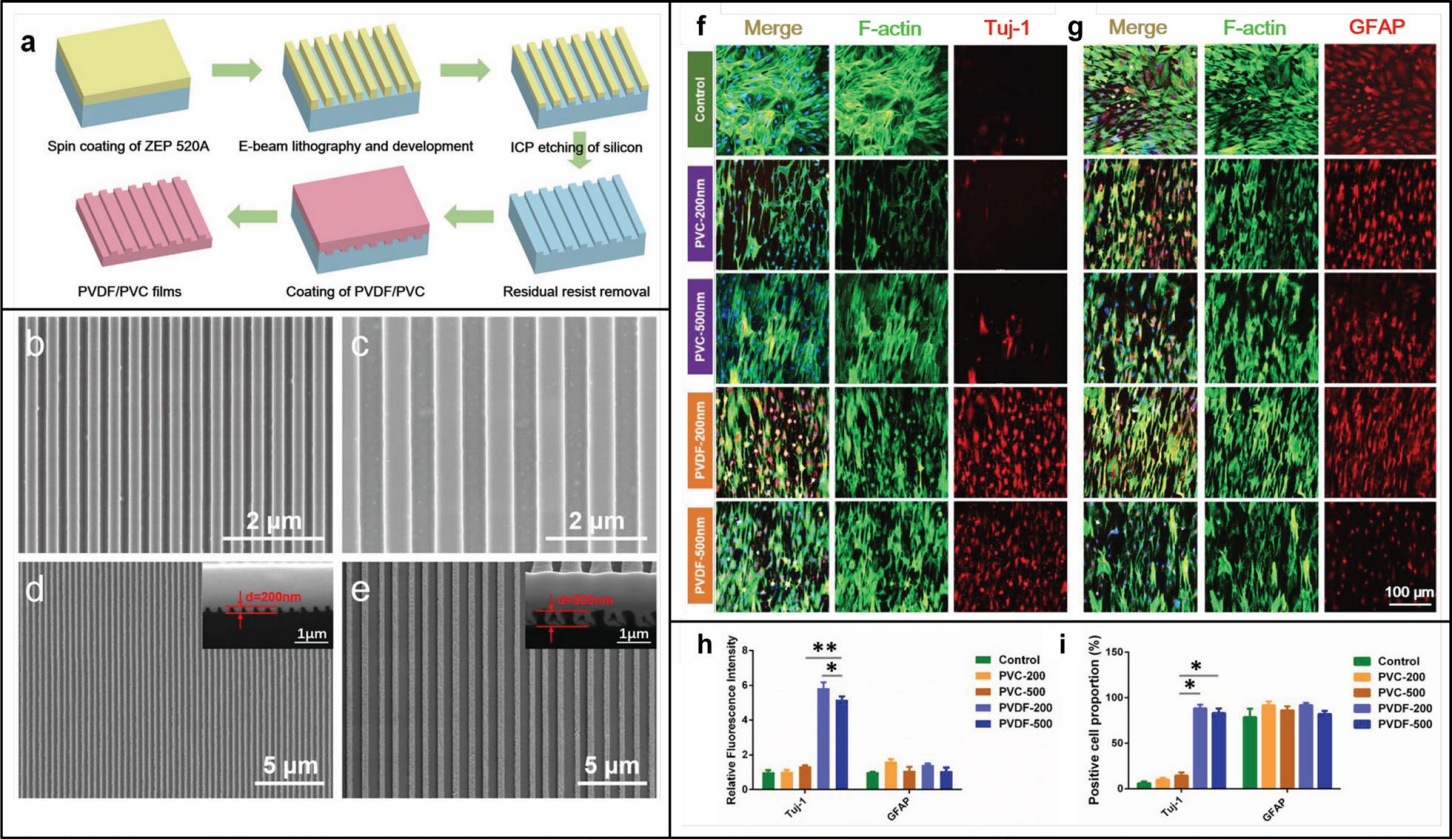
**a** The schematic illustration of fabrication procedure of PVDF or non-piezoelectric polyvinyl chloride (PVC) films. SEM images of the silicon molds with repetition period of **b** 400 nm (ridge, groove, and height were all 200 nm) and **c** 1000 nm (ridge, groove, and height were all 500 nm). SEM images of **d** the PVDF-200 (ridge, groove, and height were all 200 nm) and **e** the PVDF-500 (ridge, groove, and height were all 500 nm). **f** Immunofluorescent staining of the neuron specific maker Tuj-1 and **g** a neurogliocyte specific maker GFAP after 7 days culture. The cell nuclei were stained with DAPI (blue) and F-actin was stained with phalloidin-Alexa Fluor 488 (green). Tuj-1 and GFAP were immunostained, respectively (red). **h** Statistical analysis of the fluorescence intensity of Tuj-1 and GFAP. **i** Statistical analysis of the percentage of Tuj-1 positive cells and GFAP positive cells. Adapted with permission from Zhang et al*.* [79]

Neural engineering has benefited from the use of nanomaterials, demonstrating encouraging outcomes by facilitating cell adhesion and proliferation, and promoting neuronal cell differentiation, thus augmenting neuron regeneration. Currently, nanomaterials are expected to be used for the treatment of neurological injuries. Manipulation of the physicochemical properties of nanomaterials can prevent and/or treat neurodegeneration [73]. For example, biodegradable natural polymers, SPIONs, carbon-based nanomaterials, and silica nanostructures have demonstrated exceptional properties and superior efficacy in facilitating neural cell differentiation and outgrowth [73]. Composite-based nanomaterial of graphene and polyethylene terephthalate (PET) are employed in non-toxic, non-contact electrical stimulation to enhance cell-to-cell coupling in human neuroblastoma [74]. Magnetic nanomaterials exploit magneto-responsive effects in response to an external magnetic field, allowing for the modulation of neuronal cell activity to either activate or inhibit, aiming to treat neurological diseases such as Alzheimer's disease and Parkinson's disease [75–77]. In addition, studies have demonstrated the ability of metallic NPs like gold, iron, and cerium to ameliorate adverse pathological changes associated with spinal cord injury [78]. Furthermore, innovative approaches using nanomaterials combined with other bioactive substances have shown synergistic effects in neural tissue engineering. Zhang et al*.* fabricated two piezoelectric polyvinylidene fluoride (PVDF) nanostripe array structures, with ridge, groove, and height dimensions of either 200 or 500 nm, designed as scaffold surfaces for neural tissue engineering [79]. Their results revealed that the combination of piezoelectricity and nanogeometry favorably influenced neuron-like differentiation in both cell morphology and gene/protein expression, promising to promote nerve repair and regeneration (Fig. 8). In addition to the above, NPs can be functionalised to facilitate the crossing of the blood–brain barrier for targeted brain drug delivery [80].

In conclusion, nanotechnology holds significant potential for indispensable applications in neural tissue engineering.

##### Dental tissue engineering

Nanotechnology holds significant promise for advancing oral health in the field of craniofacial and dental tissue engineering, making it a prominent area for growth and potential enhancement of the quality of life of patients [81].

**Fig. 9:**
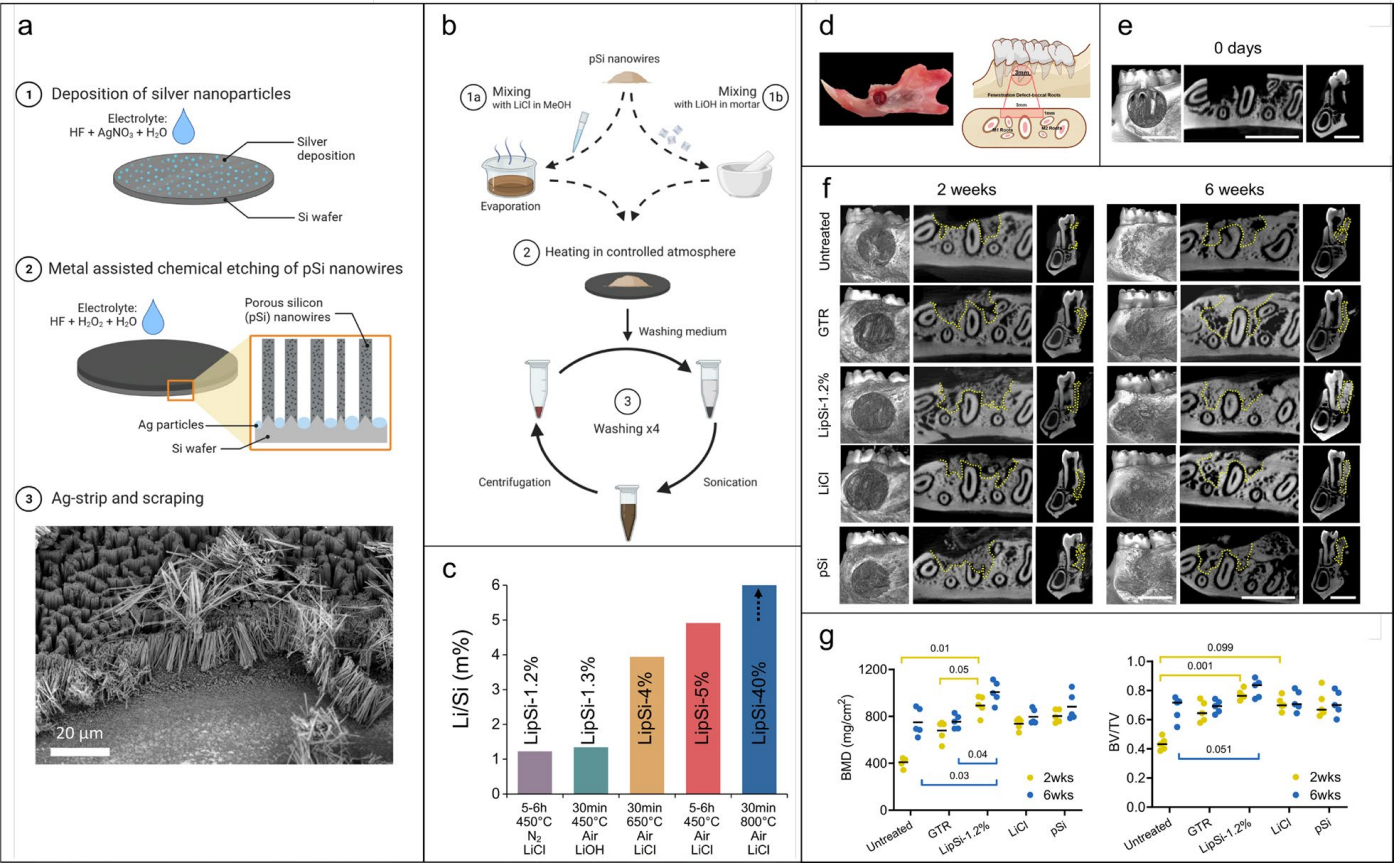
**a** Schematic of the metal assisted electrochemical etching (MACE) process used to generate porous silicon nanowires (pSiNs). **b** Schematic of the lithiation process for porous silicon nanowires. **c** Quantification of Li/Si ratio of LipSiNs of fully dissolved nanowires as a function lithium precursor, lithiation temperature, time and atmosphere. **d** The model of periodontal fenestration defect (standardised with 3 mm in length, 3 mm in height and < 1 mm in deep). **e** Micro-computed tomography (μCT) scans of rat mandibles at day 0. Analysis was performed on 5 animals. **f** μCT scans of rat mandibles showing regeneration of periodontal defects 2 weeks and 6-weeks post-operative with lithium chloride, commercial guided tissue regeneration membrane (GTR, BioGide^®^), pSi, and LipSi − 1.2%; serves as baseline comparison. The dotted yellow line outlines the newly formed bone. **g** μCT analysis for the quantification of bone volume over total volume (BV/TV) and bone mineral density (BMD). Adapted with permission from Kaasalainen et al*.* [84]

The involvement of periodontal diseases in the hard and soft tissues surrounding the teeth results in gum diseases, bone loss, and, in more severe cases, tooth loss. Nanomaterials are expected to offer innovative, nonsurgical alternatives for addressing periodontal disease, exhibiting minimal side effects yet high efficiency. The post-subgingival injection of biodegradable polydopamine NPs has been shown to be capable of efficiently remove excessive reactive oxygen species (ROS) in vivo and decrease local periodontal inflammation [82]. Au NPs with surface-anchored chiral amino acids (L-cysteine) have been reported to be able to regulate cellular behaviour through chiral effects and autophagy, thereby stimulating periodontal regeneration [83]. In addition, lithiated porous silicon nanowires (LipSiNs) have been recently shown to combine osteogenic, cementogenic and Wnt/β-catenin stimuli, leading to the regeneration of bone, cementum, and periodontal ligament fibers in a murine model of periodontal defect (Fig. 9). This regenerative potential proved to be significantly higher compared to previously studied materials, including lithium chloride, porous silicon nanowires, lithium-substituted bioglass, and a commercial guided tissue regeneration membrane (GTR, BioGide^®^) used for the treatment of periodontitis [84].

Oral and cranio-maxillofacial diseases are complications of the soft and hard tissues of the craniofacial, face and dental arches induced by physical, chemical, microbiological factors, and systemic disorders. These conditions include craniofacial defects and conditions such as head and neck tumours, craniosynostosis, dental implant failures, and osseous malunion. They often result in facial deformities and bone defects that require surgical intervention to correct normal facial features. Zirconium dioxide (ZrO_2_) NPs have been investigated for improving the mechanical properties and colour instability of maxillofacial prostheses such as silicone and silicone elastomers in order to enhance functionality and aesthetics [85, 86]. An electro-spun titanium oxide (TiO_2_)/hydroxyapatite (HA)/polyurethane (PU) nanocomposite fibre has been designed for use as an osseointegrated membrane to enhance new bone formation in oral and maxillofacial surgery [87].

Furthermore, incorporating nanotechnology, specifically antibacterial NPs, into endodontic materials can enhance their properties, preventing recurrent infections and improving the success rates of root canal treatments [88]. The incorporation of antimicrobial nano-coatings in orthodontic materials prevents the dental plaque formation around orthodontic appliances and prevents dental caries associated with orthodontic treatments [81]. Nanomaterials like TiO_2_ nanotubes, silver nanoparticles (Ag NPs), and graphene oxide have been employed to coat the surface of titanium dental implants, aiming to improve osseointegration, soft tissue integration, immunomodulation, antitumour and antibacterial properties [89].

Advances in nanomaterials, whether utilised independently or in conjunction with existing materials for oral medicine, offer innovative strategies for craniofacial and dental tissue engineering, thereby enhancing health, functionality, and aesthetics.

##### *Bone tissue engineering*

**Fig. 10:**
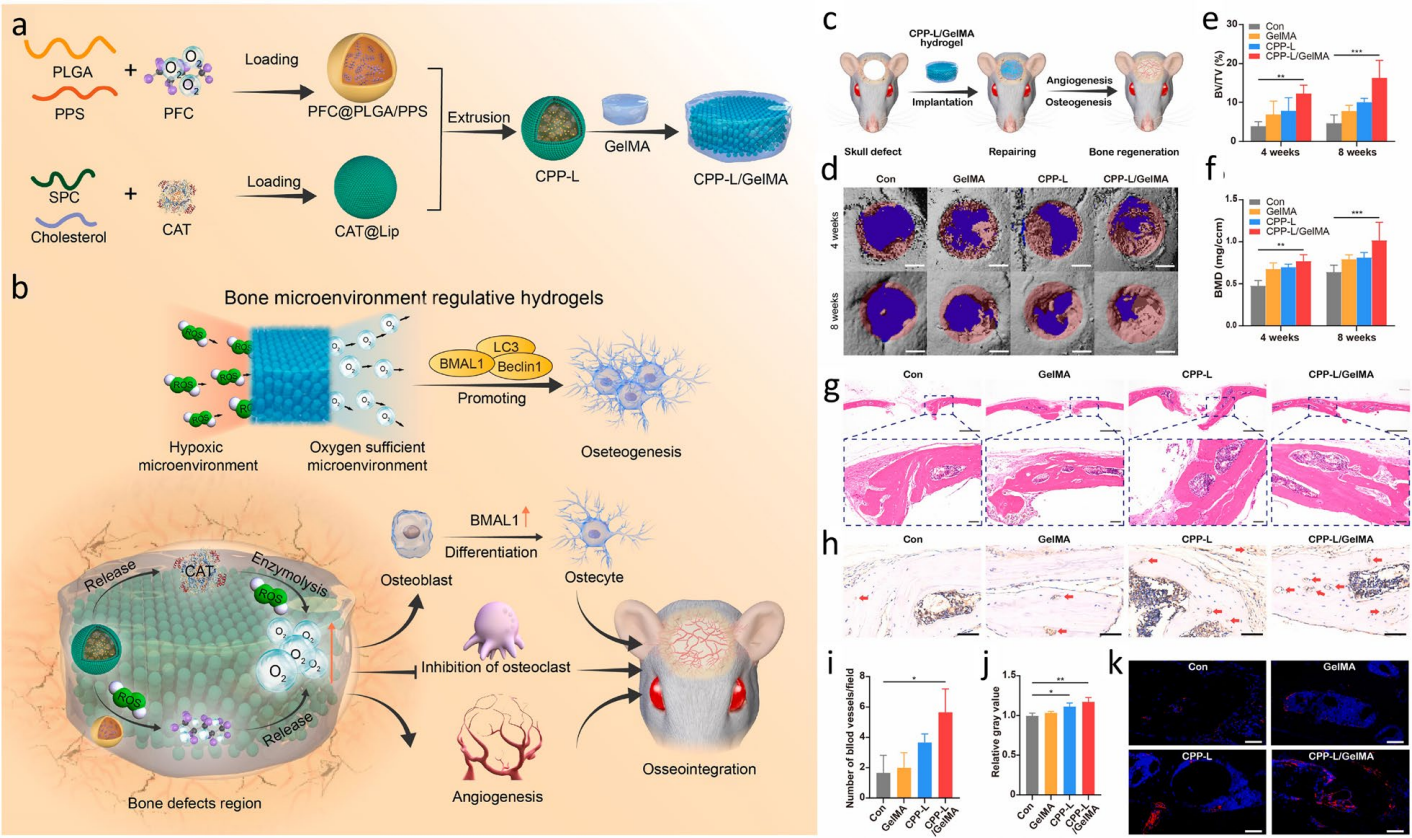
**a** Perfluorocarbon (PFC) is loaded into PLGA/poly (propylene sulphide) (PPS) NPs to form PFC@PLGA/PPS, which, along with CAT, is encapsulated in liposomes and GelMA hydrogel to create CPP-L/GelMA. **b** CPP-L/GelMA hydrogel reverses hypoxia in bone defects by releasing CAT and oxygen, promoting osteogenesis and neovascularisation while inhibiting osteoclastogenesis. **c** Schematic of hydrogel implantation in a mouse skull defect. **d** 3D micro-CT images of treated skull defects at 4 and 8 weeks. **e**, **f** Bone volume fraction and bone mineral density measurements. **g** H&E staining of skull defects at 8 weeks. **h**, **i** CD31 staining and vessel density analysis. **j**, **k** Flk-1 immunofluorescence staining and analysis. Significance: *p < 0.05, **p < 0.01, ***p < 0.005. Adapted with permission from Sun et al*.* [92]

Bone defects are the result of the compromised structural integrity of bones, primarily attributed to trauma and bone disease [90]. The integration of nanotechnology into strategies for bone tissue engineering offers benefits by guiding cellular differentiation toward osteogenesis and aiding in the repair of substantial bone defects. NPs play a crucial role in advancing both scaffold-free and scaffold-based tissue engineering methods for promoting osteogenesis and bone regeneration. They modulate inflammatory responses and signaling pathways related to osteogenesis, angiogenesis, and osteoclast activity to create an osteogenic niche. Additionally, NPs interact with biomolecules, extending their half-life and enhancing bioavailability, making them highly promising materials for promoting osteogenesis [91]. In bone tissue engineering, commonly used nanomaterials include metallic/metallic oxide NPs such as silver (Ag NP), strontium (Sr NP), magnesium oxide (MgO NP), cobalt ferrite (CoFe_2_O_4_ NP), among others. Additionally, calcium phosphate NPs including hydroxyapatite (HA) and tricalcium phosphate (TCP) are prevalent, alongside silicate-based NPs like bioactive silica-based glass particles (SBA2) and mesoporous silica NPs. Furthermore, polymeric NPs made of polymers such as poly(lactic acid) (PLA), poly(lactic acid) (PLA) nanofibers, poly(glycolic acid) (PGA), and poly(lactic-*co*-glycolide acid) (PLGA), chitosan, gelatin (Gel), and silk fibroin (SF) are commonly employed [90, 91]. For example, Sun et al*.* developed Gelatin Methacrylate (GelMA) hydrogels (CPP-L/GelMA) incorporating polymeric NPs to regulate the bone microenvironment by scavenging ROS and generating prolonged oxygen release [92]. These NPs, serving as carriers of catalase (CAT) and oxygen, improved oxygen supply to the bone defect area, promoted angiogenesis and osteogenesis, inhibited osteoclastogenesis, and ultimately facilitated bone regeneration (Fig. 10).

In addition, nano-surface modification of bone implants is widely adopted. Enhanced osteogenic capacity, modulation of macrophage-mediated inflammatory responses, and promotion of osteointegration are attainable through nano-surface modifications [93]. Various techniques, such as acid etching, sandblasting, laser modification, anodisation, micro-arc oxidation, hydrothermal treatment, chemical vapor deposition (CVD), atomic layer deposition (ALD), plasma-immersion ion implantation (PIII), and lithography, have also been employed to modify the surface topography of grafts [94–96].

These developments underscore the potential of nanomaterials to improve bone tissue engineering strategies, highlighting their promise in creating more effective bone repair solutions.

##### Skin tissue engineering

Nanotechnology has been extensively studied in skin tissue engineering. The objective of skin tissue engineering is to rebuild both the structural and functional elements of skin, with the goal of minimising scar formation and enhancing the overall quality of wound healing. Clinical approaches of wound management include techniques like hyperbaric oxygen therapy, debridement, negative wound pressure therapy, and the use of wound dressings [97].

Among these methods, the role of nanotechnology role is prominent in wound dressings. Nanocellulose, with its unique properties including enhanced absorbency and easier removal compared to traditional materials like gauze, is widely adopted in biomedical applications for treating skin diseases. Its nanoscale morphology mimics the native extracellular matrix, rendering it a favorable substrate for skin cell adhesion and growth [98]. Combining nanocellulose with chitosan, poly (*N*-isopropylacrylamide) (PNIPAAm), polyvinyl alcohol (PVA), magnetic NPs, lactoferrin, collagen, and alginate in nanocomposites has demonstrated further improved effectiveness in repairing skin tissue [99].

**Fig. 11:**
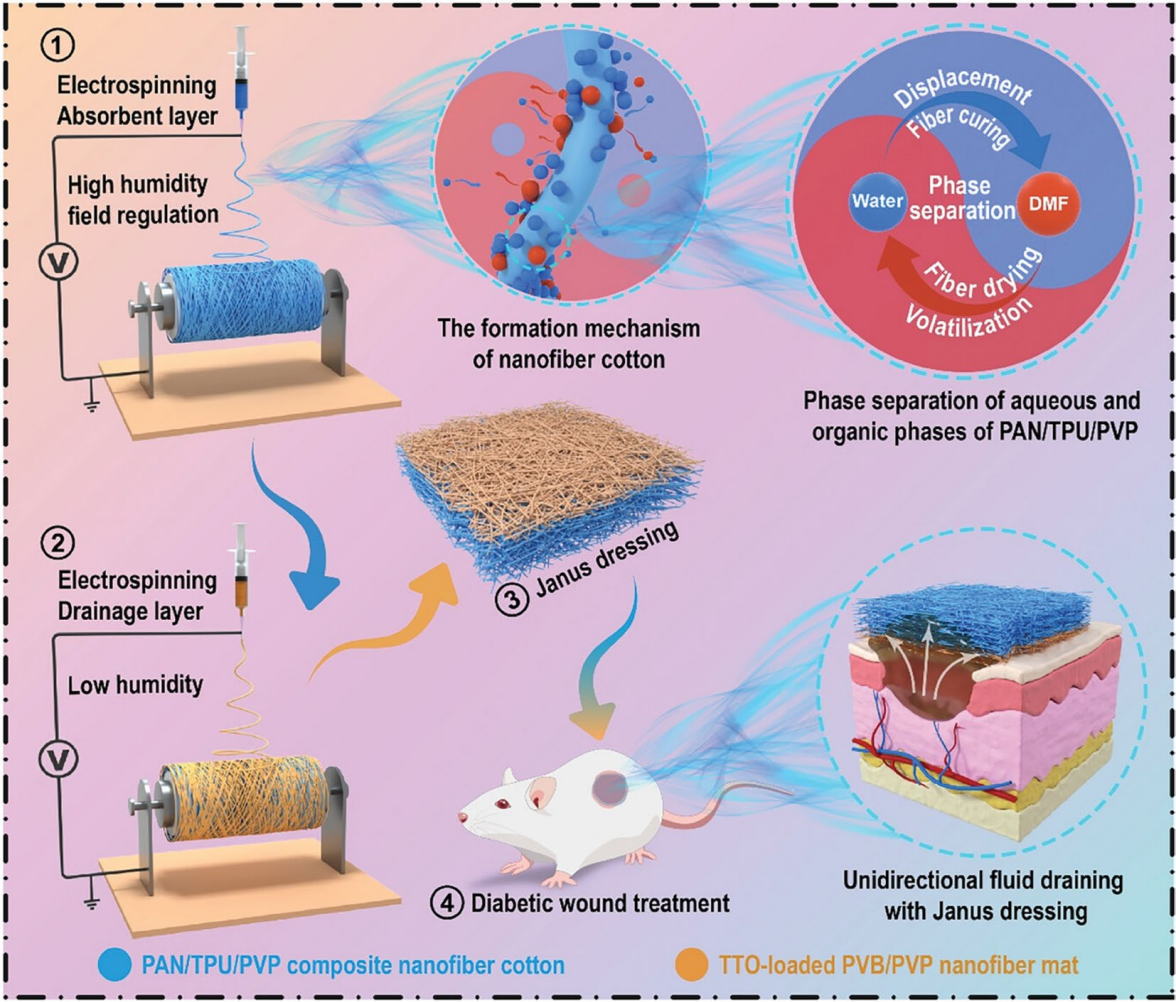
Schematic preparation of a superabsorbent Janus dressing with polyvinylpyrrolidone-induced (PVP-induced) self-pumping for diabetic wound treatment by unidirectional draining excessive exudate. Adapted with permission from Zhou et al*.* [102]

Autologous skin grafting is the “gold standard” of treatment for full-thickness injuries. However, due to the limitations of donor skin, the development of other tissue-engineered skin substitutes (such as skin regeneration scaffolds) is becoming increasingly important. Metal, ceramic and carbon-based nanomaterials have been introduced into scaffolds to reduce inflammation and enhance antimicrobial properties. Biomimicking scaffolds containing polymer nanofibres are able to recapitulate the native skin architecture and enhance cell proliferation [97]. Additionally, dual-faced polymeric nanofibers, or Janus nanofibers, can be engineered to integrate various materials and functionalities, yielding synergistic physicochemical effects and presenting a promising material for skin tissue engineering [100, 101]. Zhou et al*.* introduced a self-pumping Janus dressing with dual layers to enhance wound exudate management and accelerate healing [102]. This dressing consists of a hydrophobic drainage layer and a superabsorbent nanofiber layer, which work together to provide unidirectional fluid drainage and promote diabetic wound healing (Fig. 11).

In summary, nanotechnology, through the development of advanced wound dressings and biomimetic scaffolds, plays a crucial role in enhancing skin tissue engineering by improving wound healing, minimising scarring, and supporting cell growth and proliferation.

#### Drug delivery

Nanotechnology has transformed the field of drug delivery by providing precise and efficient drug-targeted delivery, smart-responsive drug release, and extraordinary in vivo stability, resulting in improved therapeutic efficacy, higher bioavailability, and reduced side effects [5]. A wide array of organic and inorganic nanomaterials has been developed to address various physicochemical challenges associated with drugs, including low solubility, stability, off-target deposition, and limited penetration across biological barriers [103].

The efficacy of NPs as drug carriers varies depending on factors like their shape, size, and other inherent biophysical or chemical characteristics [104]. Additionally, the method of drug loading, whether through conjugation or adsorption, plays a significant role in determining their performance. Conjugation involves the formation of covalent bonds between drug molecules and the surface of NPs, which can enhance drug stability, provide controlled release, and improve targeted delivery to specific tissues or cells [105]. In contrast, adsorption relies on non-covalent interactions, such as hydrophobic interactions or electrostatic forces, to physically load drugs onto NPs. While this approach is more straightforward and cost-effective, it may offer less stability and control over drug release compared to conjugation [106].

##### Enhancing active and passive transport

In nanomaterial drug delivery systems, the effectiveness of drug-targeted delivery can be further enhanced by employing both passive and active transport mechanisms, which complement the inherent characteristics of NPs as drug carriers [107]. Passive drug transport leverages the physicochemical properties of the nanocarriers and the specific physiological environment of the target tissue [108]. For instance, through the EPR effect, nanomedicines can passively accumulate at tumour sites, taking advantage of the leaky vasculature commonly found in tumours [107]. In contrast, active drug transport involves more precise interactions between nanomedicines and specific cellular targets, such as ligand-receptor binding, pH-responsive release, or the use of cell-penetrating peptides, which enable the drugs to efficiently target and enter specific cells [109]. These active mechanisms often work synergistically with passive transport to enhance the overall efficiency, specificity, and safety of drug delivery, particularly when combined with the strategic attachment methods of drug loading methods discussed earlier.

Doxil^®^, approved by the United States Food and Drug Administration (FDA) in 1995, marked the formal introduction of nanoparticle-based drug delivery formulations into clinical use. Traditional nanomaterial drug delivery systems, represented by Doxil^®^, have successfully achieved drug targeting and improved bioavailability. However, challenges such as drug toxicity and long-term biosafety remain [110]. Doxil^®^ is a PEGylated liposomal formulation of doxorubicin that employs the EPR effect as a passive targeting mechanism, along with surface PEG modification. The hydrophilic PEG shell protects the drug from degradation by serum components and prevents opsonisation by the complement system, thereby avoiding rapid clearance by the mononuclear phagocytic system (MPS) and extending circulation time [111]. While these modifications enhance bioavailability and drug delivery, the reliance on the passive EPR effect alone results in low targeting efficiency, which can still lead to significant cardiotoxicity, similar to that of the original DOX [112].

To address the shortcomings, novel nanomaterial drug delivery platforms are being developed to achieve precise drug delivery by integrating both active and passive targeting mechanisms. This approach aims to reduce drug toxicity while enhancing therapeutic efficacy. Abraxane^®^, a nanoparticle-based formulation of paclitaxel bound to albumin, exemplifies this strategy and was approved by the FDA in 2005 [113, 114]. Albumin, a natural blood protein, contains hydrophobic pockets that can bind paclitaxel through hydrophobic interactions. Serving as a natural carrier, albumin increases paclitaxel's solubility in the bloodstream and offers advantages such as biodegradability, non-toxicity, and non-immunogenicity. Tumour tissues overexpress secreted protein acidic and rich in cysteine (SPARC), which functions similarly to albumin receptors by selectively binding albumin and accumulating it in tumour cells. Additionally, the high demand for albumin in tumour tissues further facilitates the concentration of drug-loaded albumin NPs at the tumour site. These characteristics enable albumin-based nanoparticle formulations to significantly increase local drug concentrations in tumour tissues, thereby reducing systemic toxicity and improving patient tolerance [115].

Additionally, numerous nanoparticle-based formulations that combine active and passive targeting mechanisms are currently undergoing clinical trials. For example, MBP-426 is a novel nano drug delivery system in which oxaliplatin is encapsulated within *N*-glutaryl phosphatidylethanolamine (NGPE) liposomes and bound to transferrin [116]. Transferrin acts as a targeting carrier by binding to transferrin receptors, which are highly expressed in tumour tissues, thereby efficiently delivering the drug to cancer cells [117]. Phase I clinical trials of MBP-426 have been completed, and the formulation has since advanced to a Phase Ib/II clinical trial in second-line patients with gastric, gastroesophageal, or esophageal adenocarcinoma [118].

**Fig. 12:**
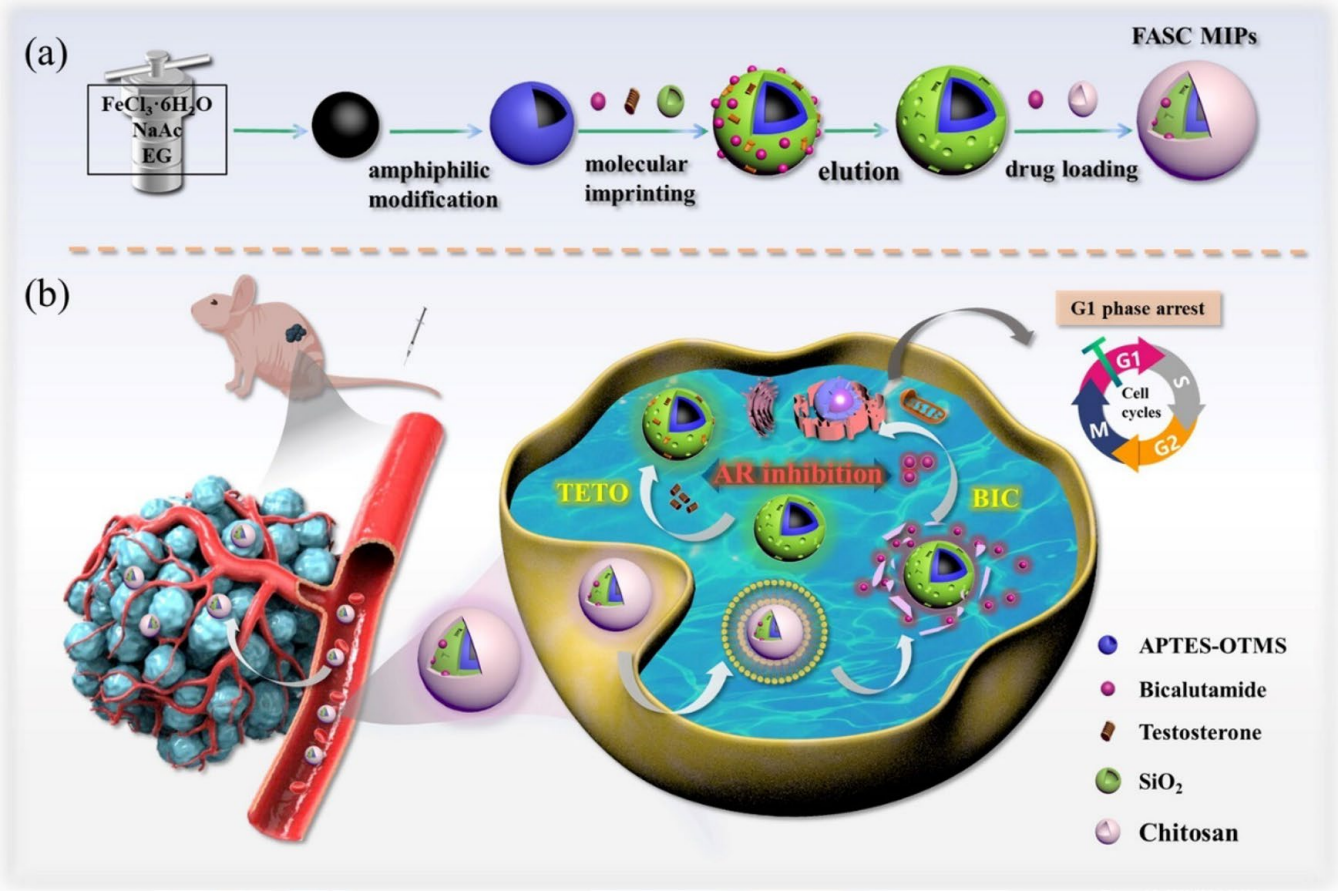
**a** Schematic illustration of the preparation route for FASC MIPs. **b** Strategy for achieving synergistic treatment of prostate tumours in vivo. Adapted with permission from Liu et al*.* [124]

Recent studies have shown that molecularly imprinted polymeric nanoparticles (MIP NPs) are promising candidates for drug delivery systems that integrate both active and passive targeting mechanisms. MIP NPs have been designed with specific and complementary binding sites for drug molecules (referred to as “templates”) within their polymer matrix. These sites enable MIP NPs to recognise and load drugs or other templates with antibody-like affinity and selectivity, through either covalent or non-covalent binding [119–121]. This ability allows MIP NPs to improve the precision of drug delivery and release. Furthermore, the development of dual-imprinted polymers enables both drug loading and in vivo targeting, facilitating active targeted delivery and responsive release of drugs [122, 123]. For example, Liu et al*.* developed a novel pH-responsive core–shell MIP NPs (FASC MIPs) for prostate cancer treatment [124]. This formulation uses superparamagnetic tetraoxide NPs as the core material. The surface of these NPs was modified to create two types of imprinted cavities: one designed to sequester free testosterone (TETO) from solid tumours and another loaded with the anti-androgen drug bicalutamide (BIC). The NPs were further coated with pH-responsive chitosan, which facilitates targeted drug release in the acidic tumour microenvironment (Fig. 12). The FASC MIPs demonstrated synergistic antitumour effects by specifically targeting TETO through the imprinted cavities and releasing BIC in response to the acidic conditions, effectively inhibiting prostate cancer cells growth both in vitro and in vivo.

Moreover, the high stability and robustness of MIP NPs protect the loaded drug molecules from complex biological environments, such as those in the gastrointestinal tract, making them suitable for various applications, including sustained transdermal formulations, therapeutic contact lenses, and oral formulations for protein delivery [122].

**Fig. 13:**
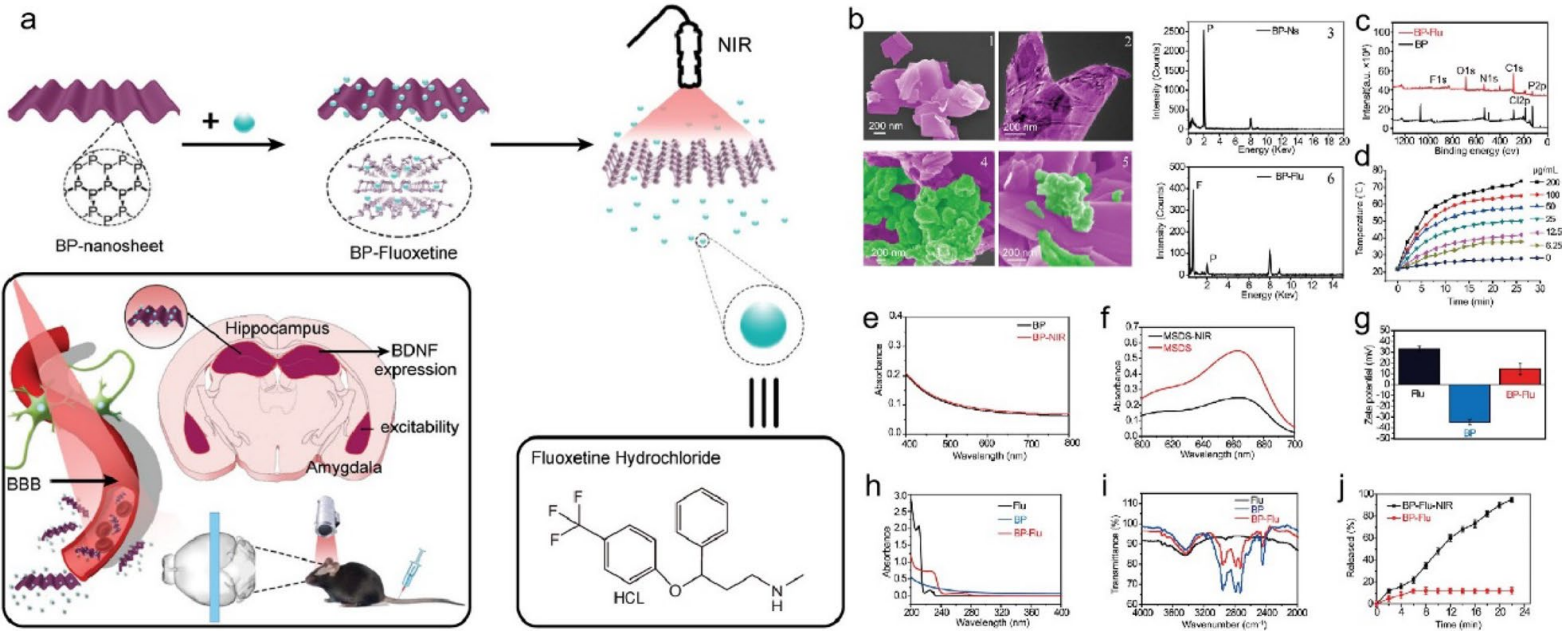
**a** Schematic procedure for fabricating BP-based drug delivery system for synergistic photothermal/chemotherapy of depression. **b** SEM, TEM, and EDS of BP nanosheets (b1, b2, b3) and BP-Fluoxetine (b4, b5, b6). Green colour represents Fluoxetine. **c** XPS survey spectra of BP nanosheets before and after Fluoxetine capturing. **d** The photothermal effect of BP nanosheets. **e**, **f** The UV–vis spectra of methylene blue (MSDS) and BP nanosheets before and after NIR irradiation. **g** Surface zeta analysis. **h** UV–vis absorption spectra of fluoxetine, BP, and BP-Fluoxetine (20 µg mL − 1). **i** FTIR spectra. **j** near-infrared defined fluoxetine releasing behaviour. Adapted with permission from Jin et al*.* [128]

Enhancing the drug solubility and increasing stimulus-responsive release have been shown to effectively improve passive and active transport, respectively [125]. For example, Gou et al*.* developed carboxyl-functionalised mesoporous silica NPs (MSN-COOH) using a silicon coupling agent (hyd-TESPSA), which enhanced drug loading and solubility for poorly water-soluble non-steroidal antiinflammatory drugs (NSAIDs) like nimesulide and indomethacin [126]. The MSN-COOH improved drug bioavailability and efficacy by converting the drugs to an amorphous state and enabling pH-responsive release, thereby enhancing passive diffusion and active targeting to specific tissues, which reduces side effects. In addition to pH-responsive formulations, temperature, light, magnetic, ultrasound, and electrical-responsive drug delivery systems are also being increasingly developed [127]. An example of a light-responsive system is the use of black phosphorus (BP) nanosheets for targeted drug delivery. Jin et al*.* demonstrated that BP nanosheets, which possess excellent biodegradability and biocompatibility, can effectively load the antidepressant fluoxetine (Flu) through electrostatic interactions [128]. Upon exposure to near-infrared (NIR) light, these BP-Flu composites facilitated a rapid and controlled release of Flu, significantly reducing treatment duration and minimising side effects associated with the free drug (Fig. 13).

In summary, nanomaterial drug delivery systems can provide targeted drug delivery, enhance therapeutic efficacy, improve the solubility and bioavailability of challenging drugs, and reduce toxicity. By integrating both active and passive targeting mechanisms, these systems achieve greater precision and improved biological safety. Additionally, they allow for precise drug release in diseased areas through internal responses to physiological changes and external responses to stimuli like pH and light.

##### Penetrating biological barriers

In addition to strategies for precise drug release to targeted tissues, effectively penetrating various biological barriers is crucial for maximising the efficacy of nanoformulations. These barriers include distribution barriers, microenvironmental barriers, and cellular and intracellular barriers [129].

**Fig. 14:**
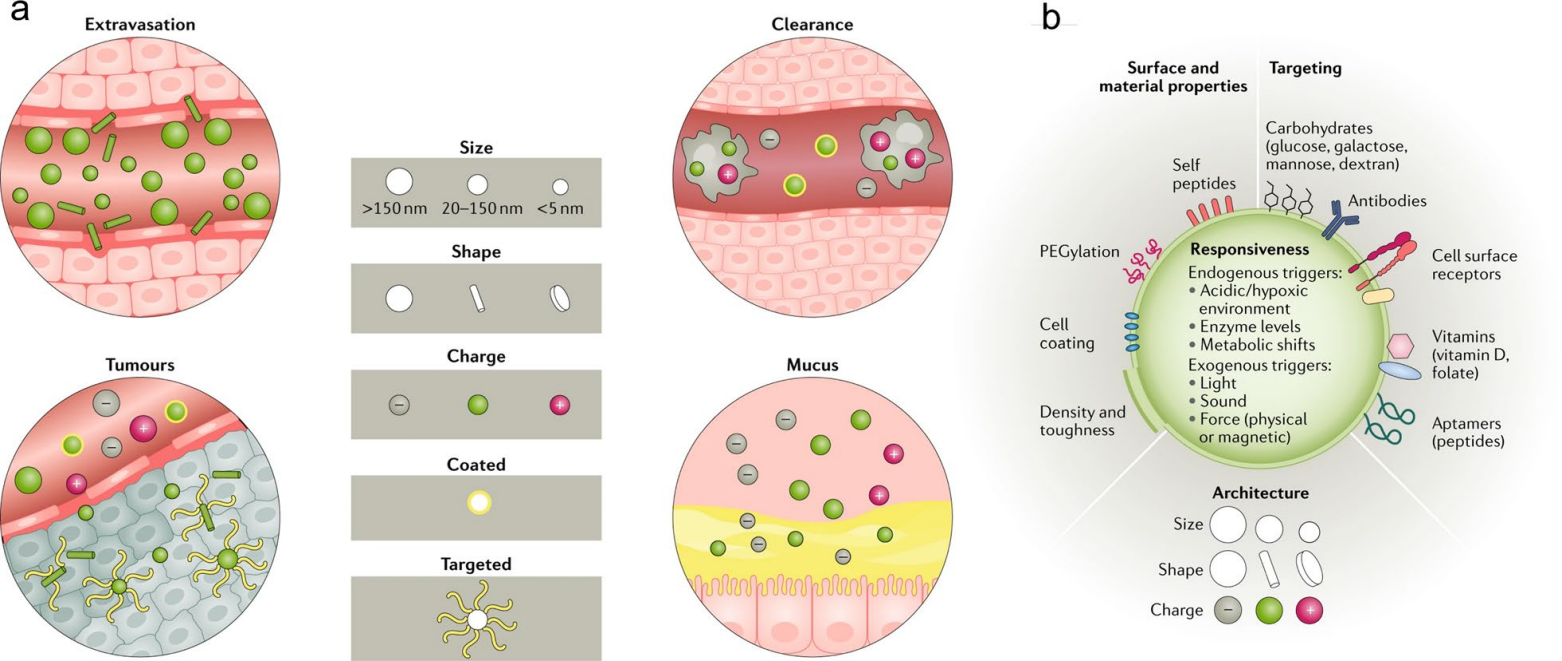
**a** Factors such as size, shape, charge, and surface coating influence the behaviour of NPs in circulation and their interaction with local barriers. Spherical and larger NPs marginate more easily, while rod-shaped NPs extravasate better. Uncoated or positively charged NPs are cleared faster by macrophages. Rod-shaped, neutral, and targeted NPs penetrate tumours more effectively, whereas positively charged, smaller, and coated NPs traverse mucosal barriers more readily. **b** The surface properties, material composition, architecture, targeting moieties, and responsiveness of NPs can be modified to suit specific applications, allowing for a wide range of tailored nanoparticle designs. Adapted with permission from Langer et al*.* [129]

First, optimising the route of administration can significantly influence nanomaterials distribution. For example, intravenously injected polymeric NPs, such as PLGA NPs, tend to accumulate in the liver and spleen, while subcutaneous or lymphatic administration directs them more effectively to local lymph nodes [130]. Inhalation can deliver drugs directly to the lungs, bypassing systemic circulation and avoiding first-pass metabolism in the liver [131]. For systemic delivery, modifying the NP surface, such as with PEGylation, can prolong circulation time and enhance drug exposure. Adjusting NP size is also critical (Fig. 14a: NPs smaller than 10 nm are quickly cleared by the kidneys, whereas those larger than 200 nm can trigger immune responses unless specifically engineered [132].

After reaching the target site, nanomaterial drug delivery systems encounter diverse microenvironments distinct from the circulatory system, such as acidic and enzyme-rich conditions in the gastrointestinal tract, low pH and high permeability in tumour tissues, and pH fluctuations in wound healing [133–135]. To overcome these challenges, NPs can be modified to respond to specific microenvironmental conditions. For example, pH-sensitive polymer coatings can enhance drug release in acidic environments [136], enzyme-resistant shells can protect NPs from degradation in the digestive tract [137], and penetration enhancers can improve NP delivery in tumour tissues [138]. Dynamic response modifications allow NPs to adapt to changing conditions, further enhancing therapeutic outcomes.

Modifying nano drug carriers can help them overcome cellular and intracellular barriers, ensuring effective drug delivery (Fig. 14b. Once inside the cell, NPs are often trapped in vesicles or endosomes, where the environment becomes increasingly acidic over time [139]. To promote drug escape from the endosome, some NPs are designed to respond to acidic or reductive environments, such as through the proton sponge effect or cleavable linkers, helping them disrupt the endosomal membrane and release the drug [140–142]. Additionally, complex-shaped NPs, such as nanostars, have shown promise in improving endosomal escape [143]. Once in the cytoplasm, drugs may need to traverse further intracellular membranes to reach specific organelles like the nucleus or mitochondria. pH-responsive nanoparticle systems are particularly useful in targeting these regions, especially for applications in gene editing or cancer immunotherapy [144, 145], as discussed further in the following sections.

#### Gene delivery

Expanding on drug delivery applications, these innovative nanoplatforms are now making significant strides in delivering gene therapeutics. Gene therapy is an experimental technique that exerts its therapeutic effects by introducing nucleic acids (DNA or RNA) into patient cells, enabling the expression of new genes or regulating the expression of target genes by correcting, disrupting or replacing them to prevent or treat a wide range of diseases [146, 147]. However, the effective in vivo delivery of nucleic acids into cells remains a significant challenge due to their low in vivo stability and susceptibility to rapid clearance from the bloodstream affecting their cellular uptake. Furthermore, nucleic acids have limited permeability through cell membranes owing to their electronegativity and large molecular size, posing a challenge to their effective cellular entry [148, 149].

Gene therapy can be stratified into DNA and mRNA-based therapy, with each encountering distinct challenges. In DNA-based therapies effective gene delivery involves overcoming extracellular barriers including enzymatic degradation and immune clearance, as well as intracellular barriers endosomal entrapment and nuclear transport across the nuclear envelope, a necessary step in DNA delivery and successful transgene expression [150]. Similarly, mRNA-based therapies must maintain the stability of the mRNA amid nucleases within the extracellular environment and avoid clearance [151, 152]. Unlike DNA therapies, in mRNA-based therapies, translation occurs in the cytosol rather than the nucleus, making endosomal escape a key intracellular barrier to conquer [154]. These challenges necessitate the development of vectors that can overcome extracellular and intracellular barriers to deliver genetic materials into target cells. Traditional vectors such as viral vectors are limited by obstacles such as immunogenicity, rapid clearance, limited genome packaging capacity, and potential carcinogenicity [149].

**Fig. 15:**
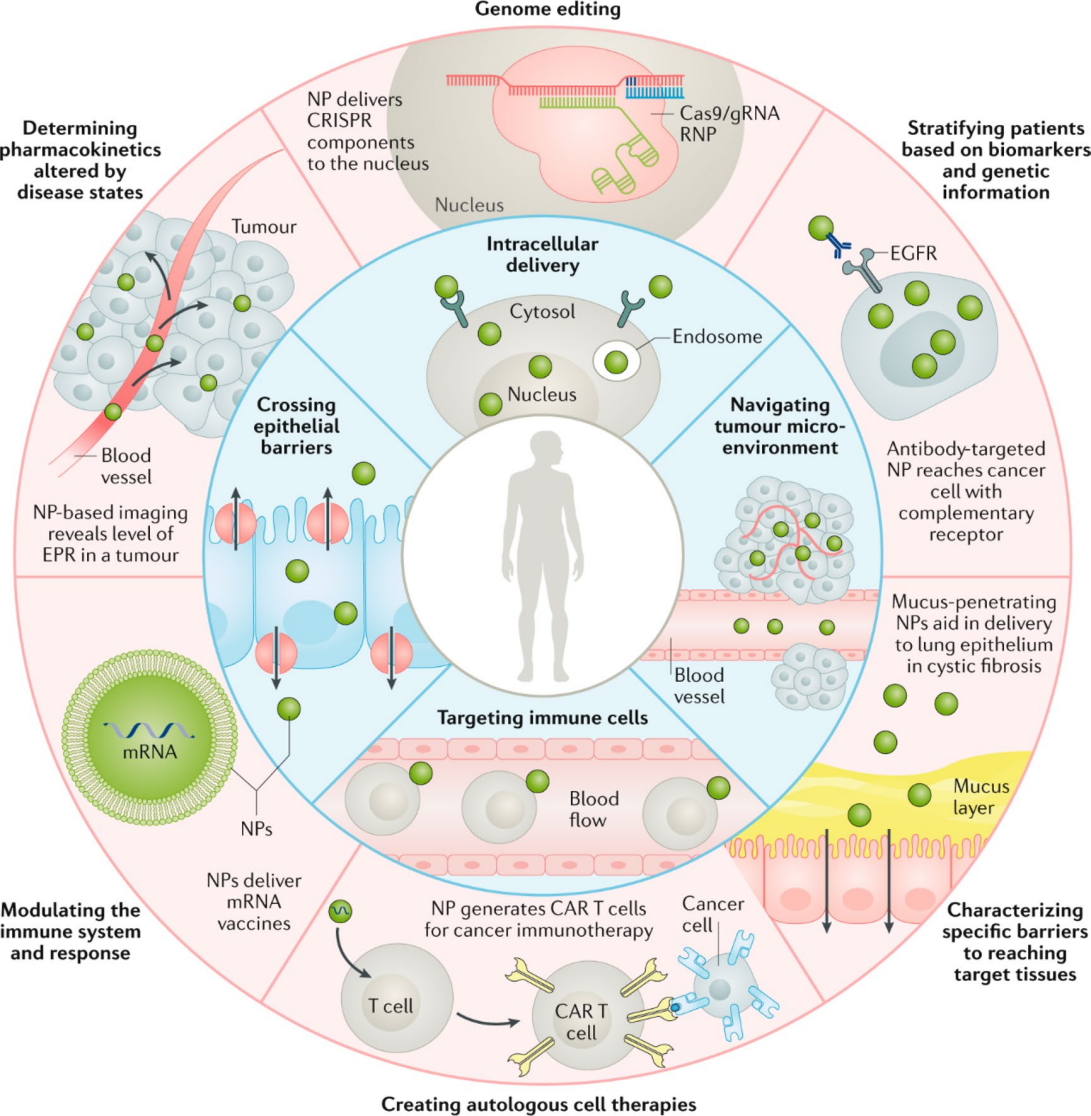
Schematic overview highlighting biological barriers that NPs can overcome (inner ring) and potential precision medicine applications that could benefit from NPs (outer ring). Intelligent NPs designs can improve the delivery of precision medicines, such as gene therapies, and accelerate their clinical translation. Abbreviations: CAR, chimeric antigen receptor; EGFR, epidermal growth factor receptor; EPR, enhanced permeation and retention; gRNA, guide RNA; RNP, ribonucleoprotein. Adapted with permission from Langer et al*.* [129]

Therefore, the ideal vector should protect genetic material, avoid clearance, enable cellular uptake, promote early endosomal escape, and be biocompatible, biodegradable, non-immunogenic and non-toxic to host cells [149]. Thus, efforts have shifted towards non-viral vectors, such as NPs, which have emerged as powerful tools to overcome the challenges associated with viral vectors for gene delivery by enabling safe and efficient targeting. These NPs boast a high drug-loading capacity, low mutagenicity and the ability to overcome biological barriers without provoking immune responses (Fig. 15) [149, 153].

Furthermore, NPs are hailed for their small size, high surface-to-volume ratio, and tunable properties that allow for selective targeting. Moreover, their ability to encapsulate genetic materials protects them from enzymatic digestion and facilitates efficient cellular uptake making them ideal vectors for gene delivery [155]. This protective capacity is exemplified with lipid NPs which have gained particular attention, especially with the recent success of the mRNA COVID-19 vaccines like BNT162b2 (Comirnaty^®^ by BioNTech and Pfizer) which demonstrated 95.0% effectiveness in preventing COVID-19 [156]. These lipid NPs protect the mRNA encoding the SARS-CoV-2 spike protein by encapsulating it into the core, ensuring its safe delivery into target cells where it can be translated and trigger an immune response [157].

**Fig. 16:**
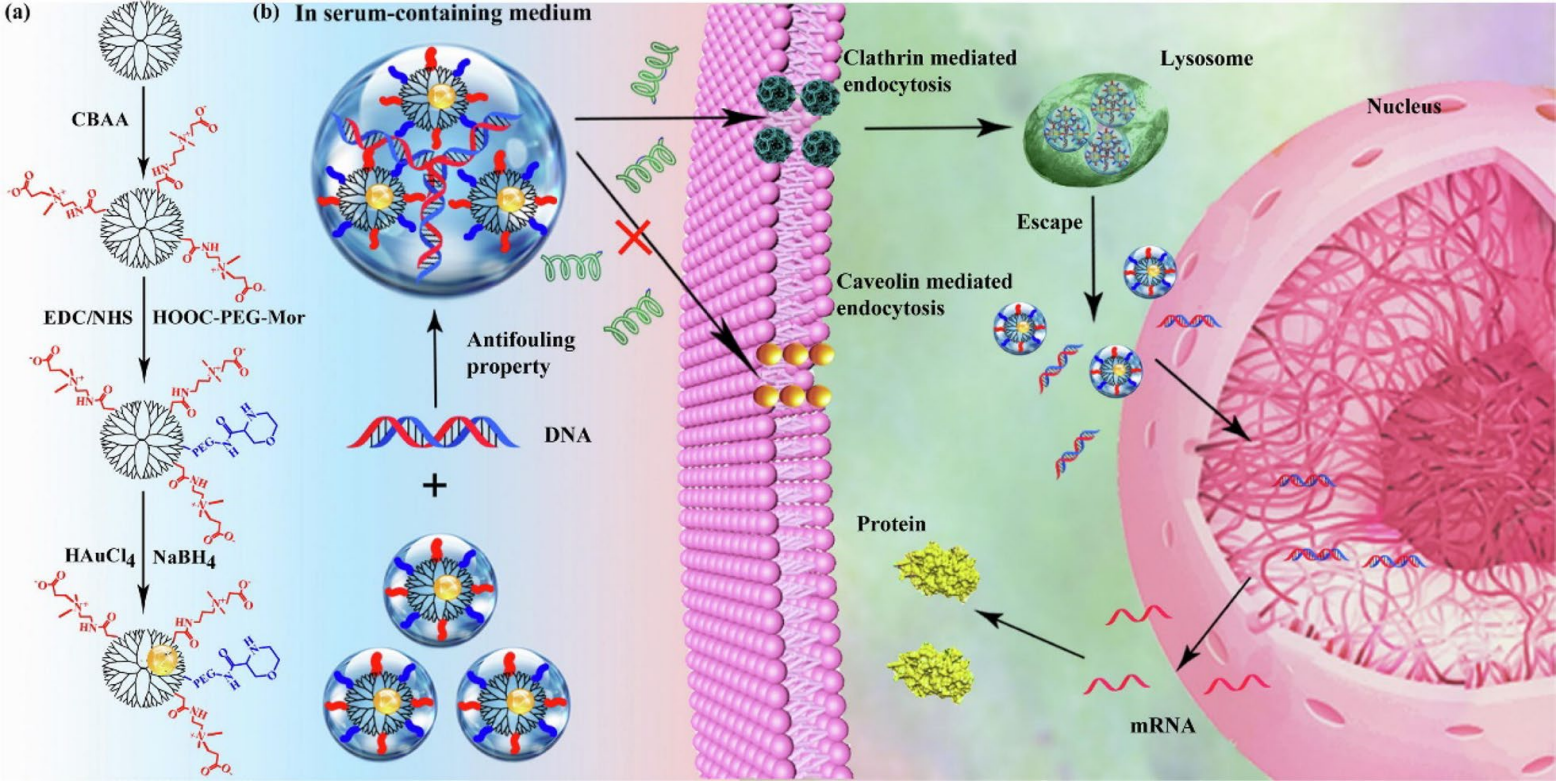
Overview of the synthesis procedure of Au DENPs (**a**) for gene delivery applications. **b** Notes for abbreviation: G5, generation 5; CBAA, carboxybetaine acrylamide; PEG-Mor, polyethylene glycol-morpholine; DENPs, dendrimer-entrapped NPs; EDC, 1-ethyl-3-(3-dimethylaminopropyl) carbodiimide hydrochloride; NHS, *N*-hydroxysuccinimide; and COOH-PEG-Mor, PEG-Mor with the other end of the carboxyl group. Adapted with permission from Xiong et al*.* [158]

In addition to safeguarding genetic cargo, NPs must also facilitate cellular uptake and gene transfection, a process that can be hindered by the formation of a protein corona by serum proteins, leading to the physical destabilisation and agglomeration of NPs. Xiong et al*.* developed zwitterion CBAA-modified gold dendrimer-entrapped NPs (Au DENPs) for serum-enhanced gene delivery to address this. Remarkably, the zwitterion coating conferred the NPs with antifouling properties, resisting serum protein adsorption and increasing gene delivery efficiency between 1.4 to 1.7-fold in serum-containing media compared with serum-free medium (Fig. 16). Consequently, NPs endowed with anti-fouling properties effectively enhanced gene delivery by preventing serum adsorption, suggesting their potential to circumvent immune clearance [158, 159].

**Fig. 17:**
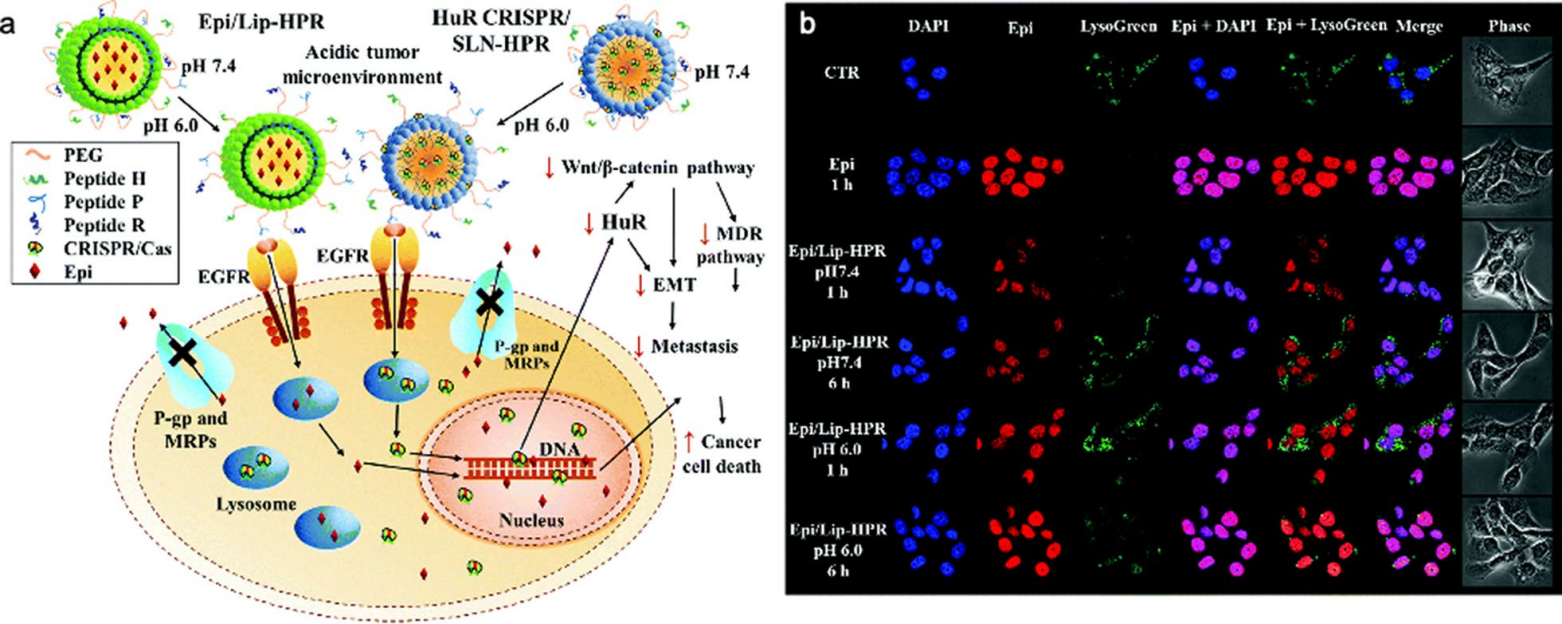
**a** Schematic illustration of how the HuR CRISPR/SLN-HPR and Epi/Lip-HPR NPs adapt in an acidic tumour environment due to the conformational changes of the H-peptide. The entry of NPs into SAS cells occurs via exposed P and R peptides. The CRISPR/Cas9 system knocked out the HuR gene, modulating several cancer pathways, while epirubicin accumulated in the nucleus, leading to cancer cell death. **b** pH-responsive localisation of Epi/Lip-HPR in SAS cells at pH 7.4 and 6.0, observed by CLSM. DAPI stains the nucleus; LysoTracker Green stains lysosomes. Adapted with permission from Wang et al*.* [161]

As previously discussed, DNA-based therapies require the DNA cargo to enter the nucleus for efficient transgene expression [150]. However, once inside the cell, NPs delivering DNA encounter several significant obstacles involving escaping from endosomal compartments before lysosomal trafficking and nucleus translocation which involves crossing the nuclear envelope, a formidable barrier, for transcription. To overcome this, NPs have been designed with nuclear targeting capabilities and nuclear localisation signals to ensure gene delivery across the nuclear envelope [160]. Wang et al*.* formulated pH-sensitive NPs delivering the CRISPR/Cas9 system and epirubicin to the nucleus in cancer cells (Fig. 17). These stimuli-responsive NPs exploit EGFR-targeting peptides and nucleus-localising sequences that enable endosomal escape and direct nuclear localisation under acidic tumour conditions [161]. By exposing targeting peptides at a lower pH, cellular uptake and transfection efficiency were enhanced. This demonstrates the potential of NPs to successfully target and traverse the nuclear envelope resulting in efficient gene delivery.

In conclusion, NPs are proving to be sophisticated vectors for overcoming key challenges in gene delivery. The development of these advanced nanomaterials has significantly enhanced the potential of gene delivery systems, offering solutions to the limitations of traditional viral vectors. The continued innovation in nanomaterial design and functionalisation is anticipated to result in more effective and safer gene delivery systems, ultimately enhancing therapeutic outcomes and broadening the scope of their applications.

#### Cancer therapy

Cancer remains a prominent cause of mortality globally, with conventional approaches identified for tumour-specific treatment: surgical resection, chemotherapy, and radiation therapy [162]. However, despite these treatment options, cancer patients often face short survival expectation and poor life quality.

**Fig. 18:**
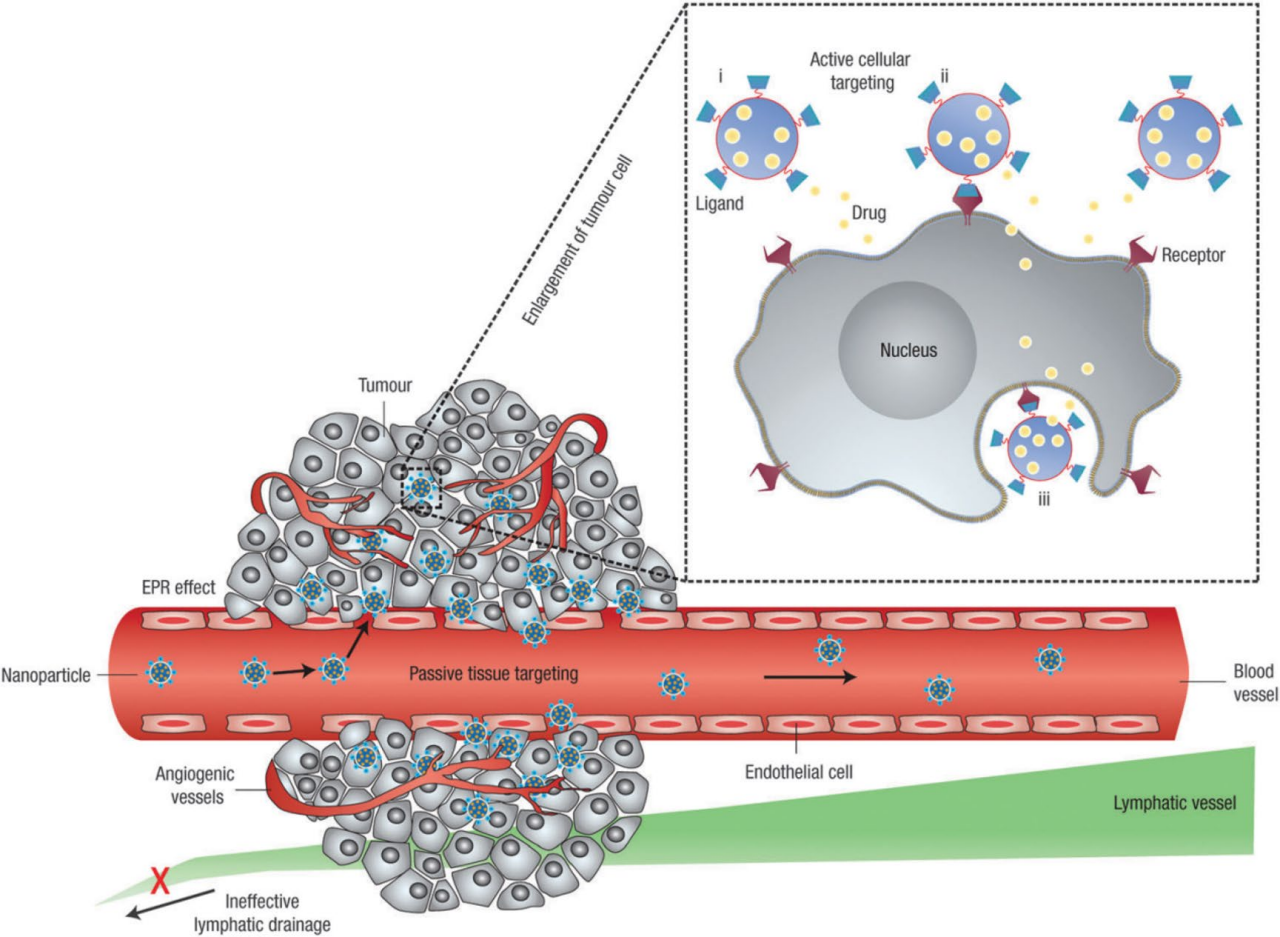
Passive and active tumour targeting. NPs achieve passive targeting by extravasating through the tumour’s permeable vasculature and poor lymphatic drainage (EPR effect). Active targeting is accomplished by functionalising NPs with ligands that bind specifically to target cells. Once targeted, NPs can (i) release their payload near target cells, (ii) adhere to the cell membrane as an extracellular sustained-release depot, or (iii) be internalised into the cell for direct drug delivery. Adapted with permission from Peer et al*.* [167]

The rapid development of nanotechnology has introduced complementary and alternative strategies for cancer treatment by using both passive targeting, due to the small size of NPs, and active targeting, achieved through specific modifications to the NPs, thereby offering greater precision in therapy [163]. As mentioned in Sect. “Drug delivery”, passive targeting of NPs takes advantage of the EPR effect, which occurs due to the abnormal structure of tumour blood vessels. These vessels have looser walls and inadequate lymphatic drainage, allowing macromolecules or NPs to penetrate and accumulate more easily within tumour tissues [164]. Active targeting, in contrast, involves the surface modification of NPs with specific ligands such as antibodies, peptides, or carbohydrates that bind selectively to receptors on tumour cells (Fig. 18). This enhances the precision of drug delivery, exemplified by the use of trastuzumab, an antibody targeting the HER2 receptor, or folic acid ligands targeting the folate receptor [165, 166].

Modified NPs frequently combine both passive and active targeting mechanisms to effectively direct them to tumour tissues, addressing the limitations of traditional cancer therapies, such as limited efficacy, toxicity, severe side effects, cancer recurrence, and drug resistance [168]. The following section explores the use of nanomaterials in surgical resection, radiation therapy, chemotherapy, and other novel therapies.

**Fig. 19:**
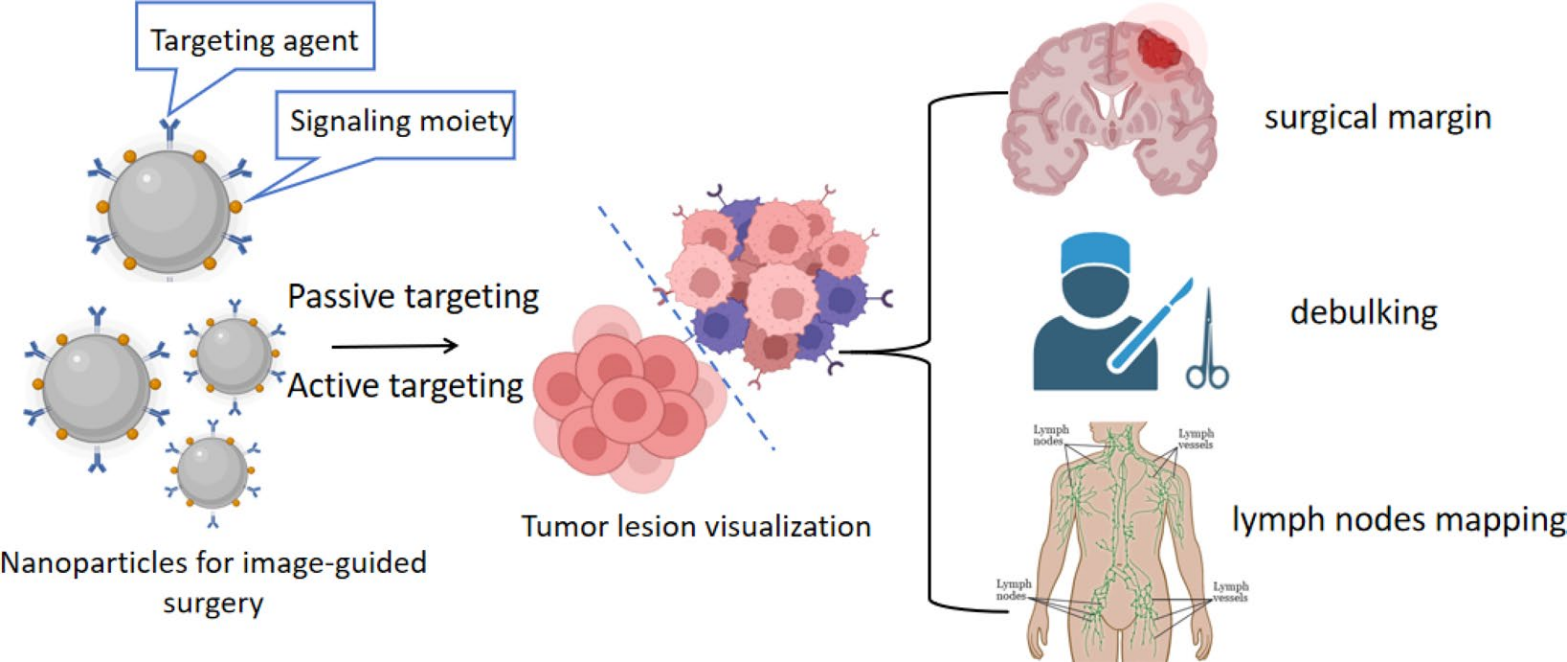
NPs development promotes improved precision in surgical tumour removal

Accurate detection of malignant and healthy tissues is crucial for the therapeutic efficacy of surgical resection. As described in Sect. “Medical diagnostics”, nanomaterials such as QDs, surface-enhanced Raman spectroscopy (SERS) NPs, luminescent NPs and dye nanoformulations, are able to act as contrast agents for image-guided surgery [169]. These modified NPs differentiate between tumours and nearby normal tissues through active targeting and passive targeting effects, thus helping surgeons to identify surgical margins and local metastases with high resolution and sensitivity, leading to improved therapeutic outcomes (Fig. 19).

In addition, many nanomaterials have been used to construct fast and efficient hemostatic measures that significantly improve safety during surgical treatments [170]. After surgery, wound healing involves three primary stages to stop bleeding. First, the damaged blood vessels constrict to reduce blood flow. Next, platelets aggregate to form an initial platelet plug that seals the wound. Finally, the coagulation cascade activates, producing fibrin that combines with the platelet plug to form a stable clot, effectively preventing further blood loss [171]. NPs have unique advantages in promoting hemostasis by enhancing platelet activation and aggregation, similar to platelet-activating factors. For example, Liang et al*.* synthesised zeolitic imidazolate framework (ZIF-8) nanoparticle-enhanced cryogels that provide rapid hemostasis during tumour resection surgery by promoting coagulation and stopping bleeding [172]. Additionally, these cryogels help prevent cancer recurrence after liver cancer surgery by releasing drugs in response to pH changes and generating reactive oxygen species under ultrasound to kill remaining cancer cells.

**Fig. 20:**
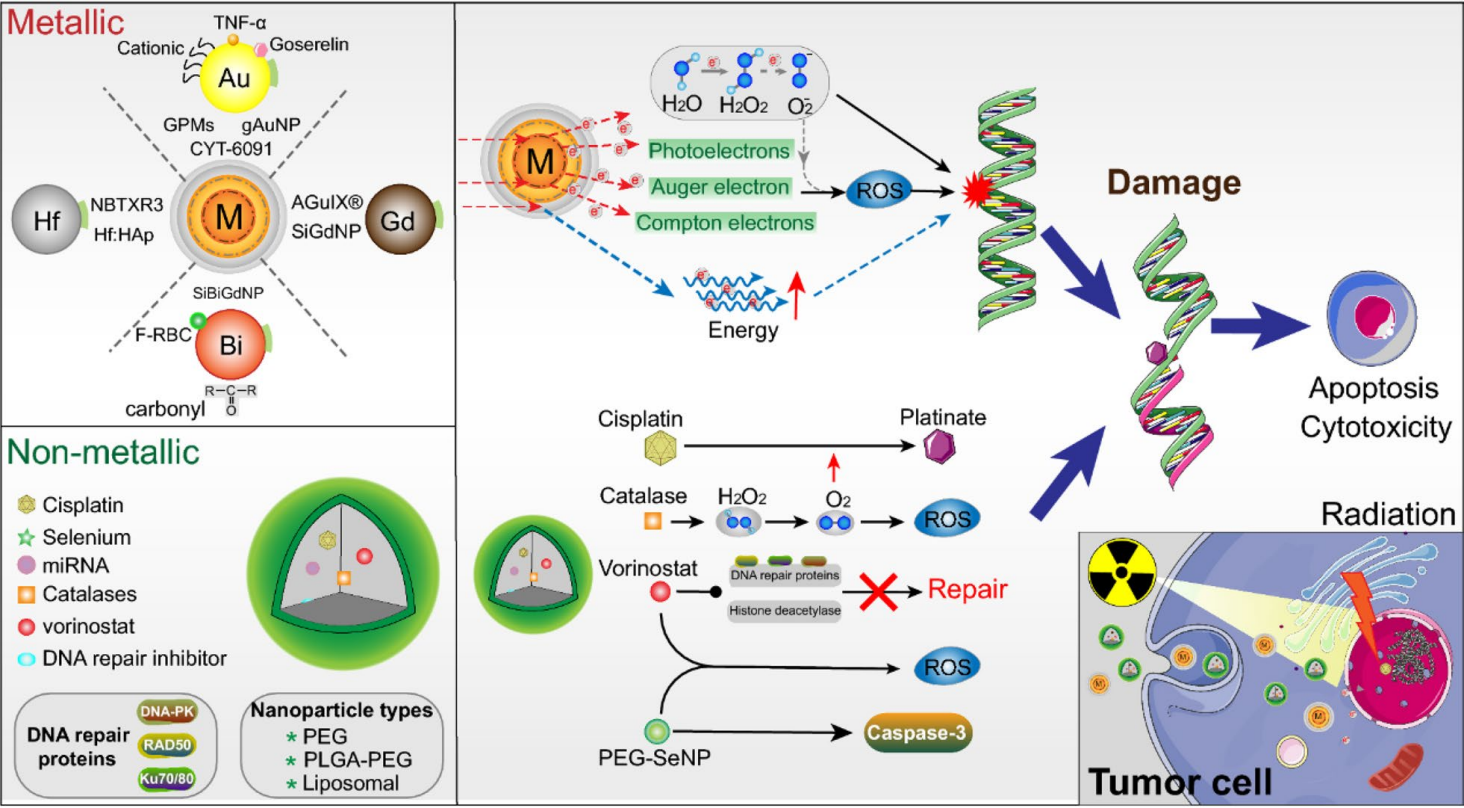
Schematic of NP functional mechanisms in radiotherapy. Combing ionising radiation (IR) with NPs can boost radiosensitisation, cell apoptosis and cytotoxicity. Upper: Metallic NPs (Au, Hf, Gd and Bi) deposit the IR dose through interactions, such as electron secretion (Compton, Auger and photoelectric), ROS generation and energy amplification. Lower: Non-metallic NPs-encapsulated combined with radiotherapy further induced DNA damage and prevented rapid DNA repair, which will cause more cell apoptosis. Adapted with permission from Jin et al*.* [175]

In radiation therapy, therapeutic effects often necessitate higher doses of irradiation, as tumour cells exhibit low radiation absorption. However, this can result in significant damage to surrounding normal tissues [173]. Therefore, the development of radiosensitisers and methods to protect normal tissues is a major strategy to increase the sensitivity of tumours to radiation therapy and to minimise side effects. Nanomaterials that incorporate high atomic number elements, including Au-, gadolinium (Gd)-, bismuth (Bi)-, hafnium (Hf)- and tungsten (W)-based nanomaterials can increase intracellular radiation deposition, making them potentially ideal sensitisers for radiotherapy (Fig. 20) [174].

Targeted modifications can further increase intratumoural enrichment to absorb radiation energy and promote the efficacy of radiation therapy [176]. Jyothi U et al*.* developed a novel nanoparticle system, R11-NU7441 NPs, which consists of PLGA NPs conjugated with the prostate cancer cell-penetrating peptide R11 and encapsulated with the potent radiosensitizer 8-dibenzothiophen-4-yl-2-morpholin-4-yl-chromen-4-one (NU7441). This system is designed for active targeting specifically to prostate cancer cells and provides a sustained release of NU7441, significantly enhancing the effectiveness of radiotherapy [177].

In addition to radiosensitisation, inorganic-based nanomaterials, exemplified by wide-ranging oxides of cerium (Ce), manganese (Mn), tantalum (Ta), and vanadium (V), play an important role in radioprotection due to their excellent enzyme-mimicking properties and their ability to scavenge ROS generated by radiotherapy in normal tissues, thus potentially reducing radioinflammation [178].

Conventional chemotherapeutic drugs typically lack specificity, causing potential damage to normal tissue cells alongside cancer cells during chemotherapy, resulting in severe adverse effects. Additionally, drug resistance and poor water solubility also hinder the effectiveness of chemotherapy drugs in clinical use [179]. As detailed in Sect. “Drug delivery”, NPs transport chemotherapeutic drugs to the tumour site by direct targeting through chemical modification or passive targeting of tumour cells through the EPR effect, thereby reducing systemic toxicity [180–182]. Moreover, nanocarriers can effectively address issues such as poor solubility, rapid metabolism, unstable absorption, insufficient permeability, and drug resistance associated with small molecule drugs [183].

**Fig. 21:**
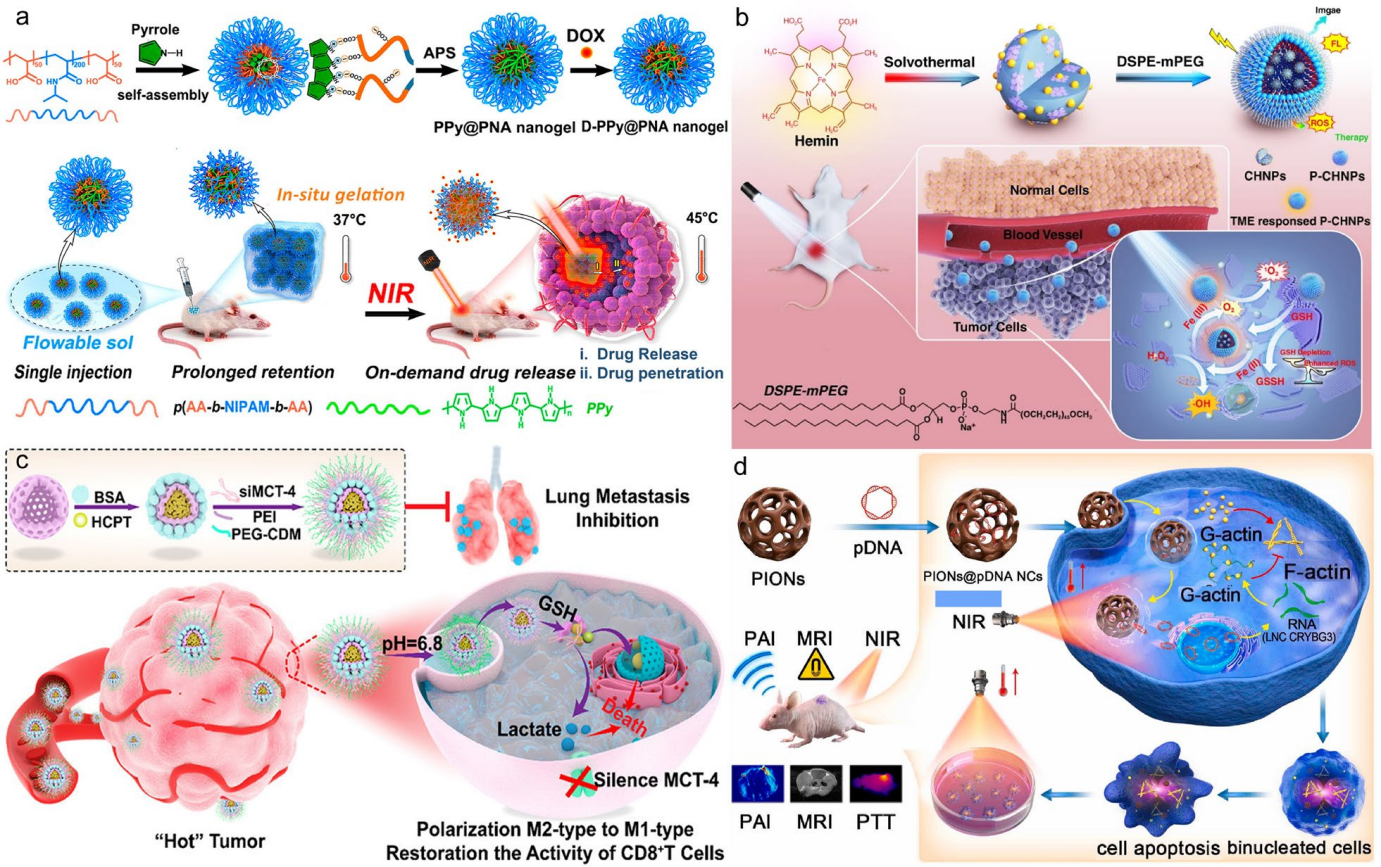
**a** The preparation of temperature-sensitive doxorubicin (DOX)-loaded complex polymeric nanogels of poly(acrylic acid-*b-N*-isopropylamide-*b*-acrylic acid/polypyrrole) (D-PPy@PNA nanogels) and in situ formation of D-PPy@PNA hydrogels for synergistic photothermo-chemotherapy. Adapted with permission from Geng et al*.* [184]. **b** Scheme of the synthesis approach of polymer encapsulated carbonised hemin NPs (P-CHNPs) and therapeutic mechanism of light amplified oxidative stress in tumour microenvironment by P-CHNPs for boosting photodynamic anticancer therapy. Adapted with permission from Lin et al*.* [185]. **c** Synthesis of HMONs@HCPT-BSA-PEI-CDM-PEG@siMCT-4, a surface-modified and redox (GSH)-responsive hollow mesoporous organosilica nanoplatform loaded with hydroxycamptothecin (HCPT) and monocarboxylate transporter 4-inhibiting siRNA (siMCT-4), administered for synergistic tumour chemo-immunotherapy. Adapted with permission from Li et al*.* [186]. **d** Schematic illustration of using porous iron oxide nanoagents (PIONs)-loaded with plasmid pcDNA3.1-LNC CRYBG3 nano-complexes (PIONs@pDNA NCs) as a photoporation nanoplatform for PTT and gene therapy. Adapted with permission from Huang et al*.* [187]

Expanding on traditional treatments, advancements in tumour understanding have spurred rapid development in new therapeutic approaches such as biotherapy (including cellular immunotherapy, gene therapy, etc.), photothermal therapy (PTT), and photodynamic therapy (PDT). Some examples are shown in Fig. 21.

These emerging methods significantly enhance comprehensive tumour treatment, improving patient prognosis and quality of life. PTT and PDT offer safety, non-invasiveness, and high efficiency [188]. Yet, traditional photosensitisers lack specificity and suffer from low water solubility, limiting their bioavailability. Nanocarriers boost photosensitiser accumulation in tumours, enhancing phototherapy effectiveness while minimising damage to healthy tissues [189].

While immunotherapy is esteemed, it encounters challenges like low patient responsiveness, limited tumour specificity, and immunosuppressive tumour microenvironments. Nanomaterial-based approaches, such as inducing immunogenic cell death (ICD), combining with immune checkpoint blockade therapy, cancer vaccines, adoptive immunotherapy, and immune microenvironment modulation, show promise in enhancing tumour immunotherapy efficacy [190].

Gene therapy introduces genes into cells to correct abnormalities and produce therapeutic proteins. It sidesteps the systemic toxicity and tolerance issues typical of chemotherapy [191]. The development of gene therapy relies heavily on nanotechnologies. As mentioned in Sect. “Gene delivery”, gold, polymer, and lipid NPs are amongst the main non-viral carriers for gene delivery, which can effectively deliver small molecules of nucleic acids, prevent their extracellular degradation by nuclease and improve drug distribution [192]. In conclusion, nanotechnology plays a vital role in numerous cancer treatments.

Nanotechnology is essential in many cancer treatments; however, NPs face challenges in tumour therapy due to individual heterogeneity among patients. The primary advantage of NPs in cancer therapy is their ability to specifically accumulate in tumour tissues through passive and active targeting mechanisms. Passive targeting largely relies on the EPR effect, but this effect is significantly influenced by various factors. Different tumour types, such as solid tumours like breast cancer and pancreatic cancer, may show a more pronounced EPR effect, while blood tumours or tumours with poor vascularsation may demonstrate a weaker EPR effect. The uneven angiogenesis of tumours, depending on their anatomical location and vascular characteristics, can also impact the EPR effect, particularly in tumours that are less vascularised or have poor vascular permeability. Additionally, the diverse microenvironments of tumours, characterised by different pressure states and compositions, affect the efficacy of the EPR effect and consequently the accumulation of NPs in tumour tissues. Furthermore, individual patient differences, such as variations in physiological state, hemodynamic characteristics, inflammation levels, and vascular permeability, contribute to the variability in the EPR effect [193–195].

To address the challenges posed by this individual heterogeneity and reduced targeting effectiveness, several strategies have been proposed. These include personalised treatment plans that assess each patient's tumour vascularisation and microenvironment characteristics to develop tailored NP delivery strategies, combination therapies that integrate both EPR and active targeting strategies to enhance the accumulation of NPs in tumours, and auxiliary methods such as using ultrasound or vascular permeability enhancers to boost the EPR effect in tumour tissues. These strategies aim to overcome the limitations of individual differences and optimise the therapeutic potential of NPs in cancer treatment.

#### Antimicrobial and antiviral applications

Overuse or misuse of conventional antibiotics in various sectors such as medical care, livestock farming, and agriculture has resulted in heightened antibiotic resistance, diminished efficacy of antibiotic therapies, and the rise of superbugs [196–198]. This resistance develops when bacteria adapt to survive antibiotics, often through mechanisms such as modifying target sites, producing enzymes that deactivate antibiotics, using efflux pumps to expel the drugs, reducing membrane permeability to prevent antibiotic entry, or forming protective biofilms that blocks antimicrobials penetration [199]. These adaptations not only complicate infection management but also contribute to higher mortality rates, prolonged hospital stays, and the need for more expensive and potentially toxic alternative therapies. Given the limitations of traditional antibiotics in overcoming these resistance mechanisms, there is an urgent need for innovative antimicrobial strategies.

Advances in nanotechnology present hopeful avenues for addressing these challenges. Nanomaterials enable deliberate engineering designs, including size control, surface modification, crystalloid change, and stimuli-responsive functionalisation, which offer unique interactions with bacterial cells compared to conventional antibiotics [200]. Such interactions frequently lead to distinctive killing mechanisms and exceptional antimicrobial attributes, including broad-spectrum activity, long-lasting effects, inhibition of biofilm formation and resistance-independent antimicrobial effects [201, 202]. By circumventing traditional pathways of resistance, nanotechnology-based solutions have the potential to revolutionise the fight against resistance, offering new hope for effective and sustainable antimicrobial therapies.

**Fig. 22:**
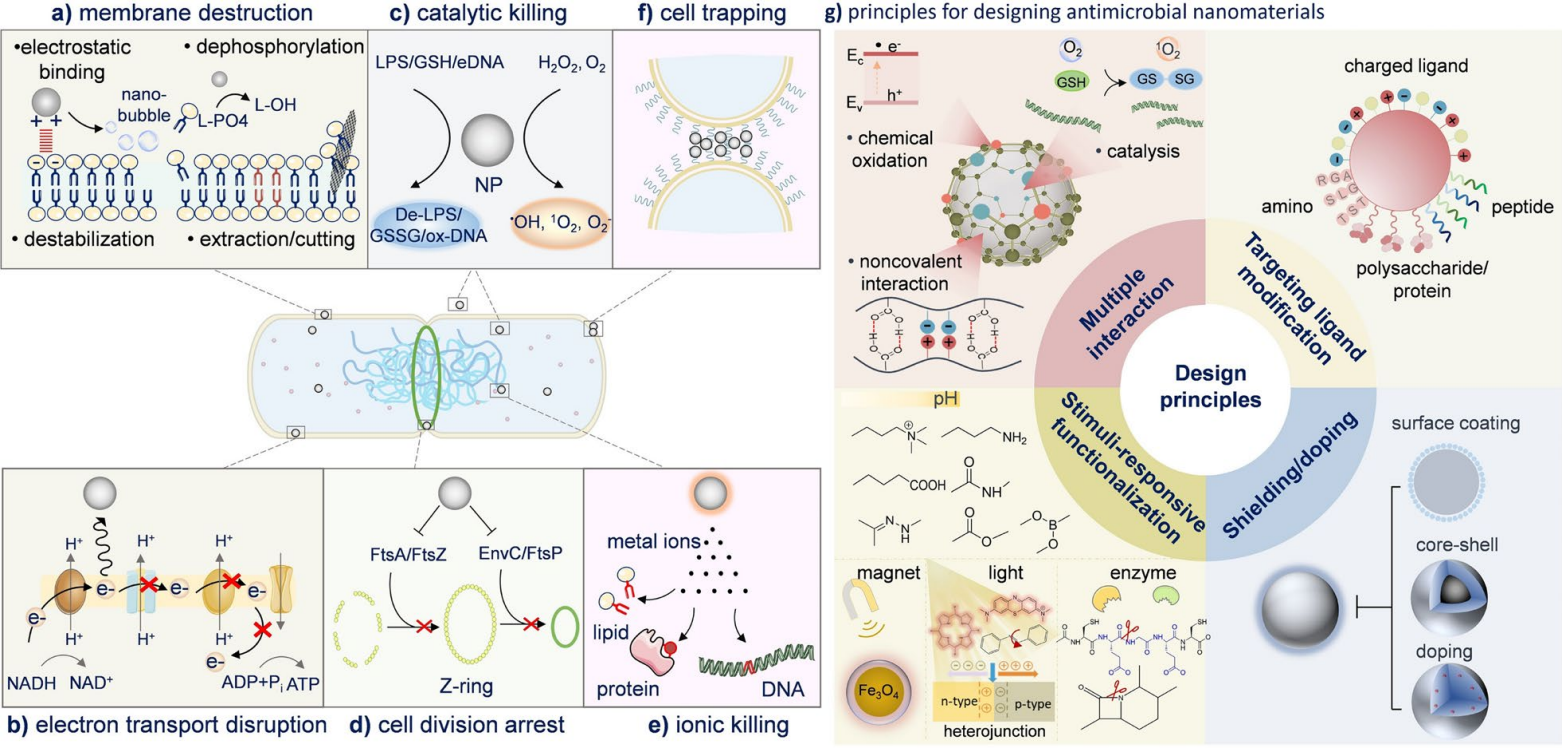
The antimicrobial mechanisms of nanomaterials operate through their Molecular Initiating Events (MIEs), which include the following: **a** Membrane destruction via physical or chemical interactions; **b** Disruption of the electron transport chain by metallic or semiconductor nanomaterials; **c** Catalytic killing by enzyme-mimicking actions that directly destroy critical biomolecules or induce ROS-mediated damage; **d** Arrest of cell division by blocking or degrading the Z ring, a cytoskeletal structure composed of the protein FtsZ that is essential for bacterial cell division; **e** Prolonged ionic killing by inactivating cellular enzymes, disrupting respiratory processes, and increasing intracellular ROS levels. **f** Cell trapping through nanoparticle aggregation via noncovalent interactions. **g** Four proposed principles for designing antimicrobial nanomaterials: (i) diversifying nano-microbe interactions (e.g., structural destruction, catalysis, noncovalent binding); (ii) enabling stimuli-responsive functionalisation (e.g., pH, magnet, light, enzyme); (iii) grafting targeting ligands (e.g., charged ligands, peptides); and (iv) doping/shielding modifications to prevent metal ion release. Adapted with permission from Xie et al*.* [202]

Five classes of antimicrobial nanomaterials have been extensively researched and reported, encompassing metallic NPs (such as Ag, Cu, Au, ZnO, La_2_O_3_, CeO_2_, V_2_O_5_, etc.), carbon-based nanomaterials (including graphene, GO, CNTs), borides (like boron nitride), nanosized polymers (such as polycarbonate), and nanocomposites (for instance, La_2_O_3_/Ag-GO) [202]. Based on the initial interactions between the materials and bacteria, Xie et al*.* proposed a summary of several antimicrobial mechanisms, including membrane destruction, disruption of the electron transport chain, catalytic killing, cell division arrest, cell trapping, and ionic killing (Fig. 22a–f) [202]. In addition, they proposed four design principles for fabricating antimicrobial nanomaterials to prevent the resistance evolution include: engineering nanocomposites to diversify nano-microbe interactions, enabling stimuli-responsive functionalisation for controllable bactericidal effects, grafting targeting ligands for precise bacterial killing, and incorporating doping/shielding to prevent triggering defense mechanism in bacterial cells due to the release of metal ions (Fig. 22g).

In addition to directly targeting bacteria, nanomaterials can combat antimicrobial resistance (AMR) by targeting bacterial biofilms through various mechanisms. Biofilms, protective layers formed by bacterial communities, are significant contributors to chronic infections and increased antibiotic resistance, particularly in the case of multi-drug resistant (MDR) infections, where bacteria are resistant to multiple antibiotics [203]. These structured communities of bacteria are encased in a self-produced extracellular polymeric substance (EPS) that protects them from external threats, including antibiotics, thereby complicating treatment efforts. Therefore, strategies that enhance the penetration of antimicrobials into biofilms, disrupt mature biofilms, and prevent biofilm formation are crucial for effectively combating the development of AMR and MDR infections [204].

By modulating the size, shape, and surface properties of NPs, it is possible to enhance their penetration into biofilms, thereby weakening the protective barrier provided by the biofilm [205]. For instance, while EPS can inhibit the penetration of silver ions, Ag NPs with diameters less than 20 nm can effectively penetrate biofilms of *Escherichia coli* and *Pseudomonas fluorescens* [206].

**Fig. 23:**
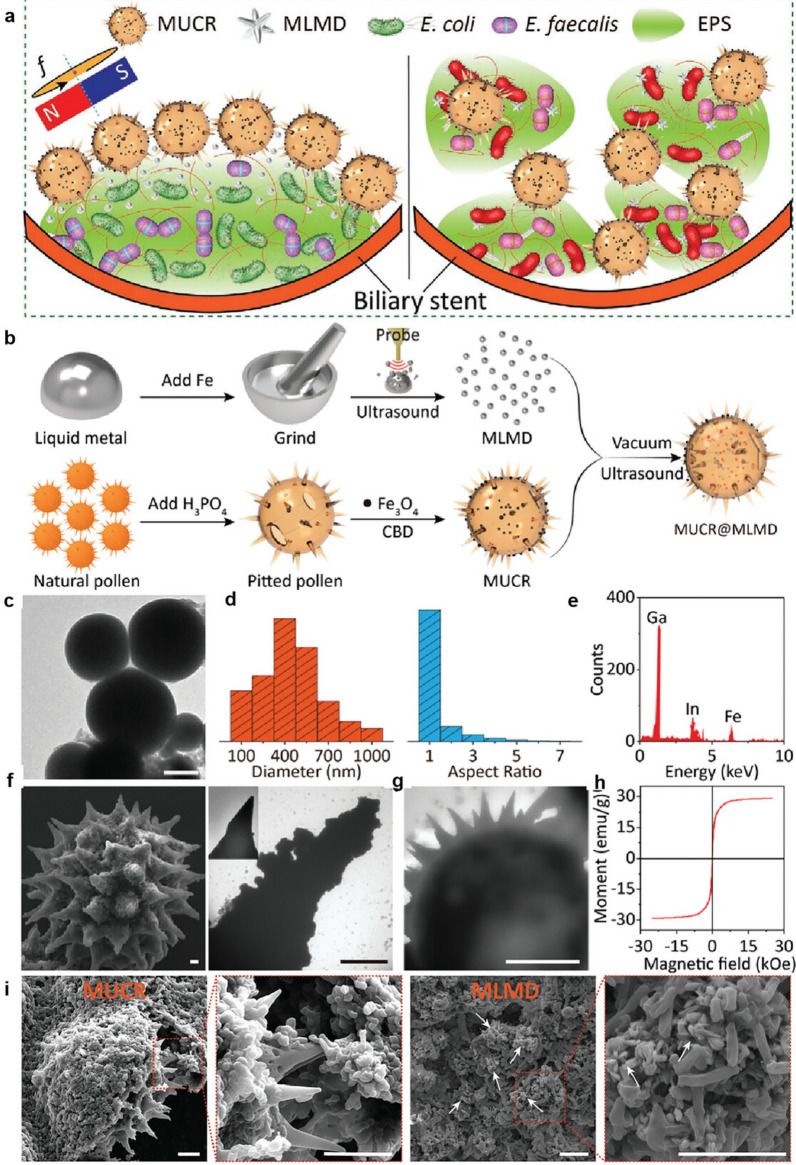
**a** Schematic of biofilm eradication on biliary stents using the MUCR@MLMD swarm. **b** Fabrication process of MUCR@MLMD. **c** TEM image of MLMD after sonication. **d** Size distribution histogram of MLMD. **e** EDX spectrum showing Ga, In, and Fe content in MLMD. **f** SEM image of MUCR post-chemical bath deposition and magnified TEM image of individual spines. **g** TEM image of MUCR@MLMD after vacuum loading and ultrasonic treatment. **h** Magnetic hysteresis loop of MUCR@MLMD. **i** Visualisation of biofilm eradication mechanism using microswarm; white arrows indicate deformed MLMDs. Reproduced with permission. Adapted with permission from Sun et al*.* [208]

Nanomaterials can also disrupt mature biofilms. NPs with unique shapes, such as nanosheets, nanoneedles, and sea urchin-shaped NPs, can mechanically disrupt the EPS of biofilms [207]. Magnetic nanomaterials have been shown to cause mechanical disruption of biofilms through the application of direct and alternating magnetic fields. For example, a magnetic urchin-like capsule robots (MUCRs) developed by Sun et al*.* loaded with magnetic liquid metal droplets (MLMDs) can change shape into spheroids and rods with sharp edges when triggered by an external magnetic field. These sharp edges, along with natural microspikes, can physically disrupt the protective function and mechanical stability of EPS (Fig. 23). Additionally, the applied mechanical force can break bacterial cells embedded within the biofilm, contributing to the overall destruction of the biofilm [208].

Beyond mechanical damage, electron transfer can also be used to disrupt biofilms. 2D transition metal disulfide (MoS_2_) nanosheets with nanoholes containing atomic vacancies can enhance electron transport interactions with biofilms, disrupting key components of mature biofilms such as proteins, polysaccharide intercellular adhesin (PIA), and extracellular DNA, while downregulating the expression of related genes [209]. Moreover, oxidative stress can not only damage bacterial cell membranes but also lead to the degradation of EPS, achieving a synergistic antibacterial and anti-biofilm effect. Various advanced nanomaterials have been reported to generate ROS and reactive nitrogen species (RNS) through different mechanisms, including photocatalysis, nanozyme catalysis, photodynamic therapy, sonodynamic therapy, and nitric oxide (NO) therapy [210]. For example, in a recent study supramolecular photosensitiser NPs were developed, capable of producing ROS under light irradiation to disrupt biofilms and release antibiotics, effectively combating MDR bacteria by breaking down the biofilm structure and enhancing drug penetration [211]. Another example is a polymeric NPs internally loaded with the photosensitiser chlorin e6 designed for root canal irrigation, which combines photodynamic therapy with NO release. This system enhances biofilm penetration and eradication by generating ROS and NO simultaneously, providing an effective strategy against endodontic bacterial infections, especially in the treatment of apical periodontitis [212].

**Fig. 24:**
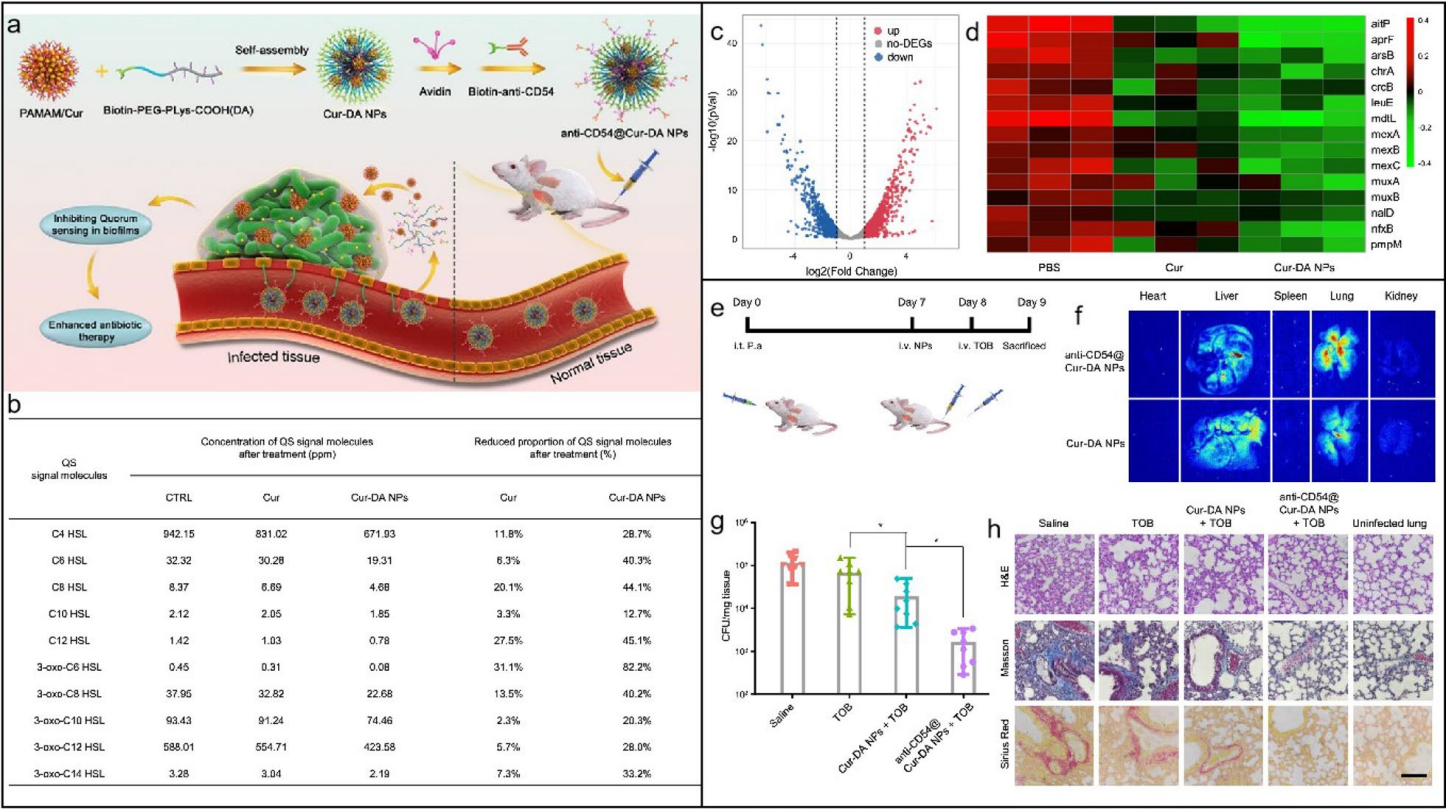
**a** Illustration of Anti-CD54@Cur-DA NPs delivery for inhibiting QS and enhancing antibiotic therapy against biofilm-associated infections. **b** HPLC analysis of QS signal molecules in *P. aeruginosa* treated with Cur-DA NPs or free curcumin (n = 3). **c** Volcano plot showing significantly upregulated (red) and downregulated (blue) genes after treatment with Cur-DA NPs. **d** Heat map of multidrug efflux-related genes in *P. aeruginosa* biofilms treated with Cur-DA NPs or free curcumin. **e** Timeline for chronic *P. aeruginosa* lung infection model and treatments. **f** Ex vivo fluorescent imaging of heart, liver, spleen, lungs, kidneys after 24 h treatment with anti-CD54@Cur-DA NPs or Cur-DA NPs. **g** Quantitative bacterial count in lungs after treatment with tobramycin (TOB, aminoglycoside antibiotics), Cur-DA NPs + TOB, or anti-CD54@Cur-DA NPs + TOB (n = 8). **h** The H&E, Masson's trichrome, and Sirius Red staining of lung slices post-treatment with TOB, Cur-DA NPs + TOB, or anti-CD54@Cur-DA NPs + TOB. Statistical analysis (t-test) showing significance: *p < 0.05, **p < 0.01, ***p < 0.001. Scale bar: 200 μm. Adapted with permission from Chen. et al*.* [216]

Certain nanomaterials do not combat bacteria and biofilm through direct interaction but instead inhibit biofilm formation and virulence expression by disrupting bacterial quorum sensing (QS) systems, the process by which bacteria communicate with each other [213–215]. This approach is expected to reduce the risk of developing resistance by avoiding selection pressure on bacteria. In addition, since QS systems are absent in mammals, therapies that block microbial QS reduce the risks of host toxicity. Chen et al*.* developed pH-sensitive curcumin-loaded nanoparticles (Cur-DA NPs and anti-CD54@Cur-DA NPs) to inhibit QS for treating biofilm-associated bacterial infections. These NPs enhance penetration and curcumin release in acidic biofilm environments, effectively inhibiting QS and improving antibiotic efficacy. The targeting modification with anti-CD54 boosts NPs accumulation at infection sites, significantly reducing bacterial load and inflammation in vivo, suggesting a promising antimicrobial strategy (Fig. 24).

In addition, nanomaterials have been used as delivery of antimicrobial agents, such as antibiotics and antimicrobial peptides [217]. Immobilisation of nanomaterials on implantable devices, wound dressings, bone cement, or dental materials can serve as antibacterial coating [218].

Viral infections represent a significant health challenge, posing risks to individuals worldwide. Nanomaterials have shown great advantages in the study of antiviral therapy. In general, antiviral nanomaterials can prevent viral infections by directly targeting virus particles, interfering with virus-host cell interaction, and suppressing viral proliferation [219]. For example, the sharp edged structure of GO can cause physical damage to viral structures [220]; Carbon dots synthesised from glycyrrhizic acid (Gly-CDs) are reported to directly inactivate porcine reproductive and respiratory syndrome virus (PRRSV), inhibit viral invasion and replication, and activate innate immunity [221]; Ag NPs were shown to inhibit the interaction between spikes over the viral membrane and target cell membrane receptors, thereby inhibiting human immunodeficiency virus (HIV-1) attachment [222]; Au NPs were reported to inhibit replication of foot-and-mouth disease virus (FMDV) [223]; MIP NPs that use whole viral particles as templates are able to specifically capture and rapidly remove viral particles from the environment, thereby preventing viral infections [224].

In addition, nanomaterials can be used as drug carriers to deliver antiviral drugs. For example, PLGA NPs, cationic nanogels, and chitosan nanospheres can deliver bictegravir, azidothymidine, and acyclovir, respectively, resulting in benefits such as sustained release, reduced cytotoxicity and dosing, increased site-specific delivery, and improved stability and solubility [225–227].

Nanomaterials offer promising solutions for both antibacterial and antiviral therapies through unique mechanisms and targeted delivery systems. Their ability to prevent resistance evolution, enhance drug efficacy, and reduce toxicity highlights their potential to revolutionise infectious disease treatment in healthcare applications.

### Future trends in nanotechnology for healthcare applications

Building on the previous discussion of nanotechnology's major applications in healthcare-medical diagnostics, tissue engineering, drug delivery, gene delivery, cancer therapy, and antimicrobial/antiviral applications-this section explores the future trends of nanotechnology in the field. As nanotechnology continues to revolutionise healthcare, it offers innovative solutions that promise to enhance the efficacy and personalisation of medical treatments. Key emerging trends such as personalised medicine, theranostics, and smart drug delivery systems are particularly noteworthy. These advancements not only represent cutting-edge technological progress but also have the potential to significantly impact the future of healthcare by enabling more precise, effective, and tailored medical interventions.

Personalised medicine aims to tailor treatments to the individual characteristics of each patient, making therapies more effective and reducing side effects. Nanotechnology plays a crucial role in this domain by enabling the development of nanoformulations that are specifically designed to interact with individual genetic profiles, thereby enhancing therapeutic outcomes. Jhawat et al*.* highlighted the integration of pharmacogenomics with nanotechnology, proposing that nanoformulations could lead to error-free and targeted therapeutic agent delivery, which is essential for personalised healthcare [228]. In addition, nanoinformatics, which combines nanotechnology with artificial intelligence, is becoming a powerful tool in personalised medicine. By analysing patient-specific data, nanoinformatics enables the design of better nanomaterials for personalised drug delivery, thus improving treatment precision [229].

**Fig. 25:**
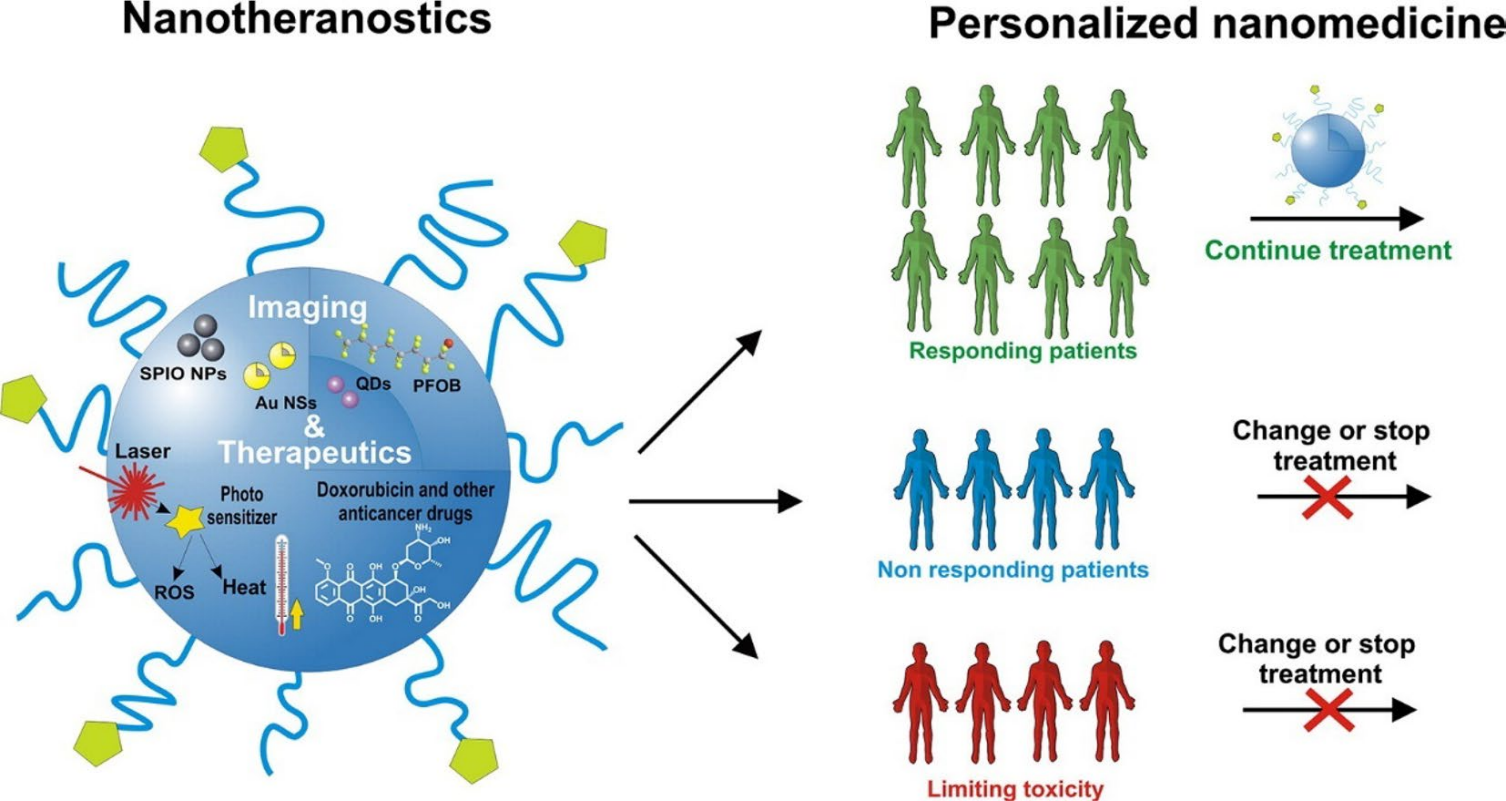
Schematic illustration of nanotheranostics and personalised medicine. Nanotheranostics integrates diagnostic and therapeutic capabilities into a single platform for precise drug delivery and real-time treatment monitoring. Personalised nanomedicine allows for treatment adjustments based on patient response, optimizing effectiveness and minimising toxicity. Adapted with permission from Mura. et al*.* [232]

Theranostics is an innovative approach in medicine that integrates therapeutic and diagnostic capabilities into a single platform, allowing for the simultaneous diagnosis, treatment, and monitoring of diseases. This dual functionality enhances the precision of medical interventions by utilising advanced nanomaterials and imaging techniques to deliver therapeutic agents directly to targeted sites while continuously monitoring treatment efficacy and disease progression [230]. The real-time feedback loop provided by theranostics enables clinicians to adjust treatment strategies based on individual patient responses, optimizing therapeutic outcomes. A notable advancement in this field is optical imaging-guided nanotheranostics, which utilises advanced imaging modalities such as MRI and CT to enhance the precision of cancer treatments. This approach not only allows for accurate visualisation of tumours but also improves the monitoring of therapeutic responses and enables real-time adjustments to treatment, thereby supporting personalised medicine (Fig. 25) [231].

Theranostics is particularly promising in cancer care, where real-time monitoring ensures precise drug delivery to tumour sites, minimising damage to healthy tissues and reducing systemic toxicity [233]. Furthermore, it facilitates tracking therapeutic efficacy at the molecular level, offering insights into the biological responses of tumours, which aids in early detection of resistance and supports adaptive, personalised treatment plans tailored to the cancer's evolving nature [234]. These personalised strategies have been shown to significantly enhance patient outcomes by enabling more targeted and effective therapies [233]. Beyond oncology, the principles of targeted delivery and real-time monitoring that define theranostics have the potential to transform the management of other conditions, including cardiovascular diseases, neurological disorders, and infectious diseases, leading to more precise, personalised, and efficient healthcare across various medical fields [235].

Smart drug delivery systems are designed to release therapeutic agents in a controlled manner, specifically targeting diseased cells while minimising side effects on healthy tissues. This precision is achieved through the use of stimuli-responsive nanomaterials that can react to environmental changes such as pH, temperature, or magnetic fields. For example, polymeric nanodroplets have emerged as versatile carriers for gas and drug delivery, showing great potential in treating conditions like hypoxia and cancer [236]. Some cutting-edge examples have been presented in the Sect. “Drug delivery” and “Gene delivery”. The integration of 3D printing with nanotechnology is also advancing smart drug delivery systems, allowing for the creation of complex, multifunctional medical products tailored to individual patient needs. This combination is expected to lead to significant advancements in personalised medicine, particularly in tissue engineering and regenerative medicine [237].

**Fig. 26:**
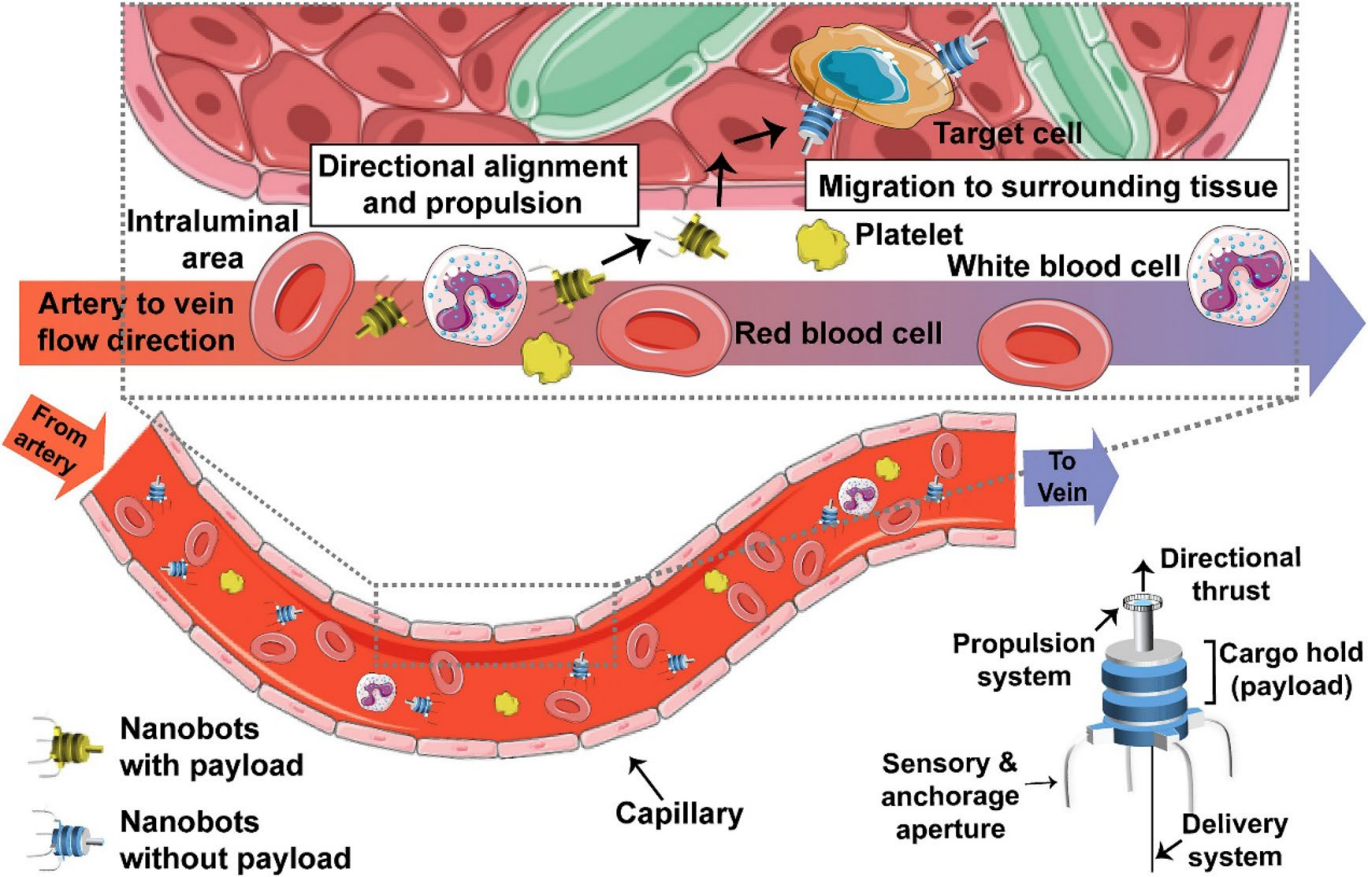
Schematic of a nanorobot for drug or cell delivery, highlighting its four key components: propulsion, payload cargo hold, sensory and anchorage system, and delivery mechanism, designed for precise and controlled operation within body fluids and tissues. Adapted with permission from Agrahari. et al*.* [238]

Nanorobots are at the forefront of nanotechnology's impact on healthcare. These tiny robots can navigate through the body, delivering drugs precisely where they are needed, and are expected to play a significant role in personalised and precision medicine (Fig. 26). However, there are still challenges to overcome, particularly regarding safety and the scalability of production for clinical use [238]. Another exciting development is the use of molecularly imprinted polymers in drug delivery. As discussed in Sect. “Drug delivery”, MIPs are synthetic polymers with the ability to recognise and bind specific molecules, making them ideal for targeted drug delivery systems that require high specificity and efficiency [122].

Nanotechnology is set to transform healthcare by enabling more personalised, precise, and effective treatments. The continued development of personalised medicine, theranostics, and smart drug delivery systems promises to significantly enhance patient outcomes, particularly in the treatment of complex diseases like cancer. As these technologies evolve, their integration with other emerging fields, such as artificial intelligence and 3D printing, will likely lead to even more groundbreaking innovations in healthcare.

To fully harness the advancements described in the above sections, however, it is essential to translate these technologies from the laboratory to clinical practice. This process involves conducting rigorous clinical trials, developing scalable manufacturing processes, and obtaining regulatory approval. The following section will examine the progress in transitioning nanotechnology-based innovations to clinical settings, with a particular focus on global regulatory approvals for healthcare products.

## Clinical translation and commercialisation of nanotechnology

Healthcare products are categorised into medicines (drugs) and medical devices, aligning with the regulatory frameworks of various countries. Therefore, nanotechnology and nanomaterials used in healthcare are necessarily regulated either as nanomedicines or as nanotechnology-based medical devices (NMDs) during clinical translation and commercialisation. Nanomedicines refer to medical applications that use nanomaterials to diagnose, treat, and prevent diseases. This category includes nanopharmaceuticals (i.e., drug formulations that enhance drug delivery and efficacy), nanoimaging agents (which improve the visualisation of biological processes for better diagnostics), and theranostics (which combine therapeutic and diagnostic functions into a single platform, thus allowing for simultaneous treatment and monitoring) [239]. NMDs, on the other hand, encompass a variety of medical tools and equipment that incorporate nanoscale properties to improve their functionality and compatibility with biological systems. These devices include: (i) diagnostic devices, which provide highly sensitive and specific disease detection; (ii) implantable devices, which integrate seamlessly with biological tissues; (iii) skin dressings, which offer superior wound healing properties; and (iv) surgical tools, which enhance precision and reduce invasiveness.

Before nanomedicines and NMDs can be applied clinically, they must undergo a rigorous approval process. This process involves preclinical studies and clinical trials to ensure their safety, efficacy, and quality. Regulatory bodies such as FDA, the European Medicines Agency (EMA), and China's National Medical Products Administration (NMPA) govern this process, requiring extensive testing and evaluation to meet stringent standards [240].

**Fig. 27:**
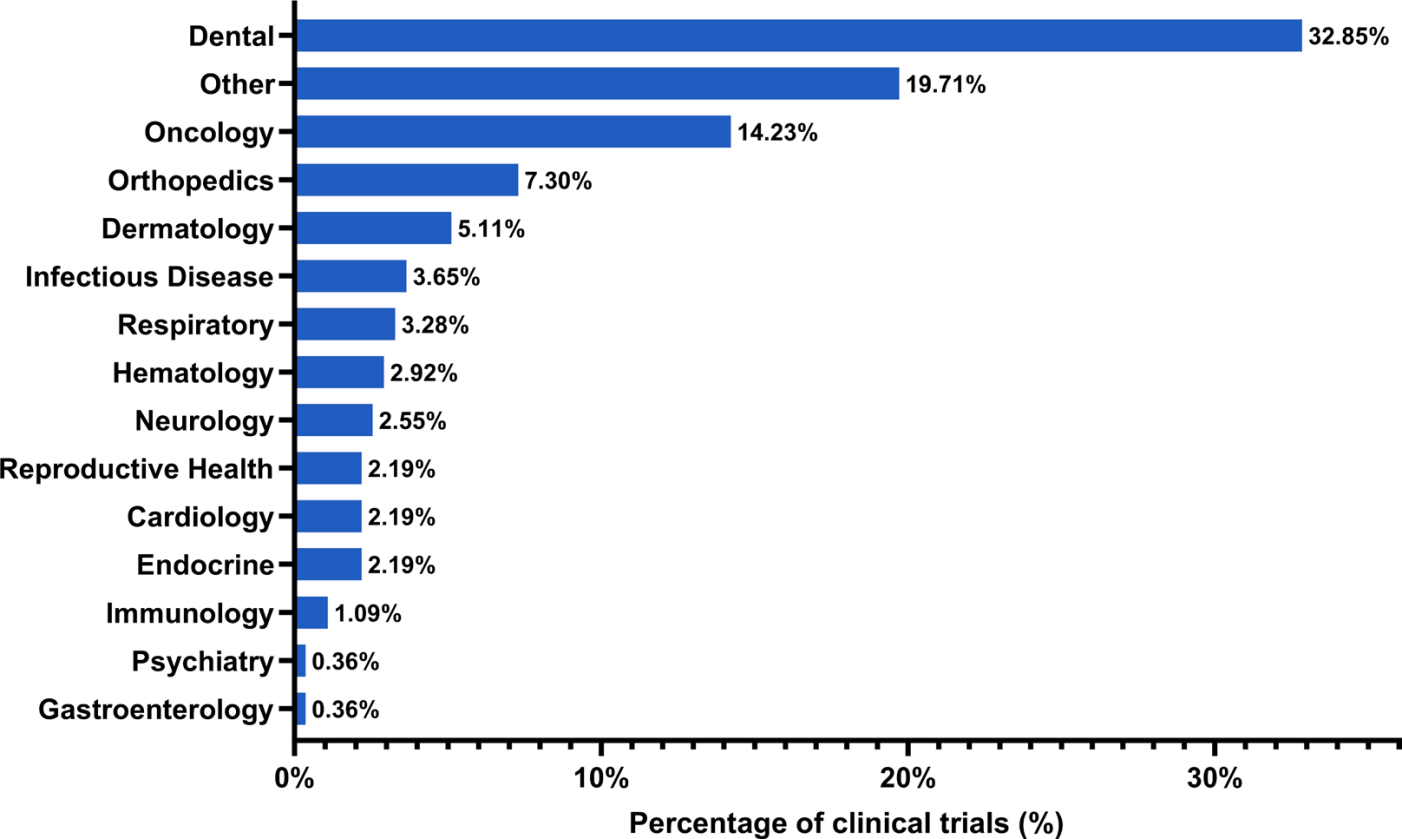
Distribution of clinical trials for nanotechnology-based healthcare products across different medical applications and specialties. Extracted from ClinicalTrials.gov database https://clinicaltrials.gov/ (keyword: nano)

The current landscape of clinical research on nanomedicines and NMDs can be evaluated using data from the ClinicalTrials.gov database. Managed by the National Library of Medicine (NLM), a division of the United States National Institutes of Health (NIH), ClinicalTrials.gov is one of the largest global registries, offering extensive information on clinical trials conducted worldwide [241]. As of August 2024, a search of this database using "nano" as a keyword in the intervention/treatment field identified 336 relevant clinical trials. Of these, 154 trials have been completed, while 20 have been suspended, withdrawn, or terminated. The conditions targeted in these 336 nano-related trials span a broad spectrum of health issues, diseases, and disorders, as illustrated in Fig. 27. This diversity of research areas highlights the extensive exploration of nanotechnology applications across various medical fields, with a notable concentration of studies focusing on dental, orthopedic, and cancer treatments.

Despite the challenges associated with transitioning from research and development (R&D) to market—such as high costs, complex manufacturing processes, and regulatory hurdles—several nanomedicines and NMDs have successfully reached the market [242]. These products are revolutionising healthcare by leveraging the unique properties of nanomaterials, such as improved drug bioavailability, targeting capabilities, and enhanced imaging capabilities. The emerging markets for nanomedicines and NMDs are experiencing rapid growth, driven by the increasing demand for advanced therapeutic and diagnostic solutions [243].

### Commercialisation of nanomedicines

**Fig. 28:**
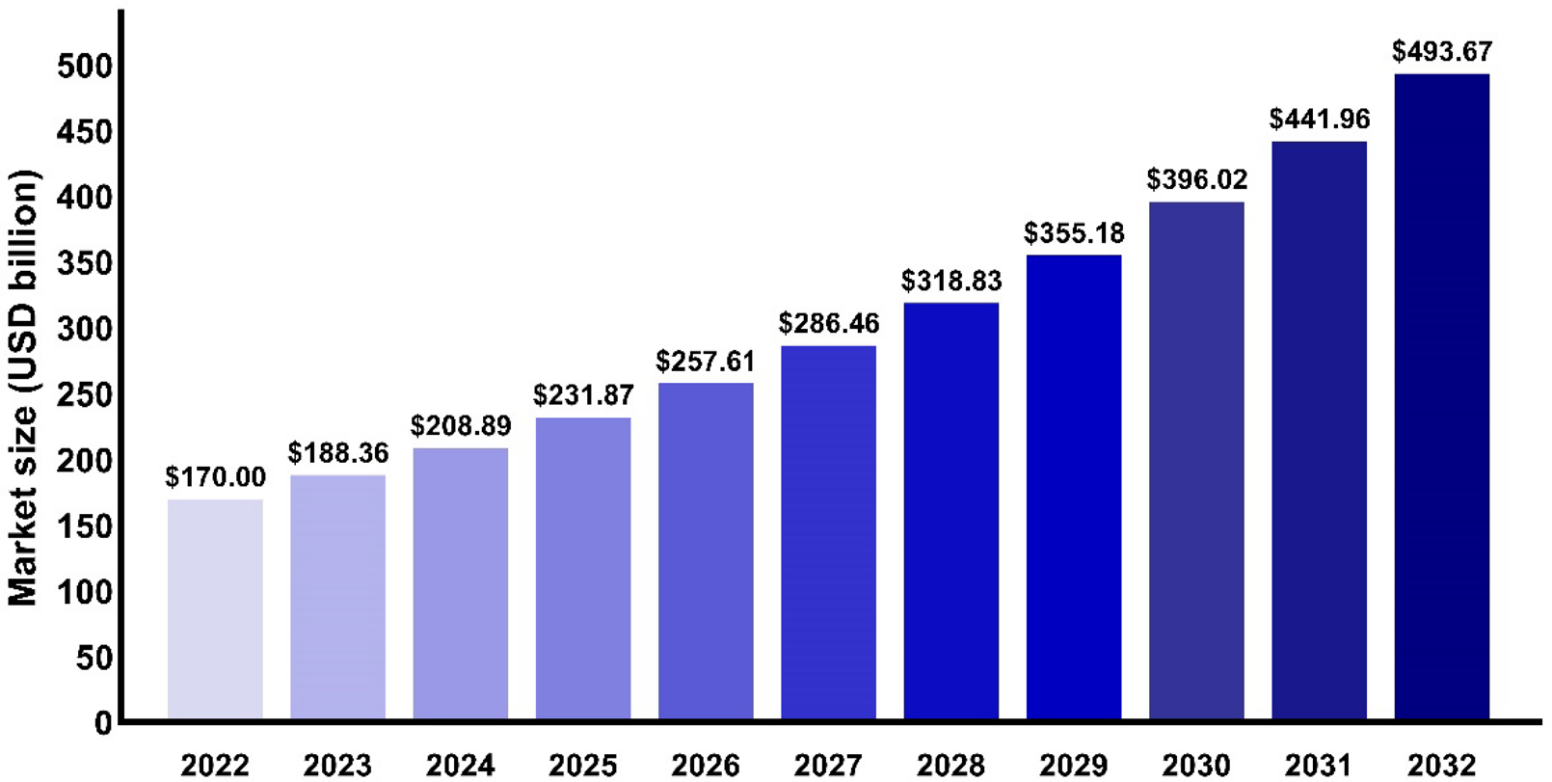
Nanomedicine market size forecast from 2022 to 2032. Adapted from Precedence Research [243]

According to a report by Precedence Research, the global nanomedicine market was valued at approximately $170 billion in 2022 and is expected to reach around $494 billion by 2032 (Fig. 28), growing at a compound annual growth rate (CAGR) of 11.3% from 2023 to 2032 [243]. The rapidly expanding domain now includes over 200 enterprises across the globe, such as Sanofi SA, Pfizer Inc., Taiwan Liposome Company Ltd, Johnson & Johnson, and Bristol-Myers Squibb Company, all of which provide a wide variety of healthcare solutions based on nanotechnology [244]. Considerable financial resources are being allocated annually towards the advancement of nanotechnology R&D, with the total investment in the United States alone reaching approximately $40 billion over the past 20 years [245].

The drug delivery segment held the largest portion of the healthcare nanotechnology (nanomedicine) market in 2022, accounting for 34% of its total, driven by the increasing prevalence of chronic and infectious diseases such as cancer and COVID-19, along with a heightened public understanding of the potential benefits of nanomedicine [246]. Liposomes (spherical vesicles made of phospholipids with the capability to deliver both hydrophilic and hydrophobic drugs) pioneered the successful transition of nanomedicine from concept to commercial reality [247, 248]. Since receiving approval from the FDA in 1995, lipid-based nanomedicines have been on the market for nearly three decades. Out of all the nanomedicines currently available in the market or undergoing different stages of clinical translation, liposomes or lipid-based NPs represent the most common category, adding up to 33% of the total [249]. Beside them, polymer-based nanomedicines, nanocrystals, inorganic-based NPs, carbon-based NPs and protein-based NPs have also gained approval and entered commercialisation [242], as shown in Table 1.

**Table 1:** List of globally marketed nanomedicines approved by FDA, EMA, and NMPA. Adapted with permission from Halwani et al*.* [250]

Type	Trade name	Company	Date of approval	Active ingredients	Indication
Lipid-based NPs	Doxil^®^	Johnson & Johnson	FDA (1995), EMA (1996)	Doxorubicin (adriamycin)	Metastatic ovarian cancer, HIV-associated Kaposi’s sarcoma (KS)
	Lipodox^®^	Sun Pharma Global FZE	FDA (2013)	Doxorubicin hydrochloride	Metastatic ovarian cancer, HIV-associated KS
	DaunoXome^®^	Galen Ltd	FDA, EMA (1996)	Daunorubicin	Cancers and HIV-associated KS
	Onivyde^®^	Merrimack Pharmaceuticals	FDA (2015)	Irinotecan	Metastatic pancreatic cancer
	DepoCyt^®^	Pacira Pharmaceuticals	EMA (2002), FDA (2007)	Cytarabine	Lymphomatous meningitis
	Myocet^®^	Teva Pharmaceutical Industries Ltd	EMA (2000)	Doxorubicin hydrochloride	Breast cancer
	Caelyx^®^	Janssen Pharmaceuticals	EMA (1996)	Doxorubicin	Breast cancer, ovarian cancer, HIV-associated KS
	Mepact^®^	Takeda France SAS	EMA (2009)	Mifamurtide	Osteogenic sarcoma
	Marqibo^®^	Talon Therapeutics	FDA (2012)	Vincristine	Philadelphia chromosome-negative chronic myelogenous leukemia in adult patients
	Onpattro^®^	Alnylam	FDA & EMA (2018)	Patisiran	Hereditary transthyretin (TTR) mediated amyloidosis
	Lipusu^®^	Luye Pharma Group Ltd	FDA (2016)	Paclitaxel	Breast cancer, non-small-cell lung cancer (NSCLC)
	AmBisome^®^	NeXstar Pharmaceuticals	EMA (1990), FDA (1997)	Amphotericin B	Antifungal drug
	Vyxeos^®^	Jazz Pharmaceutics	FDA (2017), EMA (2018)	Daunorubicin and cytarabine	Acute myeloid leukemia
	Abelcet^®^	Defiante Farmaceutica	FDA (1995)	Amphotericin B	Antifungal drug
	DepoDur^®^	SkyePharma	FDA (2004), EMA (2006)	Liposomal morphine sulphate	Postoperative analgesia
	Curosurf^®^	Chiesi	FDA (1999)	Poractant alfa	Respiratory distress syndrome (RDS)
	Zevalin^®^	Bayer Pharma	FDA (2002) Disc. *, EMA (2004)	90Y-ibritumomab tiuxetan	Lymphoma
	Inflexal^®^	Crucell Berna Biotech	EMA (1997)	Inactivated influenza virus vaccine	Prevents influenza infection
	Pfizer-BioNTech vaccine	Pfizer Pharmaceuticals	FDA (2020)	mRNA vaccine	prevents COVID-19 infection
	Moderna COVID-19 Vaccine	ModernaTX Inc	FDA (2020)	mRNA vaccine	Prevents COVID-19 infection
	Visudyne^®^	QLT Phototherapeutics	FDA & EMA (2000)	Photosensitiser (PS), benzoporphyrin	Choroidal neovascularisation caused by wet age-related macular degeneration
Polymer-based NPs	Cimzia^®^	UCB	FDA (2008), EMA (2009)	IgG Fab’ fragment that specifically recognises and binds to TNF-α	Rheumatoid arthritis, Crohn’s disease, psoriatic arthritis, and ankylosing spondylitis
	Apealea^®^	Oasmia Pharmaceutical AB	EMA (2018)	Paclitaxel	Ovarian cancer, peritoneal cancer, fallopian tube cancer
	Adagen^®^	Enzon Pharmaceuticals Inc	FDA (1990)	Adenosine deaminase (ADA)	Adenosine deaminase (ADA)-severe combined immunodeficiency disorder
	Neulasta^®^	Amgen, Inc	FDA (2002)	Filgrastim	Febrile neutropenia, consequent infections arising due to lack of neutrophils
	Oncaspar^®^	Enzon Pharmaceuticals Inc	FDA (1994), EMA (2016)	L-asparaginase	Acute lymphoblastic leukemia, chronic myelogenous leukemia
	Genexol-PM^®^	Lupin Ltd	FDA (2007)	Paclitaxel	Breast cancer
	Pegasys^®^	Genentech USA, Inc	FDA, EMA (2002)	Recombinant human alfa-2a interferon	Hepatitis C, hepatitis B
	Diprivan^®^	Fresenius Kabi	FDA (1989), EMA (2001)	Propofol	(Sedative-hypnotic agent) used in surgery to induce relaxation before and during general anesthesia
	Somavert^®^	Pfizer Pharmaceuticals	EMA (2002), FDA (2003)	Analog of human growth hormone (acts as an antagonist of GH receptors)	Acromegaly
	Macugen^®^	Pfizer Pharmaceuticals	FDA (2004)	Pegatinib sodium	Choroidal neovascularisation caused by wet age-related macular degeneration
	Mircera^®^	Vifor	EMA (2007), FDA (2018)	Epoetin β (EPO) (EPO is a genetically recombinant form of erythropoietin)	Anemia
	PegIntron^®^	Merk & Co. Inc	EMA (2000), FDA (2001)	Alpha interferon (INF) molecule	Hepatitis C
	Krystexxa^®^	Savient Pharmaceuticals	FDA (2010)	Pegloticase is a recombinant porcinelike uricase	Refractory chronic gout
	Plegridy^®^	Biogene	FDA (2014)	Recombinant IFN-β	Relapsing remitting multiple sclerosis (RRMS) in adult patients
	Adynovate^®^	Baxalta US Inc	FDA (2015)	Coagulation factor VIII	Hemophilia A
	Copaxone^®^/FOGA	Teva Pharmaceutical Industries Ltd	FDA (1996), EMA (2016)	Glatiramer acetate	Multiple sclerosis (MS)
	Eligard^®^	Tolmar Pharmaceuticals Inc	FDA (2002)	Leuprolide acetate	Prostate cancer
	Renagel^®^	Sanofi	FDA (2000)	Sevelamer carbonate	Hyperphosphatemia caused by chronic kidney disease (CKD)
	Renagel^®^/renvela^®^	Genzyme	EMA (2007)	Sevelamer HCL	Hyperphosphatemia caused by CKD
	Restasis^®^	Allergan	FDA (2003)	Cyclosporine	Chronic dry eye
	Rebinyn^®^	NovoNordisk	FDA (2017)	Recombinant DNA-derived coagulation FIX	Hemophilia B
	Estrasorb^™^	Novavax, Inc	FDA (2003)	Estradiol (17β-estradiol) hemihydrate	Moderate vasomotor symptoms due to menopause
	Zilretta^®^	Flexion Therapeutics	FDA (2017)	Triamcinolone acetonide	Knee osteoarthritis
Nanocrystals	Emend^®^	Merk & Co. Inc	FDA (2003)	Aprepitant	Antiemetic drug
	Ivemend^®^	Merk & Co. Inc	FDA, EMA (2008)	Fosaprepitant dimeglumine (prodrug of aprepitant)	Antiemetic drug
	Ostim^®^	Osartis GmbH & Co	FDA (2004)	Calcium hydroxyapatite	Bone-grafting material
	Rapamune^®^	Wyeth Pharmaceuticals Inc. (a subsidiary of Pfizer Inc.)	EMA (2001), FDA (2010)	Sirolimus (rapamycin)	Prevents rejection of kidney transplants (immunosuppressant)
	Rapamune^®^	Wyeth Pharmaceuticals Inc. (a subsidiary of Pfizer Inc.)	FDA (2015)	Sirolimus (rapamycin)	A rare progressive lung disease (lymphangioleiomyomatosis)
	Vitoss^®^	Orthovita Inc	FDA (2003)	β-tricalcium phosphate	Bone-grafting material
	Ritalin LX^®^	Novartis	FDA (2002)	Methylphenidate	Attention deficit hyperactivity disorder (ADHD) in children
	Avinza^®^	Pfizer Pharmaceuticals	FDA (2002)	Morphine sulfate	Psychostimulant
	Focalin XR^®^	Novartis	FDA (2008)	Dexamethylphenidate HCl	ADHD in children
	Invega^®^	Janssen Pharmaceuticals	FDA (2009)	Paliperidone	Schizophrenia
	Invega sustenna^®^	Janssen Pharmaceuticals	FDA (2009)	Paliperidone Palmitate	Schizophrenia
	Megace ES^®^	Par Pharmaceuticals	FDA (2005)	Megestrol acetate	Antianorexic
	NanOss^®^	RTI Surgical	FDA (2005)	Hydroxyapatite	Bone substitute
	EquivaBone^®^	Zimmer Biomet	FDA (2009)	Hydroxyapatite	Bone substitute
	OsSatura^®^	Isotis Othobiologics Inc	FDA (2003)	Hydroxyapatite	Bone substitute
	Epaxal^®^	Crucell Berna Biotech	EMA (1993)	Inactivated hepatitis A virus vaccine	Prevents hepatitis A infection
	Zanaflex^®^	Acorda	FDA (2002)	Tizanidine HCl	Muscle relaxant
	Ryanodex^®^	Eagle pharm	FDA (2014)	Dantrolene sodium	Malignant hyperthermia
	TriCor^®^	Abbott Laboratories	FDA (2004)	Fenofibrate	Antihyperlipidemia
Inorganic NPs	Feraheme^™^	AMAG Pharmaceuticals	FDA (2009)	Ferumoxytol	Anemia
	Venofer^®^	Luitpold Pharm	FDA (2000)	Iron sucrose	Iron deficiency in CKD
	Dexferrum^®^	American Regent	FDA (1996)	Iron dextran	Iron deficiency in CKD
	Ferinject^®^	Vifor	FDA, EMA (2013)	Iron carboxymaltose colloid	Iron deficient anemia
	Ferrlecit^®^	Sanofi-Aventis	FDA (1999), EMA (2013)	Sodium ferric gluconate	Iron deficiency in CKD
	Hensify^®^	Nanobiotix	EMA (2019)	Hafnium oxide NPs	Locally advanced squamous cell carcinoma
	Infed^®^	Actavis Pharma	FDA (1992)	Iron dextran	Iron deficiency in CKD
	Feridex^®^/endorem^®^	AMAG Pharma	FDA (1996) Disc.* 2008	SPION-dex	Imaging agent
	GastroMARK^™^/umirem^®^	Mallinckrodt Inc	FDA (2009)Disc.* 2012	SPION-silicone	Imaging agent
Carbon-based NPs	Canalin^®^	Chongqing Lummy Pharmaceutical Co., Ltd	NMPA (2020)	Carbon NPs	Lymph node tracers [251]
Protein-based NPs	Abraxane^®^	Celgene Pharmaceutical Co. Ltd	FDA (2005, 2012, 2013), EMA (2008)	Paclitaxel	Approved by the FDA for treatment of metastatic breast cancer (2005), lung cancer (2012), and metastatic pancreatic adenocarcinoma (2013)
	Ontak^®^	Eisai	FDA (1999)	Diphtheria toxin	Leukemia, T-cell lymphoma

^*^Disc.: discontinued

### Commercialisation of nanotechnology-based medical devices (NMDs)

In addition to nanomedicines, NMDs are being approved for marketing worldwide. The first FDA 510(k) clearance for a medical device containing nanomaterials was given in 1980 [252]. A 510(k) is a premarket submission made to the FDA to demonstrate that the device to be marketed is as safe and effective (i.e., substantially equivalent) as a legally marketed device [253]. As of June 2024, 151 FDA 510(k)-cleared products featured the term “nano” in either the manufacturer or product name. However, not all of these products are NMDs. Table 2 lists those with published evidence confirming the use of nanomaterials or nanotechnology, including bone fillers (nano-structured hydroxyapatite), and dental resins (dimer acid derived nanohybrid composite). It also covers devices featuring nano-coatings or nano-structured surfaces, like dental implants (calcium phosphate coating), intervertebral fusion devices (hierarchical nano/microtextured surface), knee prostheses (hydroxyapatite nanocoating), vessel sealing devices (non-stick coating), trauma screws (surface with antimicrobial nanotopography) [254].

**Table 2:** List of NMDs approved by FDA for marketing through the 510(k) process. Extracted from 510(k) Premarket Notification database https://www.accessdata.fda.gov/scripts/cdrh/cfdocs/cfpmn/pmn.cfm (keyword: nano)

Medical application/specialty	Device/trade name	Applicant	Date of approval	Nanomaterial	Indication
Anesthesiology	UniPlex NanoLine cannula, PlexoLong-NanoLine set, StimuLong-NanoLine Set	Pajunk GmbH	01/12/2006	Nanoparticle-enhanced surface layer	Anesthesia conduction kits for temporary anesthetic delivery to peripheral nerve bundles during regional anesthesia
	SonoLong Sono, Curl Sono, NanoLine^®^	PAJUNK GmbH Medizintechnologie	03/01/2012	Polymer nanocoating	Anesthesia conduction kits for delivery of continuous conduction anesthesia and/or analgesia of peripheral nerves
Clinical chemistry	Nanosep^™^ N10	Intercept, Ltd	04/26/1994	Nanoporous structures membrane	Clinical sample concentrator, for the concentration and purification of biological samples such as peptides, proteins, oligonucleotides, DNA, and RNA
	Nano-Check^™^ AMI 3 in 1 Cardiac Disease Test	Nano-Ditech Corporation	03/02/2006	Colloidal Au NPs	Immunoassay method, a one-step lateral flowimmuno chromatography assay for the qualitative determination of three cardiac markers simultaneously in serum and heparin plasma
	Nano-Check^™^ AMI 2 in 1 Cardiac Marker, cTnI and Myoglobin	Nano-Ditech Corporation	10/20/2010	Colloidal Au NPs	Immunoassay method, to provide rapid and reliable results in diagnosing acute myocardial infarction (heart attack) by measuring the levels of cTnI and Myoglobin in the blood
	Nano-Check^™^ AMI cTnI Cardiac Marker Test	Nano-Ditech Corporation	10/20/2010	Colloidal Au NPs	A rapid immunoassay for the qualitative determination of cardiac troponin I (cTnI) in human serum and plasma specimens at cut off, as an aid in the diagnosis of Acute Myocardial Infarction (AMI)
Dental	NanoTite PREVAIL, Certain, Parallel Walled, Tapered, External Hex	BIomet 3i, Inc	01/31/2008	Osseotite^®^ nanoscale surface and nanocrystalline deposition of calcium phosphate	A root-form endosseous implant, for surgical placement in the jaw to support prosthetic attachments for single-tooth restorations, partially or fully edentulous spans, bridgework, and overdentures
	Renamel^®^ NANO^™^ + plus	Cosmedent, Inc	02/24/2017	Multifunctional acrylic monomers and silicaceous nanofillers	Tooth shade resin material, designed for Class I-V dental restorations, direct veneering of anterior teeth, splinting, and the repair of composite or ceramic restorations
	Nano composite	Cosmedent, Inc	04/16/2007	Light-cure nanocomposite	Tooth shade resin material, possesses the physical and mechanical properties to function in the oral cavity, mimicking the esthetic qualities of natural teeth
	DMRC NanoFlow; DMRC Nanocomposite; ebond; sure etch gel; sure etch liquid	Danville Materials, Inc	09/25/2012	Light-cure NPs	DMRC NanoFlow, a flowable composite for small cavities, repairs, linings, sealants, and marginal repairs; DMRC NanoComposite, a universal composite for direct and indirect restorations; eBond, a bonding agent to enhance adhesion between composites and tooth structure; Sure Etch Gel and Sure Etch Liquid, both used to prepare tooth surfaces for improved bonding by creating micropores
	Prime & Bond^®^ NT™ Dual Cure Nano-Technology Universal Dental Adhesive System	Dentsply International, Inc	02/23/2005	Nanofiller	Tooth bonding agent for bonding all indirect restorations, including metal, ceramic, and composite crowns, inlays, onlays, veneers, bridge retainers, and endodontic posts to enamel and dentin without light
	Nanocomposite Restorative Kit	DMG USA, Inc	05/30/2008	Light-cure nanocomposite	Tooth shade resin material, designed for restorative procedures to repair and restore the function and aesthetics of teeth affected by cavities or other forms of dental damage
	NanoCeram-Bright	DMP, Ltd	11/19/2010	Light-cure nano-hybrid composite	Resin material for anterior and posterior restorations, cervical caries, root erosion, wedge-shaped defects, veneering discoloured anterior teeth, splinting mobile teeth, and repairing composite and ceramic veneers
	Nano Varnish, Plaquit, Lightpaint on Surface	Dreve Dentamid GmbH	04/05/2018	Light-cure NPs	Dental sealing lacquer for coating resin temporaries
	Nanotite^TM^ Dental Implants	Implant Innovations, Inc	02/22/2007	Calcium phosphate nanocrystal	Dental implants with nanoscale calcium phosphate crystals on the surface
	Series of Nanova products for dental use	Nanova Biomaterials Inc	01/16/2018 etc.*	Hydroxyapatite nanofiber	Tooth bonding agents; sealant, pit and fissure, conditioner; tooth shade resin materials
	Nano-TiCrown^™^	Nano-Write Corporation	06/11/2003	Nanostructured titanium/titanium nitride material	Base metal alloy for making single-unit porcelain-fused-to-metal (PFM) prosthetics and custom dental prosthetics, such as porcelain-to-metal veneers
	Dimer Acid Derived Nano-hybrid Composite Restorative Material	Novocol, Inc	09/13/2007	Dimer acid derived nanohybrid composite	Tooth shade resin material, for class I, II, III, IV and V cavity classes
	NanoGen^®^	Orthogen, Llc	05/06/2011	Nanocrystalline calcium sulfate	Bone grafting material for bone regeneration, used alone, mixed with other bone filling agents to prevent particle migration, or as a resorbable barrier over other graft materials
	Pac-Dent Ceramic Nanohybrid Resin	Pac-Dent, Inc	07/26/2021	Ceramic nanohybrid	Tooth shade resin material, for fabricating permanent restorations such as inlays, onlays, veneers and full crownrestorations
	Nano-Bond II Adhesive System	Pentron Clinical Technologies	08/10/2007	Light-cure nanocomposite	Resin tooth bonding agent
	Sculpture^®^ Plus Nano-Hybrid Composite	Pentron Laboratory Technologies	01/17/2003	Nanohybrid composite	Dental restorative material used to restore carious lesions, structural defects, or lost tooth structure
	CalApex Calplus, Cerafill RCS, Endoseal, Nanoseal S, Zical Ultra Paste, Zical Ultra Powder/Liquid	Prevest Denpro, Ltd	05/23/2022	Ag NPs	Antibacterial root canal sealer fortified with nanosilver used in conjunction with gutta-percha for effective sealing root canals of permanent teeth
	Camouflage^™^ NanoHybrid Composite (Universal and Flowable)	Prismatik Dentalcraft, Inc	06/04/2014	Ceramic-filled nanohybrid resin composite material	Tooth shade resin material for Class I-VI cavities in anterior and posterior teeth, suitable for direct restorations, inlays, and onlays
	Capo Hybrid, Capo Slow Flow, Capo Flow, Capo Natural, Capo Universal, Nano Paq, Nano Paq Flow	Schütz Dental GmbH	10/24/2016	Light-cure nanocomposite	Tooth shade resin material for direct and indirect restorations, fissure sealing on premolars and molars, stump buildup, and shape and colour correction
General & plastic surgery	Series of LigaSure^™^ products	Covidien	03/23/2021 etc.*	Nonstick nanocoating	Electrosurgical cutting and coagulation device and accessories, for open surgical procedures where ligation and division of vessels, tissue bundles, and lymphatics
	Medline ReNewal Reprocessed LigaSure Exact Dissector, Nano-coated (LF2019)	Surgical Instrument Service And Savings, Inc	04/29/2024	Nanocoating to reduce tissue sticking	Electrosurgical cutting and coagulation device and accessories
Immunology	Nanopia Wide Range C-Reactive ProTein (CRP) Reagent Kit	Clinical Data, Inc	02/09/2006	Antibody-coated latex particles	Quantitative latex agglutination immunoassay for measuring C-Reactive Protein (CRP) in serum or plasma, used to evaluate infection, tissue injury, and inflammatory disorders alongside a complete clinical evaluation
Microbiology	Nano-Check^™^ COVID-19 Antigen Test	Nano-Ditech Corporation	01/23/2024	Au NPs	Simple point-of-care device, used as an immunochromatographic lateral flow assay for detection of SARS-CoV-2 nucleo protein antigens in human anterior nasal swab specimens
Neurology	Series of Titan NanoLOCK products for neurology	Medtronic Sofamor Danek USA, Inc	09/29/2022 etc.*	Titanium nanoscale featured surface	Stereotaxic Instruments, to be used during preparation and placement of Medtronic implants during spinal surgery to assist in precisely locating anatomical structures in either open, or minimally invasive, procedures
Orthopedic	Ascend VBR System, Ascend NanoTec VBR System	Alphatec Spine, Inc	10/06/2023	Hydroxyapatite nanocoating	Spinal intervertebral body fixation orthosis to replace a collapsed, damaged, or unstable vertebral body or for reconstructive decompression of the spinal cord and neural tissues in degenerative disorders
	Calibrate PSX Interbody System, Calibrate NanoTec PSX Interbody System	Alphatec Spine, Inc	07/13/2023	Hydroxyapatite nanocoating	Intervertebral body fusion device for spinal fusion from L1 to S1 in skeletally mature patients, treating symptomatic degenerative disc disease (DDD), degenerative spondylolisthesis, and/or spinal stenosis at one or two adjacent levels
	Series of IdentiTi Porous Ti Interbody Systems	Alphatec Spine, Inc	02/22/2024 etc.*	Hydroxyapatite nanocoating	Intervertebral body fusion devices for treating DDD and spinal instability in skeletally mature patients
	nanOss^®^ Bone Void Filler	Angstrom Medica, Inc	02/03/2005	Hydroxyapatite nanocrystal	Resorbable calcium salt bone void filler device for non-structural bony voids or gaps in the extremities, spine, and pelvis
	Series of NanoBone^®^ products	Artoss GmbH	04/23/2019 etc.*	Nanocrystalline osteoconductive hydroxyapatite	Resorbable calcium salt bone void fillers device for bony voids or gaps in the extremities, posterolateral spine, and pelvis
	EMPOWR Porous Femur with HAnano Surface^TM^	Encore Medical, L.P	03/30/2021	Hydroxyapatite nanocoating	Knee joint patellofemorotibial metal/polymer porous-coated uncemented prosthesis for total knee arthroplasty, treating arthritis, avascular necrosis, joint configuration loss, deformities, fractures, and for salvage of failed surgeries
	NanoHive Medical Lumbar Interbody System	Ian Helmar	07/12/2023	Soft Titanium^®^ lattice nanotextured surface	Intervertebral body fusion device for use in skeletally mature patients with DDD at one or two contiguous levels from L2 to S1
	Series of Titan NanoLOCK products for orthopedic	Medtronic Sofamor Danek USA, Inc	10/06/2022 etc.*	Titanium nanoscale featured surface	Intervertebral body fusion device for spinal fusion in skeletally mature patients with symptomatic DDD, degenerative spondylolisthesis, and/or spinal stenosis at one or two levels from L2 to S1
	Series of Nanova products for orthopedic use	Nanova Biomaterials Inc	05/05/2017 etc.*	Hydroxyapatite nanofiber	Metallic bone fixation appliances and accessories for securing soft tissue or bone-tendon-bone grafts during cruciate ligament reconstruction surgeries of the knee
	Series of Nanovis systems	Nanovis, Llc	06/02/2023 etc.*	Bio-ceramic nanotube surface	Intervertebral body fusion devices for immobilising and stabilising spinal segments as an adjunct to fusion in patients with DDD, spinal stenosis, spondylolisthesis, spinal deformities, trauma, tumours, pseudarthrosis, and failed fusions
	Series of nanOss^™^ products	Pioneer Surgical Technology, Inc	10/17/2014 etc.*	Nano-structured hydroxyapatite	Bone void fillers
	Series of SeaSpine Systems	SeaSpine Orthopedics Corporation	07/07/2021 etc.*	Titanium nanoscale featured surface	Intervertebral body fusion devices
	OsteoFlo^®^ NanoPutty^®^-Quadphasic Synthetic Bone Graft	SurGenTec, Llc	08/14/2020	Hydroxyapatite NPs	Resorbable calcium salt bone void filler device
	Tyber Medical BioTy^®^ Nanotopography Trauma Screw	Tyber Medical, Llc	06/20/2017 11/16/2016	Nanoscale featured surface	Metallic bone fixation fastener for bone reconstruction, osteotomy, arthrodesis, and joint fusion
Radiology	DRX-Revolution Nano Mobile X-ray System	Carestream Health, Inc	02/05/2018 etc.*	Carbon nanotube	Diagnostic mobile x-ray system with digital radiography (DR) technology, featuring a self-contained x-ray generator, image receptor(s), imaging display, and software for acquiring diagnostic images outside a standard x-ray room
	Nanox.ARC	Nanox Imaging, Ltd	04/28/2023	Nano-cones field on a silicon chip	Tomographic X-ray system for producing images of the human musculoskeletal system from a single sweep, as an adjunct to conventional radiography for adult patients
	Nanox Cart X-ray System	Nanox Imaging, Ltd	04/01/2021	Nano-cones field on a silicon chip	Mobile X-ray system for radiographic examinations of hands, wrists, and fingers in adult patients, designed for use when transporting the patient to fixed equipment is not feasible
	Nanor^®^ and Efficast/Nanor Hybrid Thermoplastic Materials	Orfit Industries N.V	09/26/2013	Hybrid thermoplastic NPs	Medical charged-particle radiation therapy system for retaining and reproducing a patient's position during radiation therapy
Toxicology	Nano-Check^™^ DAT 5 Multi Drug Screening Test	Nano-Ditech Corporation	05/15/2005	Colloidal Au NPs	Qualitative immunoassay: a one-step, type II competitive immunochromatographic assay for detecting cannabinoids, opiates, benzoylecgonine, methamphetamine, phencyclidine, and their metabolites in human urine

^*^Disc.: Due to the complex regulatory process and need for continuous improvement, medical devices often undergo multiple revisions and approvals, resulting in several approval dates throughout their life cycle

Some devices gain FDA approval via a different regulatory pathway, the premarket approval (PMA) process. The PMA process requires clinical trials to demonstrate the safety and efficacy of a device and is the most stringent type of device marketing application required by the FDA [252]. As of June 2024, five manufacturers had their 75 products, each bearing “nano” in the name and including different sizes or models of the identical device, approved via the PMA process [255]. Among these products, only the COBRA PzF NanoCoated Coronary Stent demonstrated clear evidence of using nanotechnology (Table 3). This particular product features a cobalt-chromium strut enveloped in a poly [bis(trifluoroethoxy)phosphazene] (Polyzene-F^®^ polymer) coating with a thickness of approximately 50 nm, which has thrombo-resistant, anti-inflammatory and rapid healing effects [256]. Exploring the 510(k) and PMA databases reveals that FDA-approved NMDs are predominantly used in orthopedic and dental applications.

**Table 3:** List of the NMD approved by FDA for marketing through the PMA process. Extracted from Premarket Approval (PMA) database https://www.accessdata.fda.gov/scripts/cdrh/cfdocs/cfpma/pma.cfm (keyword: nano)

Medical application/specialty	Device/trade name	Applicant	Decision date	Withdrawal date	Nanomaterial	Indication
Cardiovascular	Cobra PzF NanoCoated Coronary Stent System	Celonova Biosciences, Inc.	02/21/2017 etc.*	12/22/2022	Polyphosphazene nanocoating	Coronary stent system for improving coronary luminal diameter in patients, including those with diabetes mellitus, with symptomatic ischemic heart disease from de novo lesions in native coronary arteries

^*^Disc.: Due to the complex regulatory process and need for continuous improvement, medical devices often undergo multiple revisions and approvals, resulting in several decision dates throughout their life cycle

NMDs approved by NMPA show a distinct emphasis in their applications compared to those cleared by FDA, which might be attributed to the variances in healthcare market demands, industrial chains, intellectual property rights, and regulatory frameworks between China and the United States. By June 2024, NMPA-approved NMDs from Chinese manufacturers are predominantly aimed at diagnostic purposes (Table 4), featuring in vitro diagnostic kits with elements like CNTs, gold nanocages, nano-enzymes, rare-earth nanospheres, and magnetic nanobeads for identifying conditions such as myocardial infarction, renal disease, cancer, blood clots, and COVID-19 [257]. The approval list of domestic products also comprises medical dressings containing Ag NPs or nano-ceramic materials, and bone fillers made with nano-structured hydroxyapatite. For the smaller number of overseas products approved (Table 5), diagnostic devices also represent the largest category. Furthermore, several of these products, including Adaptix^™^ Interbody System with Titan nanoLOCK^™^ Surface Technology [258], Nano-coated LigaSure^™^ Maryland Jaw Thoracic Sealer/Divider [259], and Nano-technology Universal Dental Adhesive [260], had already been approved by FDA.

**Table 4:** List of domestic NMDs approved by NMPA for marketing. Extracted from NMPA database https://www.nmpa.gov.cn/datasearch/home-index.html#category=ylqx (Keywords: ‘纳米’ and its equivalent term in English, ‘nano’)

Device/trade name	Applicant	Date of approval	Management category	Nanomaterial	Description
Urinary microalbumin detection kit (carbon nanotube colloidal gold method)	Benxi Tystj Biotechnology, Ltd.	05/06/2020	Class II	CNTs	Kit for qualitative urine microalbumin, used to diagnose kidney diseases
Series of thromboelastography assay kits (nano-enzyme clotting method)	Chongqing Kangjuquan Hong Biotechnology, Ltd.	28/04/2024 etc.*	Class II	Nanoenzyme	Kit for thromboelastography (TEG) instrument to test coagulation in sodium citrate anticoagulated whole blood
Soluble ST2 protein (ST2) assay kit (nano-enzyme fluorescent immunoassay)	Chongqing Kangjuquan Hong Biotechnology, Ltd.	09/04/2024	Class II	Nanoenzyme	Kit to measure soluble suppression of tumourigenicity 2 protein (ST2) levels in human serum
Brain natriuretic peptide (BNP) Assay kit (nano-enzyme fluorescent immunoassay)	Chongqing Kangjuquan Hong Biotechnology, Ltd.	17/04/2023	Class II	Nanoenzyme	Kit to measure B-type natriuretic peptide (BNP) levels in human plasma
Activated coagulation assay kit (nano-enzyme coagulation method)	Chongqing Kangjuquan Hong Biotechnology, Ltd.	01/03/2023	Class II	Nanoenzyme	Kit for TEG to measure R, K, Angle, and MA values in whole blood samples
Thromboelastography quality control (nano-enzyme coagulation method)	Chongqing Kangjuquan Hong Biotechnology, Ltd.	14/02/2023	Class II	Nanoenzyme	Kit for quality control of R, K, Angle, and MA values in TEG testing
Nano-enzyme immunoassay analyser	Chongqing Kangjuquan Hong Biotechnology, Ltd.	07/07/2022	Class II	Nanoenzyme	Analyser using nanoenzyme immunochromatography for quantitative detection of serum amyloid A in serum and plasma
Cardiac troponin I/Creatine Kinase-MB/Myoglobin/D-Dimer/N-terminal pro-B-type Natriuretic Peptide (cTnI/CKMB/Myo/D-Dimer/NT-proBNP) Rapid Test Kit (rare earth nanofluorescence Immunochromatography)	Hunan Naquan Aode Biotechnology, Ltd.	25/11/2022	Class II	Nanofluorescent microsphere	Kit for in vitro quantification of cardiac troponin I/creatine kinase-MB/myoglobin/D-dimer/N-terminal pro-brain natriuretic peptide (cTnI/CKMB/Myo/D-Dimer/NT-proBNP) in plasma and whole blood
Procalcitonin/Interleukin-6 (PCT/IL-6) Rapid Test Kit (rare earth nanofluorescence immunochromatography)	Hunan Naquan Aode Biotechnology, Ltd.	01/11/2022	Class II	Nanofluorescent microsphere	Kit for in vitro quantification of procalcitonin/interleukin 6 (PCT/IL-6) in serum, plasma, and whole blood, aiding in bacterial infection diagnosis
Carcinoembryonic Antigen Test Kit (nanoenzyme immunochromatography)	Hunan Naquan Aode Biotechnology, Ltd.	18/06/2021	Class II	Nanoenzyme	Kit for qualitative detection of carcinoembryonic antigen (CEA) in nipple discharge, used for monitoring breast cancer treatment, prognosis, and recurrence
Interleukin-6 (IL-6) Test Kit (rare earth nanofluorescent immunochromatography)	Hunan Naquan Aode Biotechnology, Ltd.	26/04/2022	Class II	Nanofluorescent microsphere	Kit for quantifying interleukin-6 (IL-6) in serum, plasma, and whole blood to monitor immune status and inflammation
S100-β Protein (S100-β) Test Kit (rare earth nano fluorescent immunochromatography)	Hunan Naquan Aode Biotechnology, Ltd.	26/04/2022	Class II	Nanofluorescent microsphere	Kit for quantifying S100-β protein in serum, aiding in brain injury diagnosis
Heart-type Fatty Acid Binding Protein (H-FABP) Test Kit (rare earth nano fluorescent immunochromatography)	Hunan Naquan Aode Biotechnology, Ltd.	26/04/2022	Class II	Nanofluorescent microsphere	Kit for quantifying heart-type fatty acid-binding protein (H-FABP) in serum, plasma, and whole blood, aiding in acute myocardial infarction diagnosis
Pepsinogen I (PGI)/Pepsinogen II (PGII) Test Kit (rare earth nano fluorescent immunochromatography)	Hunan Naquan Aode Biotechnology, Ltd.	25/05/2022	Class II	Nanofluorescent microsphere	Kit for quantifying pepsinogen I (PGI) and pepsinogen II (PGII) in serum and plasma, used to assess gastric function and fundus gland lesions
Serum Amyloid A (SAA) Test Kit (rare earth nano fluorescent immunochromatography)	Hunan Naquan Aode Biotechnology, Ltd.	26/04/2022	Class II	Nanofluorescent microsphere	Kit for quantifying serum amyloid A (SAA) in serum, plasma, and whole blood, used as non-specific inflammation marker
Anti-Müllerian Hormone (AMH) Test Kit (rare earth nano fluorescent immunochromatography)	Hunan Naquan Aode Biotechnology, Ltd.	26/04/2022	Class II	Nanofluorescent microsphere	Kit for quantifying anti-Müllerian hormone (AMH) in serum, plasma, and whole blood, indicating ovarian reserve function
β2-Microglobulin (β2-MG) Test Kit (rare earth nano fluorescent immunochromatography)	Hunan Naquan Aode Biotechnology, Ltd.	26/04/2022	Class II	Nanofluorescent microsphere	Kit for quantifying β2 microglobulin (β2-MG) in serum, plasma, and venous whole blood, used to monitor proximal renal tubule function
Cardiac Troponin I/Creatine Kinase-MB/Myoglobin (cTnI/CK-MB/Myo) Assay Kit (rare earth nano fluorescent immunochromatography)	Hunan Naquan Aode Biotechnology, Ltd.	26/04/2022	Class II	Nanofluorescent microsphere	Kit for quantifying cardiac troponin I/creatine kinase-MB/myoglobin (cTnI/CKMB/Myo) in serum, plasma, and venous whole blood, used to aid in diagnosing myocardial infarction and myopathy
25-Hydroxy Vitamin D (25-OH VD3) Assay Kit (rare earth nano fluorescent immunochromatography)	Hunan Naquan Aode Biotechnology, Ltd.	26/04/2022	Class II	Nanofluorescent microsphere	Kit for quantifying 25-hydroxyvitamin D3 (25-OH VD3) in serum, plasma, and venous whole blood, used to diagnose vitamin D deficiency
Neutrophil Gelatinase-Associated Lipocalin (NGAL) Assay Kit (rare earth nano fluorescent immunochromatography)	Hunan Naquan Aode Biotechnology, Ltd.	26/04/2022	Class II	Nanofluorescent microsphere	Kit for quantifying neutrophil gelatinase-associated lipocalin (NGAL) in urine, used to assess kidney function damage
Myoglobin (Myo) Assay Kit (rare earth nano fluorescent immunochromatography)	Hunan Naquan Aode Biotechnology, Ltd.	26/04/2022	Class II	Nanofluorescent microsphere	Kit for quantifying myoglobin in serum, plasma, and whole blood, aiding in myocardial infarction diagnosis
N-terminal Pro-brain Natriuretic Peptide (NT-proBNP) Assay Kit (rare earth nano fluorescent immunochromatography)	Hunan Naquan Aode Biotechnology, Ltd.	26/04/2022	Class II	Nanofluorescent microsphere	Kit for quantifying N-terminal pro-brain natriuretic peptide (NT-proBNP) in serum, plasma, and whole blood, aiding in heart failure diagnosis
Creatine Kinase-MB (CK-MB) Assay Kit (rare earth nano fluorescent immunochromatography)	Hunan Naquan Aode Biotechnology, Ltd.	26/04/2022	Class II	Nanofluorescent microsphere	Kit for quantifying creatine kinase-MB (CKMB) in serum, plasma, and whole blood, aiding in diagnosing myocardial infarction and myopathy
Urinary Albumin (U-ALB) Assay Kit (rare earth nano fluorescent immunochromatography)	Hunan Naquan Aode Biotechnology, Ltd.	26/04/2022	Class II	Nanofluorescent microsphere	Kit for quantifying urinary albumin (U-ALB) in urine, aiding in kidney disease diagnosis
Cystatin C (CysC) Assay Kit (rare earth nano fluorescent immunochromatography)	Hunan Naquan Aode Biotechnology, Ltd.	26/04/2022	Class II	Nanofluorescent microsphere	Kit for quantifying cystatin C (CysC) in serum, plasma, and whole blood, indicating glomerular filtration rate
High-sensitivity C-reactive Protein (hsCRP + Conventional CRP) Assay Kit (rare earth nano fluorescent immunochromatography)	Hunan Naquan Aode Biotechnology, Ltd.	26/04/2022	Class II	Nanofluorescent microsphere	Kit for quantifying C-reactive protein (CRP) in serum, plasma, and whole blood
Procalcitonin (PCT) Assay Kit (rare earth nano fluorescent immunochromatography)	Hunan Naquan Aode Biotechnology, Ltd.	26/04/2022	Class II	Nanofluorescent microsphere	Kit for quantifying procalcitonin (PCT) in serum, plasma, and whole blood, aiding in diagnosing bacterial infectious diseases
D-Dimer Assay Kit (rare earth nano fluorescent immunochromatography)	Hunan Naquan Aode Biotechnology, Ltd.	26/04/2022	Class II	Nanofluorescent microsphere	Kit for quantifying D-dimer in plasma and whole blood, aiding in diagnosing venous thrombosis, DIC, and monitoring thrombolytic therapy
Cardiac Troponin I (cTnI) Assay Kit (rare earth nano fluorescent immunochromatography)	Hunan Naquan Aode Biotechnology, Ltd.	26/04/2022	Class II	Nanofluorescent microsphere	Kit for quantifying cardiac troponin I (cTnI) in serum, plasma, and whole blood, aiding in myocardial infarction diagnosis
Glycated Hemoglobin (HbA1c) Assay Kit (rare earth nano fluorescent immunochromatography)	Hunan Naquan Aode Biotechnology, Ltd.	26/04/2022	Class II	Nanofluorescent microsphere	Kit for quantifying glycated hemoglobin (HbA1c) in whole blood, aiding in diabetes diagnosis and glucose monitoring
Series of rare earth nano fluorescent immunoassay analysers	Hunan Naquan Aode Biotechnology, Ltd.	10/06/2022 etc.*	Class II	Nanofluorescent microsphere	Analyser for qualitative and quantitative analysis of analytes in human samples with compatible reagents
Cardiac Troponin I (cTnI) Detection Kit (nanogold cage colloidal gold method)	Nanjing Botian Kezhi Biotechnology, Ltd.	05/02/2021	Class II	Gold nanocages	Kit for qualitative detection of cardiac myosin-binding protein C in plasma
Troponin C (cTnC) Detection Kit (nanogold cage colloidal gold method)	Nanjing Botian Kezhi Biotechnology, Ltd.	27/08/2021	Class II	Gold nanocages	Kit for qualitative detection of cardiac myosin-binding protein C in plasma
Nano acupoint dressing	Shanghai Manjici Biology Limited Company	29/01/2021	Class II	Nano far-infrared ceramic	Dressing relieves pain from periarthritis, sprains, dysmenorrhea, cervical pain, joint pain, breast hyperplasia, and mastitis
Nano-silver medical antimicrobial dressing	Shenzhen Ajet Pharmaceutical Technology, Ltd.	23/04/2021	Class III	Ag NPs	Dressing protects burns and scalds, with nano-silver reducing infections
Nano-silver antimicrobial adhesive dressing	Shenzhen Ajet Pharmaceutical Technology, Ltd.	27/05/2021	Class III	Ag NPs	Dressing protects surgical incisions, with nano-silver as an antibacterial
Medical Nano Hydroxyapatite/Polycaprolactone 66 Composite Bone Filling Material	Sichuan Guona Keji, Ltd.	14/11/2022	Class III	Hydroxyapatite NPs	Bone filling material for repairing defects from cysts, tumours, fractures, spinal fusion, and joint grafting
Nano-silver burn dressing	Wuhan Canvest Biotechnology, Ltd.	29/10/2021	Class III	Ag NPs	Dressing for burns and scalds, with nano-silver reducing infections
Nano-silver wound dressing	Wuhan Canvest Biotechnology, Ltd.	02/08/2021	Class III	Ag NPs	Dressing with antibacterial nano-silver layer, adhesive, absorbent layer, release paper, and barrier paper; reduces wound infections
Novel Coronavirus (2019-nCoV) IgM/IgG Antibody Test Kit (rare earth nanofluorescence immunoassay)	Xiamen AmonMed Biotechnology, Ltd.	22/04/2022	Class III	Nanofluorescent microsphere	Kit for qualitative detection of 2019-nCoV IgM/IgG antibodies in serum from unvaccinated and uninfected individuals
Nano-silver burn dressing	Anxin Nano Biotechnology (Zhuhai), Ltd.	02/08/2021	Class III	Ag NPs	Burn dressing for second-degree burns, scalds, ulcers, and wounds to reduce infections
Nano silver dressing	Anxin Nano Biotechnology (Zhuhai), Ltd.	02/08/2021	Class III	Ag NPs	Wound dressing to reduce infections

^*^Disc.: Due to the complex regulatory process and need for continuous improvement, medical devices often undergo multiple revisions and approvals, resulting in several decision dates throughout their life cycle

**Table 5:** List of imported NMDs approved by NMPA for marketing. Extracted from NMPA database https://www.nmpa.gov.cn/datasearch/home-index.html#category=ylqx (Keywords: '纳米' and its equivalent term in English, ‘nano’)

Device/trade name	Applicant	Date of approval	Management category	Nanomaterial	Description
Nanofilled self adhesive light cured protective coating	GC Corporation	28/08/2019	Class II	Nanoscale light-cure filler	Dental coating material enhances gloss and hardness, protects glass ionomer cement and composite resin, and seals the bonding interface
Nano-checker 710	Nano-Ditech Corporation	21/10/2019	Class II	Colloidal Au NPs	Analytical equipment quantitatively detects analytes in serum, plasma, or blood, including troponin I, myoglobin, CK-MB, procalcitonin, D-dimer, and NT-proBNP
In Vitro Nano-CheckTM AMI 2 in 1 Cardiac Marker, cTnI and Myoglobin	Nano-Ditech Corporation	23/11/2020	Class II	Colloidal Au NPs	Immunoassay reagent for qualitative detection of cTnI and myoglobin in serum or plasma
In vitro Nano-CheckTM AMI cTnI Cardiac Marker Test	Nano-Ditech Corporation	27/11/2020	Class II	Colloidal Au NPs	Immunoassay reagent for qualitative detection of cTnI in serum or plasma
In vitro Nano-CheckTM AMI 3 in 1 Cardiac Marker Test cTnI, CK-MB and Myoglobin	Nano-Ditech Corporation	08/12/2020	Class II	Colloidal Au NPs	Immunoassay reagent for qualitative detection of cTnI, CK-MB, and myoglobin in serum or plasma
Nanopia KL-6	Sekisui Medical, Ltd	21/08/2023	Class II	KL-6-coated latex NPs	Reagent for quantitative in vitro detection of sialylated carbohydrate antigen (KL-6) in serum or plasma
Nanopia TDM carbamazepine	Sekisui Medical, Ltd	03/04/2020	Class II	Carbamazepine-coated latex NPs	Reagent for quantitative in vitro detection of carbamazepine in serum or plasma
Nanopia TDM digoxin	Sekisui Medical, Ltd	18/05/2020	Class II	Digoxin-coated latex NPs	Reagent for quantitative in vitro detection of digoxin in serum or plasma
Nanopia TDM valproic acid	Sekisui Medical, Ltd	18/05/2020	Class II	Valproic Acid-coated latex NPs	Reagent for quantitative in vitro detection of valproic acid in serum or plasma
Nanopia CRP	Sekisui Medical, Ltd	17/04/2020	Class II	CRP-coated latex NPs	Reagent for quantitative in vitro detection of C-reactive protein in serum or plasma
Nanopia P-FDP	Sekisui Medical., Ltd	17/02/2020	Class II	P-FDP-coated latex NPs	Reagent for quantitative detection of fibrin/fibrinogen degradation products (FDP) in plasma or serum
Nanopia MMP-3	Sekisui Medical., Ltd	10/02/2020	Class II	MMP-3-coated latex NPs	Kit for quantitative detection of matrix metalloproteinase-3 (MMP-3) in serum or plasma
Nanopia D dimer	Sekisui Medical, Ltd	26/11/2021	Class II	D dimer-coated latex NPs	Kit for quantitative in vitro detection of D-dimer in serum or plasma
Series of LigaSure Sealer/divider, nano-coated systems	Covidien, Llc	27/06/2022, etc.*	Class II and III	Nano-coated surface	Surgical equipment for cutting and coagulating tissue, blood vessels, and lymphatic vessels in open surgeries
Grandio light-curing nano-hybrid filling material	Voco GmbH	03/08/2021	Class III	Nano-hybrid composite	Dental filling material for Class I-V cavities, primary teeth, core buildups, and resin inlays
Filtek Z250 XT Nano Hybrid Universal Restorative	3 M ESPE Dental Products	22/01/2020	Class III	Visible light-activated nanohybrid composite	Dental restorative material for direct restoration of teeth, core buildup, splinting, and indirect restorations like inlays, onlays, and veneers
Nano-technology dental adhesive	Dentsply Detrey GmbH	18/08/2020	Class III	Nanoscale light-cure filler	Dental adhesive for composite restorations, cavity lining, and protecting sensitive cervical areas
Adaptix^™^ Interbody System with Titan nanoLOCK^™^ Surface Technology	Medtronic Sofamor Danek USA, Inc	10/07/2023	Class III	Titanium nanoscale featured surface	Spinal fusion system for thoracolumbar fixation in DDD patients (L2-S1)

^*^Disc.: Due to the complex regulatory process and need for continuous improvement, medical devices often undergo multiple revisions and approvals, resulting in several decision dates throughout their life cycle

In the European Union (EU), over 70 EU-certified private notified bodies regulate medical device approvals, issuing Conformité Européenne (CE) marks for EU-wide marketing. Differently from the United States and China, national authorities oversee post-market safety [261]. As of June 2024, the European Database on Medical Devices (EUDAMED) lists 130 approved medical devices that either contain nanomaterials or have "nano" in their name [262]. The products among them that can be identified as NMDs through publicly available information are listed in Table 6. They are mainly intended for use in the following areas: dental restorative materials, cosmetic injections, laboratory instruments, magnetic NPs for anti-cancer purposes, protein assays, COVID-19 rapid diagnostic kits, and others. Within this assortment, products related to dentistry represent the largest category, constituting over 30% of the total. This category specifically includes light-cure nanocomposites and zirconia-silica nanocomposite fillers for dental restoration. In addition, one particular example is aimed at cancer therapy: NanoTherm^®^ AS1 is a water-based solution filled with superparamagnetic NPs, primarily made of Fe_3_O_4_ with a diameter of roughly 12 nm. These NPs function as energy transducers for magnetothermal therapy aimed at cancer treatment. To ensure the stability of these NPs within tumour tissues, their surface is coated with an aminosilane. In Europe, this formulation is applied in a dual treatment approach, combining magnetic hyperthermia therapy with radiation therapy, for patients suffering from recurrent glioblastoma multiforme (GBM) [263]. Another example, Proteonano™, represents instead a proteomics analysis technology that use magnetic NPs modified with peptides and small molecules for the identification of various protein biomarkers.

**Table 6:** List of NMDs approved for EU-wide marketing. Extracted from EUDAMED database https://ec.europa.eu/tools/eudamed/#/screen/home (Keyword: nano)

Device/trade name	Manufacturer/producer	Nomenclature code(s)	Risk class	Nanomaterial	Indication
Alergo Alnanogel	Alegro Medical GmbH	Medicated bandages, with zinc oxide and other components—other	Class I	Nanogel	The bandage is designed to provide superior protection against bacterial infection, while also offering relief from itching, burning, and other common symptoms associated with neuro dermatitis
JBP NanoLink Fille HA S	BioPlus, Ltd	Resorbable filling and reconstruction devices	Class III	Hyaluronic acid NPs	The gel is a transparent, viscous, and sterile substance in a pre-filled syringe for facial tissue augmentation by injection into the skin layer
Medental nanohybrid light cure composite	Conamco SA de CV dba Medental	Dental restoration compomers	Class IIa	light-cure nanocomposites	The dental composite for restorations of all cavity classes, Indirect inlays, onlays and veneers
Nano Synt.	Dentscare Ltda	Dental graft devices	Class III	Nano-hydroxyapatite	The synthetic bone replacement material is based on biphasic calcium phosphate with excellent osteoconductive action, being resorbed and replaced by living bone during bone remodelling
NanoTherm^®^ AS1	MagForce NT GmbH	Active-implantable devices—other	Class III	Superparamagnetic NPs	NPs for magnetothermal therapy in tumour treatment
Proteonano^™^ Whole Proteome Profiling Kit	Nanomics Biotechnology, Ltd	Immunochemistry reagents	Class A	Magnetic NPs	The kit uses modified magnetic NPs to compress the dynamic range of protein concentrations in complex biological samples, enhancing the detection of medium- and low-abundance proteins by reducing the dominance of high-abundance proteins
NanoSigma Oral Gel	NanoSigma Biotech,Ltd	Dressings for wounds, sores and ulcerations	Class I	Gel’s nanotechnology	The oral gel is a liquid bandage for oral ulcer and injured wound. It is suitable for oral mucosa wound
Saliva SARS-CoV-2 (2019-nCoV) Antigen Test Kit (Nanocarbon Assay)	Ningbo Beautiful Life Medical Biotechnology Development,Ltd	Rapid tests & point of care—immunochemisty—other	IVD General	Nanocarbon	The kit is a lateral flow, one-step immunoassay for the qualitative detection of N antigen in saliva sample
QNome-3841hexDx Nanopore Sequencer; QNome-3841 Nanopore Sequencer	Qitan Taike Biotechnology, Ltd	DNA synthesisers	Class A	Nano-sized protein pores	The instrument is used for determining the order, type and quantification of bases in DNA fragments
Znano	Zest Anchors, Llc	Dental conservation composites	Class IIa	zirconia-silica nanocomposite	The dental composite is a highly-filled, light cured, resin-based dental composite material

From Tables 1–6, it is evident that while the classification of NMDs varies across regions, all systems fundamentally rely on the risk level associated with the device to determine regulatory requirements. The United States, Europe, and China all use a tiered approach, classifying devices into different categories to align regulatory requirements with the device’s potential risk, as shown in Table 7. FDA and NMPA both categorise devices into three classes: Class I for low-risk devices that require minimal oversight, Class II for moderate-risk devices needing more regulatory controls (such as 510(k) premarket notification for FDA and enhanced supervision for NMPA), and Class III for high-risk devices that support or sustain life, requiring stringent evaluation and approval processes [264]. The EU employs a more detailed four-class system, with Class I for low-risk, Class IIa and IIb for moderate to higher-risk depending on usage duration and invasiveness, and Class III for high-risk devices [265]. This system emphasises device invasiveness and duration of use more than the FDA or NMPA. Despite these differences, all systems aim to ensure device safety and effectiveness by increasing regulatory requirements as the risk level rises, particularly for devices that are life-supporting or implanted. These frameworks reflect a global commitment to maintaining high standards in medical device safety, adapted to each region's regulatory environment.

**Table 7:** Comparison of medical device classification systems

Classification Level	United States	Europe	China
Class I	Low-risk devices (general controls)	Low-risk devices (non-invasive or short-term use)	Low-risk devices (Routine management)
Class IIa	–	Medium-risk devices (short-term or moderately long-term use)	–
Class II	Moderate-risk devices [special controls, require 510(k)]	–	Moderate-risk devices (Enhanced supervision required)
Class IIb	–	Higher-risk devices (medium to long-term implantable or invasive devices)	–
Class III	High-risk devices (require PMA premarket approval)	High-risk devices (used to sustain life or implantable)	High-risk devices (Vital for life, strict requirements)

The commercialisation decision for NMDs is heavily influenced by these regulatory classifications, as they dictate the level of evidence required for approval and market access. For example, devices intended for high-risk applications, such as implantable devices or those involving critical nanomaterial interactions with the human body, typically face stricter regulatory scrutiny and require more extensive clinical data. Understanding these regulatory frameworks is crucial for developers to strategise their product development and market entry effectively.

In addition, the divergent application focuses of nanotechnology in medical devices across the United States, Europe, and China reflect differences in the clinical translation and commercialisation of nanotechnology globally, which can be attributed to several factors, including differing healthcare priorities, regulatory environments, R&D capacities, and market demands in these regions. Different regions may prioritise healthcare needs based on prevalent diseases, demographic profiles, and public health challenges. For instance, the higher emphasis on orthopedics and spinal surgery in the United States could reflect a larger aging population suffering from musculoskeletal disorders [266]. The focus of Europe on dentistry might be due to a combination of high dental care standards and public health policies prioritising oral health [267]. In contrast, the high interest of China for diagnostics could stem from a strategic push to improve early disease detection and management in a populous country, aiming to reduce long-term healthcare costs [268]. In addition, the regulatory frameworks governing medical devices, including those using nanotechnology, vary significantly across regions, which can influence the type of products that reach the market [269]. Stricter or more lenient regulations in one area of healthcare over another can steer the R&D focus. Manufacturers with superior R&D capabilities can secure a larger market share by leveraging intellectual property protection, which limits similar technological advancements of their competitors, thus fostering differentiated competition. Market demands, driven by patient needs, insurance coverage, and societal values, also influence the focus of nanotechnology applications in medical devices. The economic incentives to address specific healthcare challenges can drive companies to innovate in areas with the highest return on investment.

The development and market introduction of nanomedicines and NMDs are ongoing, but the products available are still few compared to the extensive research indicated by 50,424 articles on 'nanomedicine' and 15,220 on 'nano medical device' found on PubMed (as of June 2024). This disparity underscores the significant hurdles in their clinical translation and commercialisation.

### Market entry challenges and cost-effectiveness

The commercialisation of nanomedicines and NMDs faces several significant challenges, particularly in terms of market entry barriers and cost-effectiveness. This section explores these obstacles in detail, focusing on the financial, manufacturing, and quality control issues that impact the successful introduction of nanotechnology in healthcare.

#### Regulatory and approval challenges

**Fig. 29:**
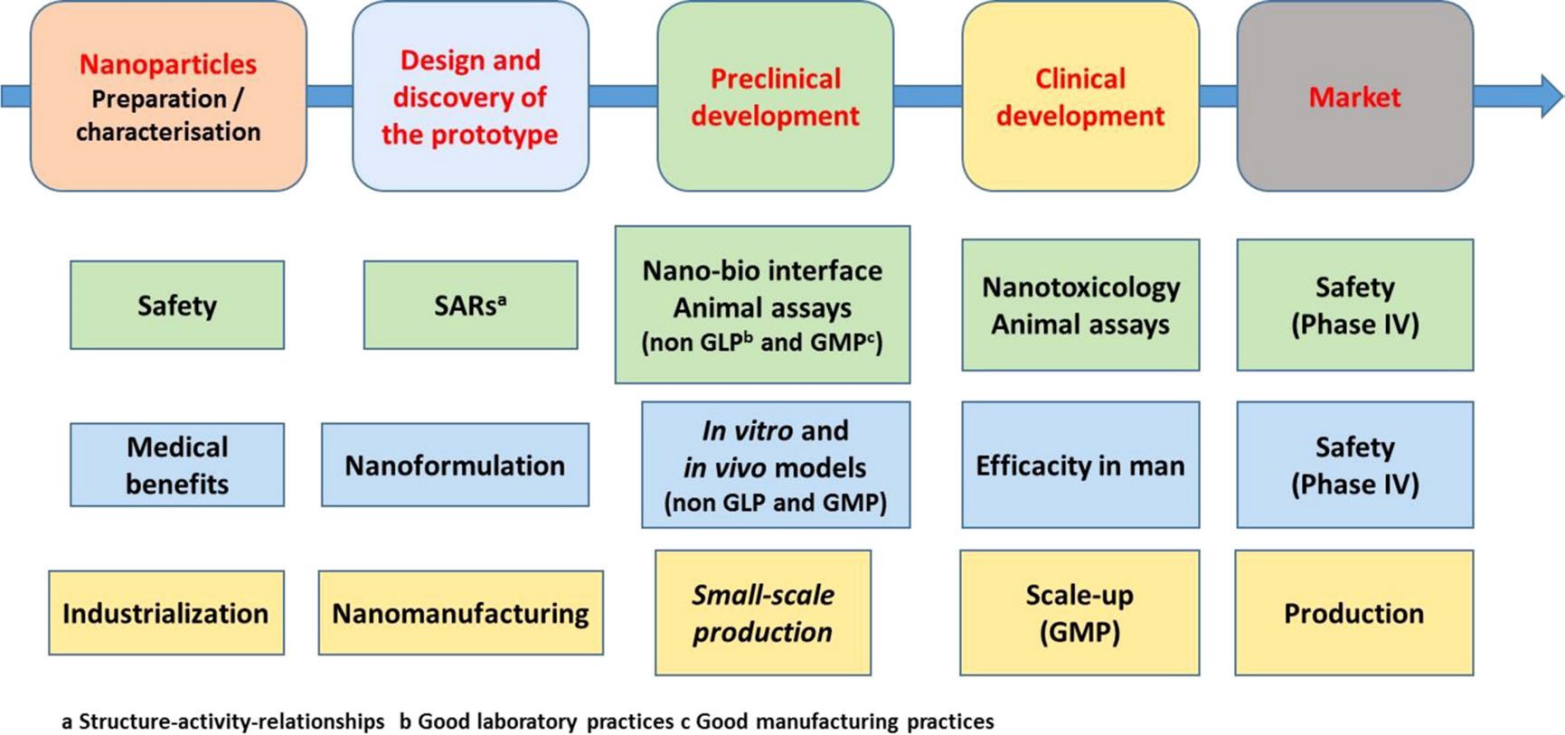
Summary of key steps for clinical application of NPs. Adapted from Webster [274]

One of the main challenges for bringing nanomedicines and NMDs to market is meeting the stringent regulatory requirements, which are crucial to ensuring the safety, efficacy, and quality of these products. Regulatory approval typically involves comprehensive preclinical and clinical evaluations, with toxicity testing and safety assessments being essential components [270]. Due to the unique size and surface characteristics of nanomaterials, they may exhibit toxicity profiles that differ from those of traditional materials, necessitating specific tests for acute, subacute, and chronic toxicity, as well as genotoxicity, immunotoxicity, and environmental toxicity to assess their potential impact on biological systems and the environment [271]. Sect. “Nanotoxicity and safety risks” will provide a more in-depth discussion on the health risks of nanomaterials in vivo and in the environment, as well as the mechanisms of toxicity. Additionally, safety evaluations generally include a range of in vitro and in vivo studies to understand the biocompatibility of nanomedicines and NMDs, as well as their absorption, distribution, metabolism, and excretion (ADME) properties in the body [272]. These studies must strictly adhere to Good Laboratory Practices (GLP) and Good Manufacturing Practices (GMP) to ensure the reliability of the tests and the consistency of the products [273]. Figure 29 illustrates the steps necessary to bring nanomaterials to healthcare market. While these rigorous requirements help reduce uncertainties in the approval process, they also significantly increase the cost and time required for research and development, posing a major challenge to market entry [270]. By meeting these complex regulatory requirements, nanomedicines and NMDs can successfully enter the market and provide safe and effective treatment options for patients.

Moreover, the requirements for these evaluations can vary significantly across different regions, creating additional obstacles for companies seeking to bring their products to the global market. Regulatory agencies in various countries have different standards and procedures for the approval of nanomedicines and NMDs. For example, as described in Table 7, different regions have distinct requirements for the classification of medical devices. These discrepancies mean that companies may need to conduct additional tests and modifications to meet the specific requirements of each market, thereby increasing both time and cost. This complexity necessitates that companies carefully consider the regulatory requirements and market characteristics of each region when developing global commercialisation strategies. Sect. “Current regulatory policies around the globe” will provide a detailed discussion on the current state of global regulations and offer recommendations for improvement, helping companies better navigate these challenges and achieve global market entry for nanomedicines and NMDs.

#### Financial barriers and R&D costs

Developing nanomedicines is an expensive and resource-intensive process that poses significant financial challenges for companies, particularly for smaller firms and start-ups [275]. The costs associated with R&D encompass a wide range of activities, including the design, synthesis, characterisation, and testing of nanomaterials. Each of these stages requires specialised equipment and expertise, which can drive up expenses. Additionally, scaling up production from laboratory-scale synthesis to manufacturing quantities suitable for clinical trials and eventual market supply involves substantial investment in infrastructure and technology.

Beyond the initial development costs, conducting extensive clinical trials to meet regulatory standards is another major financial hurdle [276]. Clinical trials, which are necessary to establish the safety, efficacy, and quality of nanomedicines, often involve multiple phases and require significant resources to manage and execute. These trials are not only costly due to the need for specialised staff, facilities, and materials but also time-consuming, which further delays the potential return on investment. Smaller companies and start-ups may find these expenses particularly prohibitive, as they typically lack the financial reserves and access to capital that larger pharmaceutical firms possess.

The financial burden of developing nanomedicines is further compounded by the uncertainty of return on investment. The innovative nature of nanomedicines means that there is often limited prior data to support their efficacy and safety, leading to a higher risk of delays or failures in the approval process [277]. This uncertainty can deter investors and make it difficult for companies to secure the necessary funding to carry their products through to market.

Given these substantial financial barriers, it is crucial for companies to carefully strategise their R&D investments and seek collaborative partnerships or alternative funding sources to mitigate risks and enhance their chances of successful market entry.

#### Manufacturing and quality control challenges

Scaling up the production of nanomedicines from laboratory research to commercial-scale manufacturing presents unique challenges due to the distinct properties of nanomaterials, such as their small size, high surface area, and reactivity [278]. These characteristics necessitate specialised manufacturing processes and equipment to maintain consistent quality. Even slight variations in particle size or surface properties can significantly impact the efficacy and safety of the final product.

Ensuring reproducibility across large-scale production is difficult, as processes optimised at a small scale may not perform identically when scaled up, potentially leading to variability. This requires strict control over production conditions and process parameters to ensure uniformity. Adherence to GMP guidelines is essential to meet regulatory standards, involving rigorous quality control measures, real-time monitoring, and validation of processes to detect and correct deviations [279].

Additionally, manufacturing nanomedicines requires advanced analytical techniques to accurately characterise nanomaterials, such as dynamic light scattering and electron microscopy, to ensure consistent particle size, morphology, and surface chemistry [280]. These requirements increase the complexity and cost of production but are crucial for ensuring product quality and safety.

#### Ethical implications

The integration of nanotechnology into healthcare brings forth several ethical considerations that must be addressed to ensure responsible and equitable use. One of the primary concerns is the issue of informed consent. Nanomedicines and NMDs often involve complex mechanisms and potential risks that may not be fully understood by patients. The novelty and intricacies of these technologies can make it challenging for patients to comprehend the full scope of benefits, risks, and long-term implications associated with their use. This can lead to difficulties in achieving truly informed consent, as patients may not have all the necessary information to make well-informed decisions about their treatments [281]. Therefore, healthcare providers have a duty to develop comprehensive educational materials and employ effective communication strategies that clearly explain the nature and implications of nanotechnology-based interventions in a way that is understandable to patients, thus safeguarding patient autonomy.

In addition to informed consent, ethical considerations also extend to issues of privacy and equitable access. Nano-enabled diagnostics and treatments could collect extensive biological and genetic data, raising concerns about data security, privacy breaches, and the potential misuse of sensitive information [282]. This data, if inadequately protected, could be exploited by third parties such as insurance companies or employers, leading to discrimination or stigmatisation based on genetic predispositions [283]. Furthermore, the high cost of developing and manufacturing nanotechnology-based healthcare products can create barriers to access, particularly for disadvantaged populations or those in low-income countries [284]. The unequal distribution of these advanced technologies risks widening existing healthcare disparities. To address these ethical challenges, it is crucial to implement robust data protection regulations and develop policies that promote equitable access to nanotechnology-based innovations, ensuring that all patients, regardless of their socioeconomic status, can benefit from these advancements in healthcare.

#### Cost-effectiveness consideration

The cost-effectiveness of nanomedicines and NMDs is a critical consideration in their development and market adoption. Although these technologies offer significant clinical advantages, such as improved therapeutic outcomes, targeted delivery, and enhanced diagnostic capabilities, they also involve substantial costs. The high initial investments required for the specialised synthesis of nanomaterials and the advanced technologies needed for their production contribute to increased development expenses compared to traditional pharmaceuticals and medical devices. Additionally, both nanomedicines and NMDs require extensive preclinical and clinical testing to meet stringent regulatory standards, further adding to the financial burden. However, these high costs can be offset by the potential long-term benefits, such as enhanced drug delivery, reduced side effects, fewer invasive procedures, and shorter recovery times, which can ultimately lead to lower overall healthcare costs. Moreover, implementing policies that promote equitable access and affordability, as mentioned previously, can enhance the cost-effectiveness of these technologies by ensuring that a wider patient population benefits from their advantages. Comprehensive economic evaluations that account for both direct and indirect costs are essential to determine the value these technologies bring to healthcare systems. By balancing these costs against the potential benefits, nanomedicines and NMDs can be seen as viable options that not only improve patient outcomes but also contribute to economic sustainability in healthcare.

## Nanotoxicity and safety risks

The significant potential benefits of using nanotechnology in healthcare are undoubtedly appealing. However, the issue of biocompatibility must be addressed when any foreign substance is introduced into the human body for medical reasons, along with the environmental effects associated with commercialisation and market entry. Conducting a comprehensive and reliable evaluation of their toxicity throughout their lifecycle poses a challenge for professionals and organisations alike. This is because the reduction in size or dimensions, particularly to nanoscale in one or two dimensions, can lead to not only novel properties but also potentially unconventional toxicity behaviour.

**Fig. 30:**
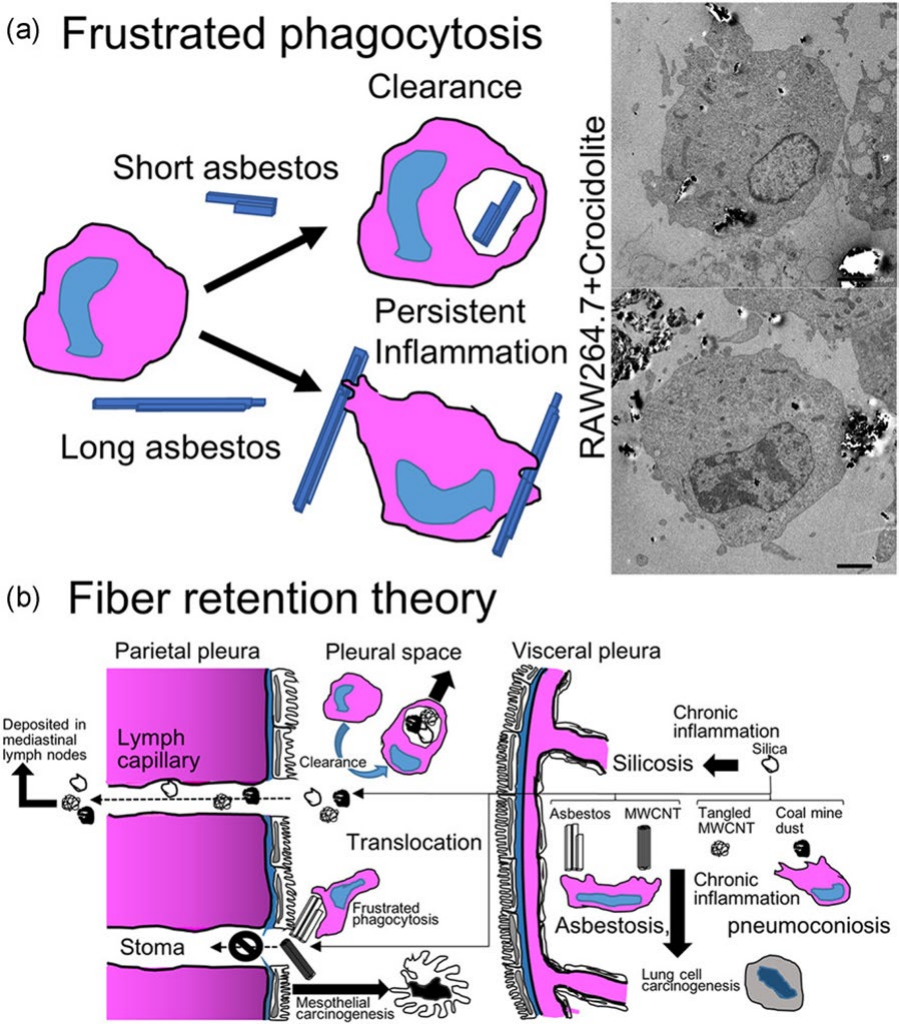
Mechanism of Biopersistent Fiber-Induced Carcinogenesis. **a** Short asbestos fibers are cleared by macrophages, while long fibers (15–20 μm) cause persistent inflammation due to incomplete phagocytosis. TEM images show crocidolite phagocytosis by RAW264.7 cells. **b** Long fibers translocate to the pleural space, triggering frustrated phagocytosis at stoma openings and releasing proinflammatory cytokines and genotoxic activators, leading to mesothelial carcinogenesis. Small particles like coal dust, silica, and tangled multiwalled carbon nanotubes (MWCNTs) are cleared by phagocytes or lymphatic drainage. Retained long fibers cause chronic inflammation, leading to asbestosis or pulmonary carcinoma. Tangled MWCNTs and coal dust also cause lung carcinogenesis through persistent inflammation. Adapted with permission from Zhang et al*.* [285]

Research has demonstrated that certain medical conditions may result from the improper exposure to nanomaterials. Mesothelioma, a type of cancer that forms in the protective lining of various internal organs, serves as a notable example, often linked to asbestos exposure. Asbestos, a collection of minerals composed of tiny, fibrous particles, was extensively used in the construction industry in the 1970s and shares similarities in size with nanomaterials. These tiny asbestos fibres can be inhaled and embedded in the lung tissue, causing damage over prolonged periods (Fig. 30).

Although over 60 countries worldwide have enacted bans on asbestos [286], there remains a valid concern that other substances with similar sizes and shapes, such as nanofibers, could pose similar health risks if they are introduced into the human body in a comparable manner. NPs have been shown to be inhaled and deposited by diffusion in the small-diameter tubes of the lower part of the respiratory tract and in the alveoli [287]. Furthermore, epidemiological research indicates that exposure to NPs is linked to an increased risk of cardiovascular and respiratory diseases [288]. Regrettably, advancements in research on this subject have been limited; more specifically, there has not been a consensus on a standardised methodology for toxicological testing and analysis among medical professionals and toxicologists. This challenge arises partly due to the need for a multidisciplinary approach that encompasses materials science, chemistry, physics, toxicology, and environmental science.

While human health risk assessment focuses on determining the acceptability of health risks from medical products to humans under specific exposure scenarios [289], the life cycle assessment of medical products evaluates and compares the environmental impacts of the products or processes throughout their life cycle, encompassing their potential (negative) impacts on human health among other effects [290]. Currently, data on the impact of nanomaterials on aquatic life and plants are limited, with a shortage of suitable research methods [291]. Despite variations in their objectives, processes, and scope, both types of risk assessments depend on similar data for insights into nanomaterial toxicity [290]. There is a consensus on the importance of assessing the ecological and environmental safety of nanomaterials, which necessitates the development of suitable models and new methods. The following sections contain a discussion on the safety risks of nanomaterials from both health and environmental perspectives.

### In vivo health risks

Typically, the body manages foreign chemicals through processes including exposure, absorption, distribution, metabolism, and excretion. These processes can be analysed through toxico-kinetics and a risk-based approach. Using logical and scientific rationale, along with ISO 10993, should elucidate the in vivo biosafety risk of a medical product. Yet, small particles are more easily taken up by cells, cross epithelial and endothelial membranes, undergo transcytosis, enter the blood and lymphatic circulatory systems, and ultimately accumulate in sensitive organs like the bone marrow, lymph nodes, spleen, and heart, therefore exhibiting significantly different dose sensitivities and kinetics [292]. Table 8 provides a comprehensive list of the primary and secondary biological barriers that NPs can penetrate, along with the organs they may accumulate in, offering an initial overview of the potential fate of NPs upon exposure to humans through different pathways.

**Table 8:** Potential safety risk factors of nanotechnology in healthcare applications

Potential safety risk factors in healthcare nanotechnology applications	
Penetration through each physiological barrier and cause of complex dose-dependence toxicology; Primary biological barriers Intestinal barrier [293, 294] pulmonary barrier [295] skin barriers and etc. [296, 297] Secondary biological barriers vascular endothelial barrier [298, 299] blood–brain barrier [300–303] blood-testis barrier [304, 305] placental barrier [306, 307]	Retention in each organ and cause of immunogenicity and/or toxicity; Liver [308] Lung [309–311] Spleen [312–314] Kidney [315, 316] Heart [317–319] Brain [320, 321]

Specifically for the respiratory system, a predictive model has been developed to detail the behaviour of NPs within it [322]. The model suggests that smaller particles are more prone to settle in the respiratory tract, with their distribution across various regions being size-dependent. NPs measuring 5 nm are equally likely to settle in the nasopharyngeal (upper airways), tracheobronchial, and alveolar areas. However, for particles smaller than 5 nm, there is a higher tendency for them to settle in the nasopharyngeal area, while those larger than 5 nm predominantly settle in the alveolar region. This variance in settlement locations is likely to affect the biological interactions of the NPs. This model, however, exhibits limitations in two main areas: firstly, it was designed specifically for spherical NPs, leaving uncertainty about its applicability to NPs of different shapes; secondly, it uses default material behaviours primarily derived from studies on thorium, uranium, or technetium, despite the fact that the behaviour of a nanoparticle is highly influenced by its chemical composition and surface properties [287]. To add even more complexity to an already difficult challenge, the final destinations of NPs are diverse. To provide an example, after administering 20 nm colloidal Au NPs to mice through intratracheal instillation, these particles were found not only at the basal membrane between alveolar and endothelial cells but also on the surface of blood vessel endothelial cells, with a small fraction even detected in the bloodstream [323]. Extensive research has confirmed that NPs can cross biological barriers, impacting the parenchymal components of organs either toxically or therapeutically, as detailed in prior reviews [324–327].

These findings lead to two key agreements: firstly, the toxicity of metallic NPs cannot be inferred from the existing toxicology of their bulk metal counterparts [328], and secondly, once inside an organism, NPs can distribute across tissues in ways not governed by known physiological processes. Consequently, there is no clear guidance for nano-toxicology evaluation using traditional toxicological approaches, primarily due to the intricate interactions between nanomedicines and biological systems [329]. To advance in this area, it is crucial to understand the mechanisms behind the complex reactions to nanomaterial exposure. This section outlines common mechanisms of action, as listed in Table 9.

**Table 9:** Potential safety risk factors of nanotechnology in healthcare applications

Presently known mechanisms of nanotechnology potential risks	
The complex dose-dependent toxicity linked to nanomaterials, which includes unintentional translocation or retention, can manifest through the following mechanisms: Transcellular/paracellular transport Physical damage to cell membrane	Toxic phenomena related to nanomaterials, including lipid peroxidation, protein alterations, DNA damage, interference with signaling functions, and modulation of gene transcription, may occur through the following mechanism: Induction of oxidative stress

#### Transcellular transport mechanisms

When nanomaterials enter a biological system, they inevitably engage with a variety of biological components, such as DNA, proteins, membranes, cells, and organelles, based on the specific context. The surfaces of these nanomaterials are quickly coated by biomolecules from the biological environment, with proteins being the most significant, forming what is known as a “protein corona.” This corona bestows a biological identity on the nanomaterials, influencing their subsequent interactions within the biological environment. Notably, the makeup of the “protein corona” is subject to change, determined by the concentrations and affinities of its different protein constituents to the nanomaterial surface [330]. Simultaneously, a key characteristic of nanomaterials is their relatively high surface-area-to-mass ratio, enabling them to adsorb significant quantities of materials per unit of mass. This capacity to bind a wide array of substances is thought to significantly influence their behaviour within cells under certain conditions. For instance, an in vitro investigation of polystyrene NPs demonstrated that NPs with a positive charge could penetrate cells 20 to 40 times more rapidly than those with a negative charge [331]. For NPs administered intravenously, their distribution across different organs is ultimately influenced by the plasma proteins that adhere to their surfaces. The distinct physical and chemical characteristics of these NPs—such as their size, surface area, modifications, charge, hydrophobic/hydrophilic properties, and redox activity—along with the in vivo microenvironment, dictate their varying patterns of interaction with proteins and lipids across various organ tissues and cells. This interaction determines the eventual location and destiny of the NPs within the body [332].

**Fig. 31:**
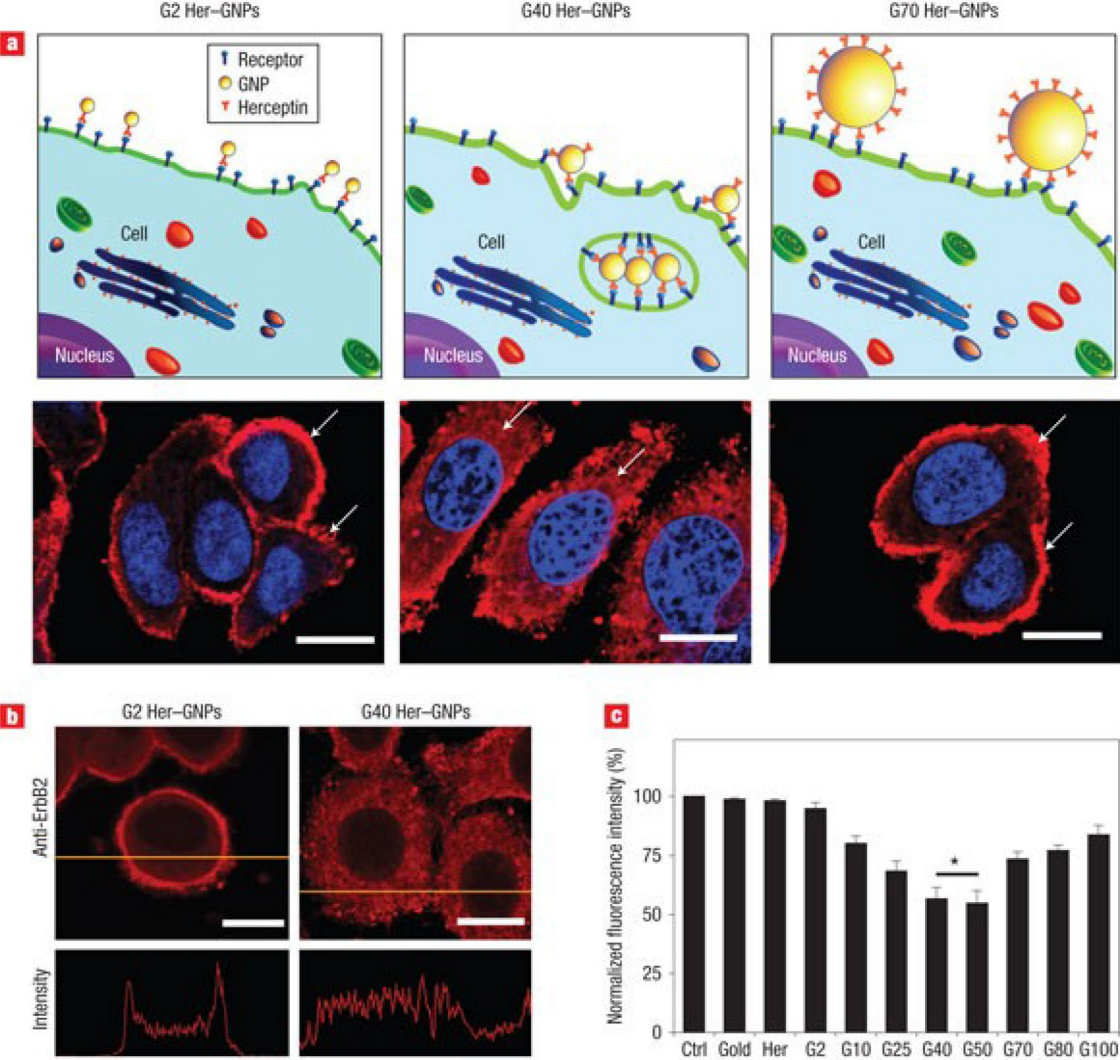
**a** Illustrations with corresponding fluorescence images of ErbB2 receptor localisation after treatment with different-sized Herceptin–colloidal gold nanoparticles complexes (Her–GNPs). Arrows indicate ErbB2 receptors, and the nucleus is counterstained with DAPI (blue) (scale bars = 10 µm). **b** Cross-sectional fluorescence intensity measurements of ErbB2 receptor localisation patterns with G2 (2 nm) and G40 (40 nm) Her–GNPs (scale bars = 10 µm). **c** Surface ErbB2 expression analysis using untreated cells normalised as 100% expression level (Ctrl). Cells were treated with unmodified 40-nm GNPs (Gold), Herceptin (Her) and Herceptin-modified GNPs of various sizes (* denotes statistical significance for G40/G50 compared to Her–GNPs of other sizes, p < 0.05, ANOVA). Error bars, ± s.d.; n = 4. Adapted with permission from Jiang et al*.* [335]

Research on inhaled nanomaterials has shown that while cells can engulf particles of most sizes, those smaller than 100 nm can move across alveolar epithelial cells via diffusion through the cell membrane lipid bilayer [333] or, in certain cases, cross the air-blood barrier through gaps between cells [334]. Experiments involving Au NPs and Ag NPs, coated with antibodies and of various sizes, demonstrated their role in the process of membrane receptor internalisation [335]. The binding and activation of these membrane receptors, along with the resulting protein expression, were notably influenced by the size of the NPs. NPs within the 2 to 100 nm range influenced signal pathways critical for cell function, with those measuring 7, 40, and 50 nm showing the most pronounced effects (Fig. 31). A summary of size-dependent cellular uptake of nanomaterials in regarding to the examined cell lines and major conclusions is presented in Table 10.

**Table 10:** Size-dependent active and passive cellular uptake of nanomaterials with examined cell lines, and major conclusions. Adapted with permission from Kumar et al*.* [336]

Nanomaterial	Size	Cell line	Major outcomes
Au NPs	2–15 nm	MCF-7	Higher uptake of smaller NPs; 2/6 nm located in cytoplasm and nucleus,15 nm only in cytoplasm
Aqueous QDs	2–7 nm	A-427	Size-dependent internalisation efficiency
Au NPs	2.4–89 nm	Cos1	2.4 nm: in nucleus; 5.5 and 8.2 nm: partially in cytoplasm; 16 nm and above: no uptake
TiO_2_	5–80 nm	A549	Uptake depends on overall size (with hard corona)
Au NPs	13 nm, 45 nm	CF-31	45 nm: clathrin-mediated endocytosis 13 nm: mostly phagocytosis
Polystyrene NPs	40 ~ 2000 nm	HeLa, A549, 1321 N1, HCMEC D3, RAW 264.7	Uptake highly size-dependent for all cell lines; larger NPs enter more slowly
DPA-Quantum dot	8 nm	RBCs	QDs penetrate cell membranes without pore formation
Mesoporous silica	100 ~ 600 nm	RBCs	Strongly dependent on surface chemistry and nanoparticle size

#### Paracellular transport mechanism

By interfering with specific transmembrane proteins, NPs can regulate internalisation and other fundamental cell functions, and also penetrate biological barriers. This is achieved by inducing the degradation, conformational change, and disintegration of proteins that link adjacent cells, facilitating paracellular transport. There are various types of intercellular junctions, including tight junctions (TJs), adherent junctions (AJs), desmosomes, and gap junctions (GJs) [337]. Although they serve different functions, desmosomes provide strong linkages that maintain stable adhesion between cells exposed to mechanical stress, such as those in the bladder and myocardium [338]; GJs are primarily used as channels for the exchange of ions and signal molecules between neighbouring cells, playing a crucial role in intercellular communication [339]. Regarding the function of paracellular permeability, which is an essential component in sealing the paracellular route in epithelial and certain endothelial barriers (e.g., blood–brain barrier), the structure of TJs is deemed responsible. They “glue” together portions of neighbouring cell membranes adjacent to the apical side, preventing lipids and specific proteins from mixing between the apical and basal sides of the cell, and limiting the space between cells to a very narrow range (from about 5 Å to 50 Å in different tissues) [340].

**Fig. 32:**
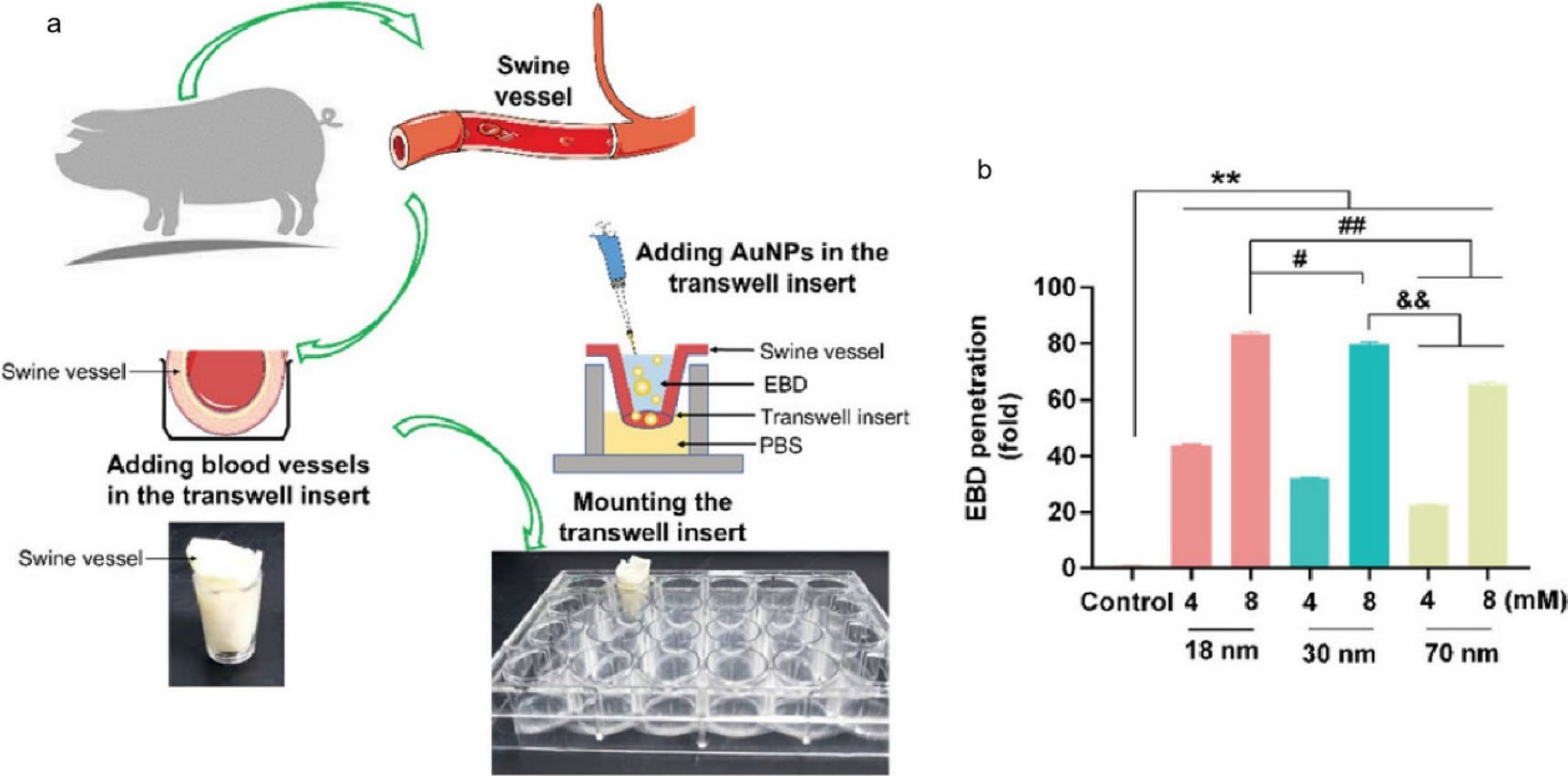
Au NPs-Induced Endothelial Leakiness in Swine Vessels. **a** Ex vivo construct scheme. **b** Size-dependent decrease in endothelial leakiness, measured by Evans blue dye (EBD) penetration. Smaller Au NPs (18 nm) at 8 × 10⁻^3^ m showed greater leakiness compared to 30 and 70 nm Au NPs. Results are means ± SD (n = 3), analysed by one-way ANOVA for 18, 30, and 70 nm Au NPs at 4 and 8 × 10⁻^3^ m concentrations. #P < 0.01, **, ##, &&P < 0.001. Adapted with permission from Lee et al*.* [341]

A detailed study investigated the impact of Au NPs size on their interactions with the paracellular AJs (Fig. 32) [341].

It was found that Au NPs with diameters of 18 nm and 30 nm were sufficiently small to migrate into the AJs and disrupt vascular endothelial (VE)-cadherin homophilic interactions, whereas Au NPs measuring 70 nm in diameter could not cause significant disruption. Another study investigated the impact of pristine graphene on brain microvascular endothelial cells (BMVECs), revealing a reduction in the expression of the TJ protein occludin, increased permeability, and a resulting increase in BMVEC cell death [342]. Additionally, a significant drop in mitochondrial membrane potential was observed in rats exposed to Ag NPs, leading to a decrease in adenosine triphosphate (ATP) levels and setting the stage for the onset of cell autophagy [343]. The study also revealed that autophagy is triggered in the brains of rats exposed to low doses of Ag NPs, unlike those exposed to silver citrate, highlighting how differences in surface chemistry can lead to distinct toxicity profiles.

The interaction between nanomaterials and proteins in the human body is particularly complex, especially since nanomaterials covered with proteins that trigger macrophage phagocytosis are identified by immune system cells with specific receptors. These interactions are not fixed and vary with the changing protein environment within the body, where abundant proteins may be gradually replaced by less abundant ones that have a higher affinity for the nanomaterials, indicating a dynamic process. The increasingly understood mechanism of interaction between nanomaterials and proteins at the molecular level has been illustrated in a recent publication [344].

#### Physical interaction

Surface chemistry may result in varied nanotoxicity behaviour via diverse biochemical interactions [345]. However, as the physical attributes of nanomaterials, such as shape and size, are scaled down, their significance becomes increasingly non-negligible in comparison to surface chemistry.

A study by Yuan et al*.* examined how diameter influences the uptake of NPs with a consistent surface ligand density through mathematical modeling [346]. The authors identified the most effective diameters for endocytosis as being approximately 30–50 nm. However, as each cell type has its distinct phenotype, the most effective size and distribution for NPs uptake should be determined individually, based on the specific cell type under examination and the surface chemistry involved. In another study published by Akcan et al*.*, a series of toxicity behaviours of Au NPs were detailed [347]: 50 nm particles showed maximum uptake by HeLa cells; particles ranging from 45 to 50 nm activated membrane receptors in SK-BR-3 cells; those between 15 and 50 nm had the capacity to cross the blood–brain barrier in mice; and 15 nm particles displayed the most extensive distribution across various organs (in mice), including blood, liver, lungs, spleen, kidneys, brain, heart, and stomach. Inside the cell, size remains significant. Williams et al*.* reported that the entry of cadmium telluride quantum dots (CdTe QDs) into various subcellular organelles varies by size and cell type: QDs smaller than 2.1 nm penetrate the nucleus, while those measuring 4.4 nm localise within the cytoplasm [348].

**Fig. 33:**
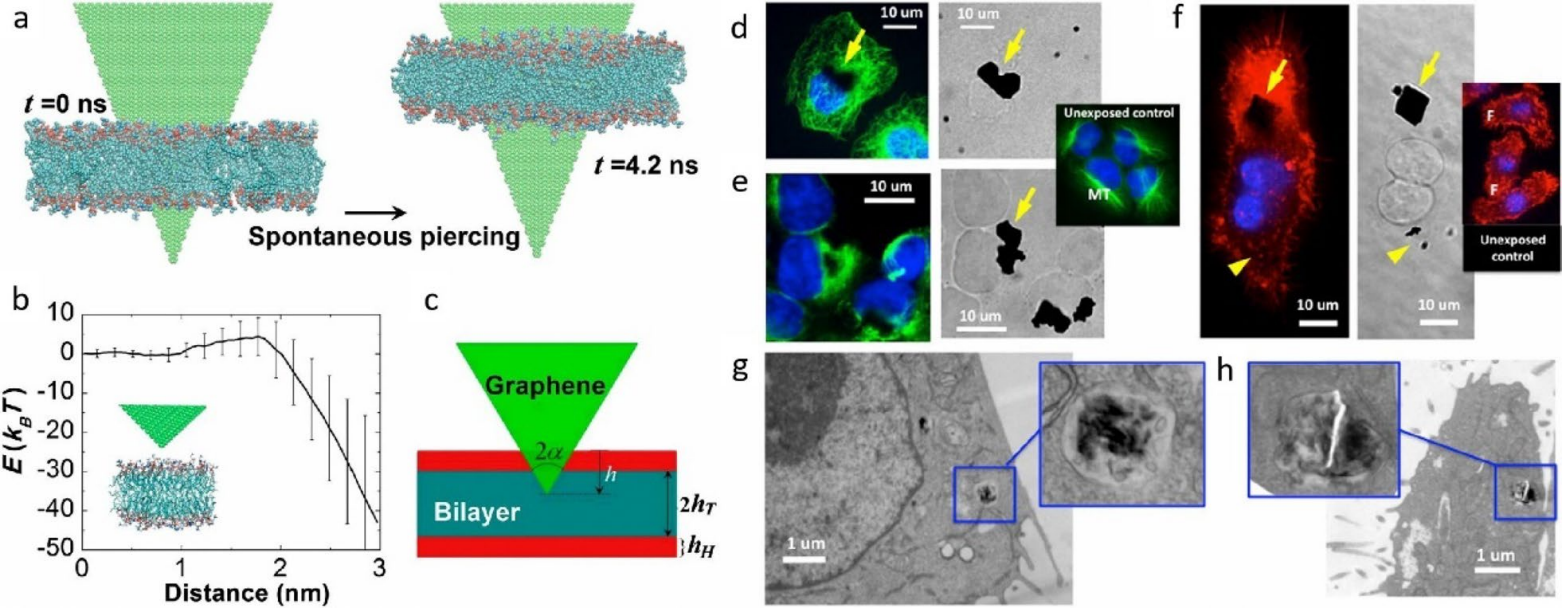
All-atom molecular dynamics simulations of corner piercing of a monolayer graphene across a lipid bilayer: **a** Spontaneous corner piercing observed. **b** Graphene–bilayer interaction energy vs. penetration distance, indicating a ~ 5kBT energy barrier; mean values from 11 simulations, error bars show SD. **c** Analytical model of corner piercing. Cellular uptake and internalisation of few-layer graphene microsheets: **d–f** Confocal images of human lung epithelial cells (**d**, **e**) and mouse macrophages (**f**) exposed to graphene microsheets (0.5–25 μm lateral dimension) after 24 h and 5 h, respectively. Nuclei (blue, DAPI), lung epithelial microtubules (green, FITC), and macrophage actin (red, rhodamine–phalloidin). Internalised graphene flakes (yellow arrows) disrupt cellular structures. **g**, **h** Transmission electron micrographs show graphene localisation in membrane-bound vacuoles in macrophages and lung epithelial cells after exposure to 10 ppm FLG sheets for 5 h and 24 h, respectively. Adapted with permission from Li et al*.* [349]

Beyond the common size parameter for describing uniform nanomaterials (typically NPs), an important study published by Li et al*.* has showed that ultrathin 2D synthetic materials like graphene, ranging from 0.5 to 10 μm in lateral size, could be fully internalised by cells after initially attaching by their edges [349] (Fig. 33).

The study provided an energy barrier-based explanation, indicating that for small graphene pieces, thermal motion and entropic forces near the cell membrane initially align the piece with the membrane plane, leading to spontaneous piercing by corners with a minimal energy barrier (about K_B_T level). It was noted that in the absence of sharp edges, cell membranes present a significant energy barrier to the penetration by long graphene edges, even if they are only atomically thin. This highlights that local sharp features can facilitate penetration when the activation barriers are surpassed, regardless of whether the overall dimensions are in the nanometer or micrometer scale.

#### Oxidative stress and high reactivity

As the size of NPs decreases, the proportion of surface atoms to the total number of atoms in the NPs significantly increases, enhancing the surface capacity and surface tension of the particles. This leads to alterations in the physicochemical properties of the NPs, a phenomenon known as the *surface effect* or *interface effect*. When the surface atoms are exposed, they lack surrounding atoms, possess numerous unpaired bond forces, and are more likely to react with other atoms to reach a stable state, thus exhibiting high chemical reactivity. The substantial specific surface area of NPs increases the number of surface atoms, raises the level of structural disorder, and results in mismatched bonding states, creating many active sites.

Specifically, in the context of copper nanoparticles (Cu NPs) [350, 351], when these particles enter the digestive system, the acidic gastric fluid environment (with a pH of about 2) triggers the rapid conversion of ultra-reactive nano-metallic particles into their ionic form. Cu NPs transform hydrogen ions more swiftly than their micron-sized counterparts, leading to an excessive accumulation of Cu ions in the body and consequent toxicity. This toxicity escalates as the size of the Cu particles decreases, with significant toxicity observed at 17 μm for micron-sized copper particles and at 23.5 nm for Cu NPs. While inert micron-sized Cu only causes intestinal blockage at extremely high doses (5000 mg/kg), in gastric fluids, the highly reactive Cu NPs quickly convert to ionic form, rapidly relocating to the liver and kidneys for metabolic processing and elimination. The swift generation of numerous Cu ions by the NPs induces Cu ions overload in the body, resulting in liver and kidney damage and the disruption of tissue function.

The primary internal and biological toxic effects from nanomaterials are largely due to their ability to generate an overabundance of ROS such as hydroxyl radicals, singlet oxygen, hydrogen peroxide, and superoxide anion radicals [352–354] These free radical species including ROS and RNS, are highly reactive because of their unpaired electrons, leading them to interact with different cellular and subcellular structures and resulting in damage to cell membranes, lysosomes, DNA, and mitochondria, ultimately causing cell death through apoptosis and necrosis. The excessive generation of these radicals disrupts normal cellular signaling and mitogenic responses, impairing cell function [355, 356]

Oxidative stress is a critical factor in primary indirect genotoxicity, primarily due to the action of free radicals. These reactive species can interact with cellular biomolecules, including DNA, leading to significant cellular damage. ROS are particularly harmful as they can oxidize purines and pyrimidines in DNA, as well as cause strand breaks. Although cells have repair mechanisms to address this damage, these processes are not always successful, potentially resulting in gene mutations or extensive chromosomal abnormalities. Moreover, nanomaterials can induce DNA damage through additional mechanisms. They may directly interact with DNA or interfere with the processes of DNA replication and cell division. Nanomaterials can also affect protein kinases, which are crucial for regulating key cell cycle events, such as DNA replication and cell division, thereby further contributing to genomic instability [357].

Ag [356], silica [358, 359], amorphous silica [360, 361], Au [362], and ZnO [363] nanomaterials have been identified as causing human cell toxicity by both increasing ROS production and damaging DNA. Similarly, carbon-based nanomaterials like C_60_ fullerene [364] have been noted for their potential to generate free radicals. C_60_ and single-walled CNTs can become photoactivated, leading to intersystem crossing (ISC), which generates free electrons and subsequently results in ROS formation.

Moreover, free radicals can trigger an inflammatory response by accumulating in alveolar macrophages and inducing significant inflammation. If not addressed, this inflammation can lead to various pulmonary conditions, including lung cancer.

#### Toxicity of typical nanomaterials

From the standpoint of drugs and medical devices, potential safety concerns typically fall into four main categories: (i) systemic toxicity, which is especially relevant for long-term exposure in the case of permanent implants, (ii) local toxicity, (iii) carcinogenicity, and (iv) special considerations, which depend on the device's application, such as blood contact or interactions with the central nervous system. The introduction of nanomaterials complicates the evaluation of each of these safety issues. Therefore, alongside fundamental research into the interactions between these novel materials and biological systems, this review provides a concise examination of the safety concerns associated with extensively studied nanotechnology materials to enhance understanding.

##### Systemic toxicity

Systemic toxicity of dispersive nanomaterials, whether they are manufactured or naturally occurring, is heavily influenced by their biokinetics. This is typically evaluated using the ADME(T) framework, where ‘A’ represents administration, ‘D’ denotes distribution, ‘M’ stands for metabolism, ‘E’ signifies elimination, and ‘T’ refers to toxicity [365]. Understanding the biodistribution and clearance rates of nanomaterials is particularly complex and challenging, which has significantly impeded the broader clinical application of nanomedicines and nanotechnology-based devices.

Joël Bourquin’s review examined the long-term biological fate of various clinically relevant nanomaterials, including liposomes, micelles, Au NPs, SPIONs, silica, and polymers [366]. The review identified the kidneys, testes, liver, and spleen as primary target organs for dispersive nanomaterials. For Au NPs, the study found that their distribution and retention in these organs were influenced by factors beyond particle size, such as surface properties and coating materials. As shown in Table 11, Au NPs of different sizes (ranging from 2 to 110 nm) demonstrated similar patterns of organ accumulation and variable clearance times, indicating that size alone does not reliably predict their behaviour in biological systems. This data highlights that Au NPs exhibit consistent biodistribution across various sizes, with accumulation in several organs regardless of particle dimensions. Additionally, the clearance times for these NPs are not directly correlated with size, suggesting that other factors, including surface chemistry and shape, have a significant impact. The importance of these factors emphasises that a comprehensive understanding of NPs behaviour requires considering properties beyond size, such as surface characteristics and coatings, to accurately predict their biological interactions and clearance.

**Table 11:** Long-term biodistribution data of gold-based nanomaterials based on collected evidence. Adapted with permission from Bourquin et al*.* [366]

Material	Size (nm)	Animal	Primary organs with accumulation	Clearance observed (days)	Comments on biodistribution
Ethanediamine-coated Au NPs	3.2	Mouse	Kidney, Testis, Liver, Lung, Heart, Spleen	90	Detected in multiple organs, size not a clear predictor
Ethanedioic-acid-coated Au NPs	3.7	Mouse	Liver, Spleen, Lung, Heart, Brain	90	Similar to 3.2 nm, broad distribution
Au NPs	2 ± 0.5	Mouse	Liver, Spleen, Lung, Heart, Brain	90	Consistent with 3.2 and 3.7 nm, suggests size-independent distribution
Au NPs	5 ± 1	Mouse	Liver, Spleen, Lung, Heart, Brain	90	Distribution similar across size range
Au NPs	10 ± 2	Mouse	Liver, Spleen, Lung, Heart, Brain	90	Broad organ distribution regardless of size increase
CALNN-coated Au NPs	16	Rat	Thymus, Heart, Liver, Kidney, Brain, Spleen, Testis, Bladder	28	Rapid clearance, diverse organ distribution
Citrate-coated Au NPs	20	Rat	Liver, Spleen, Kidney, Brain, Bone Marrow	60	Increased size but similar clearance time
Au nanorods	55 × 13	Rat	Femur, Muscle, Liver, Heart, Kidney, Spleen, Lung	28	Larger dimensions, yet cleared similarly to smaller NPs
PEGylated Au NPs containing TNF-α	27	Mouse	Spleen, Liver, Kidney	120	Longer retention, not purely size-dependent
PEG stabilised silica coated Au NPs	110	Mouse	Bladder, Liver, Lung, Spleen, Brain	28	Large size, rapid clearance contradictory to expectations

High surface activity can lead to aggregation, which makes nanomaterials more easily recognised and phagocytised by macrophages, particularly in blood-rich organs such as the liver and spleen. This recognition and capture process significantly impacts the biodistribution and retention of NPs in the body. Furthermore, surface chemistry plays a vital role in NPs behaviour; certain surface characteristics may cause NPs to cluster, resulting in increased accumulation in organs like the kidneys and complicating their excretion. Additionally, variations in surface charge can affect how NPs interact with and are internalised by tissue cells, further influencing their biodistribution. These findings underscore that understanding the biological fate of NPs requires a comprehensive analysis of surface properties, as these factors are critical determinants of their interactions within biological systems and their eventual clearance from the body.

In addition to size and surface properties, the shape of nanomaterials is a critical factor in evaluating their toxic properties. Carbon-based nanomaterials, such as graphene oxide (GO), exemplify how different shapes—such as sheets, flakes, and nanoribbons—can affect the rate and extent of internalisation and interactions with biological structures. For instance, irregularly shaped GO sheets are cleared more slowly, leading to prolonged exposure and increased toxicity, whereas spherical GO NPs are cleared more rapidly, reducing long-term toxicity [367].

Ultimately, the systemic toxicity of nanotechnology-based drugs and devices depends on a comprehensive understanding of biodistribution and clearance processes. This understanding requires not only a detailed study of the size, shape, and physicochemical properties of nanomaterials but also consideration of how these materials are applied in the human body. Manufacturers must also consider the potential generation of nanoscale debris during long-term use, which could impact safety and efficacy.

##### Local toxicity

Local toxicity of nanomaterials is often associated with their route of administration, such as topical application or the site of implantation. Research has shown that Ag NPs, for example, can induce local toxicity at the point of entry, triggering an inflammatory response from the immune system [368, 369]. When nanomaterials interact with local tissues, they can provoke inflammation and lead to tissue damage. For instance, prolonged exposure to long asbestos fibers can result in fibrosis, characterised by the accumulation of excess fibrous connective tissue, which can impair organ function and lead to disease [370]. This local toxicity can arise from the intrinsic properties of the materials themselves or from their interactions with biological molecules, leading to chronic inflammation. A notable example is breast implant-associated anaplastic large cell lymphoma (BIA-ALCL), which has been identified in patients with various types of textured implants, used for both breast cancer reconstruction and cosmetic purposes [371]. Due to the lack of specific tracking of implant textures, the risk of this disease remains inconclusive at both individual and societal levels [372]. Furthermore, the effects of nanostructured surface topography on interactions with surrounding tissues have not been extensively studied from a nanotoxicology perspective, as highlighted in the regulatory discussions in Sect. “Current regulatory policies around the globe”.

Patient-based investigations have confirmed that the surface roughness of silicone mammary implants significantly influences both acute and early-stage chronic fibrotic responses [373]. The adherence of proteins to rough surfaces can mediate pro-inflammatory and profibrotic processes, suggesting that surface roughness plays a role in modulating fibrosis and enhancing the immune response [373]. Additionally, another study demonstrated that nanostructures with a higher aspect ratio on titanium substrates can alter the elastic modulus of the material surface, thereby inducing macrophage polarisation toward the M1 phenotype and triggering intense immune responses [374]. These findings underscore the importance of considering surface topography in the design of nanomaterials, as it plays a crucial role in interactions with proteins and subsequent local biological reactions, beyond the well-studied effects of surface chemistry.

Furthermore, the inflammatory response is amplified by the activation of the complement system, which leads to the production of chemoattractant molecules that recruit and activate additional innate immune cells [375]. Over the years, there has been growing recognition of the role that complement activation plays in the interaction of engineered nanomaterials, including nanomedicines, with the immune system. While it is well-established that surface properties such as chemistry, area, and energy influence protein adsorption, including that of complement proteins, the mechanisms by which the complement system is specifically activated at sites of local exposure to nanomaterials remain less understood. These gaps in understanding highlight the need for further research into how surface properties contribute to immune responses at the molecular level.

##### Carcinogenicity

The assessment of carcinogenicity risk for nanomaterials typically relies on theoretical analysis and existing data rather than new carcinogenicity testing, especially when the risks can be adequately evaluated or managed without additional studies [376]. Long-term carcinogenicity studies are costly and time-consuming, often requiring two to three years to complete, which poses significant economic concerns for manufacturers. Therefore, understanding the genotoxicity of nanomaterials is crucial, as genotoxicity is a key aspect of carcinogenesis. Recent research has extensively studied the genotoxic effects of nanomaterials and the mechanisms leading to transient or permanent genetic changes [377–379]. Genotoxicity is generally categorised into two main mechanisms: primary and secondary.

Primary genotoxicity can be further divided into direct and indirect mechanisms. Direct genotoxicity occurs when nanomaterials physically interact with DNA within the nucleus, leading to DNA damage such as breaks, lesions, or chromosomal alterations. Indirect genotoxicity, on the other hand, is often associated with the generation of ROS induced by nanomaterials or the release of toxic ions from their dissolution. These ROS can cause significant DNA damage through oxidative stress mechanisms, such as the Fenton-type reaction described in Sect. “Oxidative stress and high reactivity”. To determine whether primary genotoxicity is direct or indirect, it is essential to investigate the mechanisms of nanomaterial uptake and their ability to enter the nucleus, as discussed in Sects. “Transcellular transport mechanisms” and “Paracellular transport mechanism”. For instance, smaller nanomaterials measuring only a few nanometres can penetrate the nucleus via nuclear pores. However, larger nanomaterials have also been observed within the nucleus, suggesting alternative pathways for nuclear entry, such as endocytosis [380].

Secondary genotoxicity represents the predominant mechanism of nanomaterial-induced genotoxicity and is mediated by ROS produced by inflammatory cells [357]. When nanomaterials activate phagocytes, they can initiate an oxidative burst, triggering an inflammatory response aimed at clearing foreign materials. If this clearance is unsuccessful, as is often the case with inhaled nanomaterials, it can lead to a chronic immune response and sustained genotoxic effects. This form of genotoxicity is challenging to study using standard in vitro methods and has primarily been investigated in vivo, focusing on chronic inflammation caused by immune cells such as macrophages and neutrophils [377, 381]. These findings underscore the importance of carefully designing the surface chemistry and size of nanomaterials to control their ROS-inducing potential, which is critical during the manufacturing development phase.

Several studies have highlighted the variability in genotoxic effects depending on the type and coating of NPs. For example, Uygur et al*.* compared the toxicity of dialysed pristine Ag NPs with a size of 15 nm, PEG-coated Ag NPs (PEGylated Ag NPs), and silver nanorods across various cell lines [382]. Their findings demonstrated that all these forms of Ag NPs were genotoxic to peripheral blood cells. Similarly, Foldbjerg et al*.* investigated polyvinylpyrrolidone (PVP)-coated Ag NPs, with primary sizes exceeding 50 nm, using human adenocarcinomic alveolar epithelial cells (A549) [383]. They detected bulky DNA adducts in the nucleus through ^32^P post-labelling, which strongly correlated with oxidative stress. Conversely, Singh et al*.* utilised sophorolipid, a novel glycolipid, to conjugate 10 nm Ag NPs and found no genotoxicity in human liver carcinoma cells (Hep G2) at concentrations up to 100 μg/mL [384]. Additionally, Kim et al*.* reported that Ag NPs caused DNA damage in BEAS-2B cells and mouse lymphoma cells (L5178Y), although they did not exhibit mutagenicity [385]. Recent studies have also found that starch-coated Ag NPs, with primary sizes ranging from 6 to 20 nm, were cytotoxic, genotoxic, and antiproliferative in human fibroblasts and glioblastoma cells [386]. These findings suggest that the genotoxicity of Ag NPs is highly dependent on their size and surface coating, highlighting the importance of surface properties in determining their biological interactions and effects.

The role of surface properties is also evident in semiconductors like metal oxides, which generate electron–hole pairs upon energy exposure (*e.g.*, from light) a and subsequently produce ROS. The risk of oxidative stress from these materials is significantly influenced by the chemical interactions of their surfaces with the environment. Rossi et al*.* demonstrated that the pulmonary effects observed in mice following acute and subacute inhalation of various coated or uncoated TiO_2_ NPs could not be attributed to particle size or surface area alone [387]. Instead, these effects were primarily influenced by the surface coating of the TiO_2_ particles. Among four types of TiO_2_ NPs with diameters ranging from 10 to 40 nm, only the uncoated TiO_2_ NPs did not induce pulmonary inflammation, likely due to differences in electron–hole recombination tendencies caused by the coatings. This electron–hole recombination effect is a well-known mechanism in the photocatalytic field [388, 389].

In summary, the genotoxicity and carcinogenicity of nanomaterials are complex phenomena influenced by multiple factors, including size, surface chemistry, and coating materials. A comprehensive understanding of these factors is essential for accurately assessing the risks associated with nanomaterials and for guiding their safe design and application in various fields.

##### Special considerations

In risk analysis, special considerations must be tailored to specific product scenarios. For example, for products that involve blood contact, beyond conventional toxicity risks, it is crucial to consider haemocompatibility. Given the broad scope of this comprehensive review, which encompasses a wide range of product types, it is challenging to systematically analyse each specific hazard in detail. Therefore, we have chosen to highlight some recent studies on the risks associated with nanomaterials. Keeping abreast of the latest research findings is vital for risk analysis and is particularly important for R&D engineers and scientists in developing safe and effective nanomaterials.

Jack et al*.* recently conducted a study on the toxicity potential of highly purified, metal-free, and endotoxin-free GO nanosheets [390]. The study focused on two precisely defined size ranges of GO nanosheets: small (ranging from 2 μm to 50 nm) and ultrasmall (ranging from 300 to 10 nm). The findings revealed that when introduced into the human airway, these GO nanosheets formed airborne aggregates with a median size of 80–90 nm. This size distribution is significant for blood-contact products, as the study also found that both small and ultrasmall GO increased blood thrombogenicity in in vitro coagulation assays compared to air exposure. These results suggest that the thrombotic potential of GO must be carefully considered in the design of products involving blood contact.

Similarly, a study on nano-silicon dioxide (nano-SiO_2)_ investigated the cytotoxic effects of orally administered nano-SiO_2_ particles ranging in size from 20 to 30 nm at doses of 300, 600, and 900 mg/kg/day over 20 days [391]. The results indicated varying levels of cytotoxicity, with the highest doses causing significant toxicity in the liver and lungs, and the intermediate dose primarily affecting the kidneys and lungs. These findings provide essential insights into the risk assessment for oral nanomedicine applications, highlighting the need for careful dose and toxicity evaluations based on specific organ sensitivity.

These studies not only deepen our understanding of nanotoxicity but also underscore the complexity of assessing risks associated with nanomaterials. They highlight the importance of not assuming all risks are fully controlled and the need for thorough biological risk evaluations. For scientists and R&D engineers, regularly reviewing and analysing the latest research developments is crucial for guiding the development of safe and effective nanomaterials. By integrating the most current scientific insights into their design and evaluation processes, researchers and developers can better anticipate and mitigate potential risks associated with nanomaterial use.

### Environmental risks

Nanotechnology has the potential to be a beneficial tool in environmental cleanup efforts by aiding in the treatment and prevention of pollution from hazardous substances [392–394], but it can also present risks to the ecosystem if not properly managed. One major concern is that the unique properties of nanomaterials, such as their small size and high reactivity, can lead to unexpected ecological consequences. Studies have shown that nanomaterials, such as Ag NPs and TiO_2_ NPs, can induce toxicity in aquatic and terrestrial organisms, which may disrupt local ecosystems and biodiversity [395, 396].

In the evaluation of risks from newly created engineered nanomaterials, it is widely agreed, and we share this view, that while the conventional approach to environmental risk assessment, which involves assessing both exposure and potential hazards, remains applicable to nanotechnology, it must be further adapted to consider the specific mechanisms unique to nanomaterials. Handy et al*.* have offered an extensive review based on regulatory guidelines, including a flowchart for assessing toxicity in water and soil environments and a summary of standard tests of the Organisation for Economic Co-operation and Development (OECD) for ecotoxicity [397].

Additionally, there have been calls for caution regarding environmental considerations, suggesting that hazard assessments for nanomaterials should also encompass studies on their behavior in environmentally aged states, including non-nanomaterial entities like dissolution products. Once nanomaterials are released into natural ecosystems, they may undergo transformations that significantly affect their toxicity and environmental fate. Thus, more comprehensive research is needed on how environmental conditions, such as pH, temperature, and the presence of natural organic matter, affect the surface chemistry, aggregation state, and solubility of nanomaterials. These factors, in turn, influence their interaction with biota and ecological systems.

Generally, risk assessment for nanomaterials should cover five stages across the lifecycle: resource acquisition, production process, utilisation stage, elimination process, and waste management. Furthermore, the risk assessment must ensure the integrity and standardisation of the evaluation across these stages, with special emphasis on the waste management stage. A critical challenge in this assessment is the development of reliable environmental monitoring techniques that can track the concentration, transformation, and bioavailability of nanomaterials in different environmental compartments (*e.g.,* air, water, soil). A more integrated approach is suggested, combining multiple techniques such as single-particle inductively coupled plasma mass spectrometry (spICP-MS), transmission electron microscopy (TEM), and field-flow fractionation (FFF) coupled with light scattering detection to achieve real-time and precise detection of nanomaterials in complex matrices [398, 399].

Upon entering natural environments, nanomaterials may degrade into various forms under the influence of environmental processes, resulting in the final environmental aged state, which includes changes like surface transformation and dissolution [400]. For instance, the aging process of nanomaterials in aquatic environments can result in the formation of new nanomaterial species with altered reactivity and toxicity. Therefore, risk assessments should not only focus on pristine nanomaterials but should also extend to the aged forms, as these might pose different risks. Long-term monitoring and predictive modeling of these aged forms are crucial to assess their cumulative ecological impacts.

A research work about the lifecycle aging of Ag and TiO_2_ nanomaterials and its distribution of the different environmental aged states was done by Adam et al*.* [401]. It was modeled that release into surface water and soil primarily occur in altered forms (with 34–58% and 78–86% in the 25th and 75th percentiles, respectively, averaging 53% and 82%), whereas air releases were mainly in unaltered or matrix-encapsulated forms (ranging 38–46% and 36–44%, with averages of 42% and 40%). The variation in the release pathways across different environmental compartments highlights the need for a more systematic study of the nanomaterial release patterns in each medium and their potential environmental and ecological impact [401–403].

In response to the complex transformation pathways of nanomaterials, such as hetero-aggregation, dissolution, and sulphidation, it is recommended that risk assessments incorporate detailed monitoring and predictive models of these processes within natural ecosystems [404, 405]. Monitoring should focus on both short-term and long-term effects of nanomaterial exposure in critical environmental compartments. Furthermore, the integration of environmental data from different regions and ecosystems is essential to better understand the global distribution and impact of nanomaterials, which can support more informed regulatory frameworks.

Once nanomaterials enter ecosystems through industrial discharges or agricultural activities, they may affect the growth and development of rare medicinal plants, crop yield, and the metabolism of primary and secondary metabolites [406]. TiO_2_ NPs, commonly used in daily life, were studied for their effect on Luteolin, a sensitive anticancer flavonoid [407]. The findings showed that the impact was directly proportional to the level of nanoparticle contamination. Flavonoids can undergo various chemical modifications when exposed to metallic oxide NPs, depending on factors such as pH, temperature, and the presence of other chemical species.

In conclusion, the environmental risk analysis approach should be improved to fully account for the environmental aging of nanomaterials through specific transformation pathways such as hetero aggregation [408], dissolution [409], and sulphidation [404]. These transformations largely depend on the characteristics of the nanomaterial like size and surface coating, as well as the nanomaterial’s interact with various environmental components, such as water, soil, and living organisms which also determined largely on the surface chemistry of the nanomaterial. For a scientifically sound risk assessment, tailored methods should be developed by the manufacturers or other responsible entities based on specific application scenarios.

### Safety risk analysis consideration

Based on the mechanisms outlined above, we recommend the following guidelines for medical product manufacturers conducting risk analysis of nanotechnology-derived medical products:

- pay higher attention to NPs due to their higher mobility;
- carefully consider the surface properties of all materials. For example, a nano-structured coating might not release NPs but could still pose cytotoxicity through physical interaction mechanisms when in direct contact;
- when evaluating the properties of a nanotechnology-derived medical product, especially the nanoscale materials it might contain or produce throughout its lifecycle (such as size, size distribution, surface modification, redox potential, etc.), remember that no universal rule has been established. Therefore, a characterisation protocol based on scientific rationale is necessary for both documentation and result reliability;
- for designing a reasonable characterisation protocol, refer to relevant technical manuals [410–412]. However, these manuals are just a starting point aimed at distinguishing “normal bulk materials” from “nanomaterials” to facilitate future evaluation. Medical product manufacturers should thoroughly scrutinise their product;.
- regardless of the characterisation results, they only serve as input data for further toxicity or safety evaluation rather than as rigid indicators. For example, a material with nanoscale dimensions does not unconditionally show higher toxicity than its bulk form;
- the nano-safety risks associated with nanoscale materials or other forms of products involving nanotechnology can be analysed or controlled using the mechanisms outlined above.

## Current regulatory policies around the globe

Previous reviews have highlighted that key regional and national regulatory agencies have been proactively engaging in the oversight of nano-enabled products and collaborating to form a global consensus on standardisation and regulation [413]. Initiatives aimed at the evaluation and regulation of nanomaterials have been launched by Europe, the United States, and China.

In 2003, the United States officially proposed examining the possible impacts of synthetic nanomaterials on human health and the environment. By 2004, the Environmental Protection Agency (EPA) in the United States had established the direction for environmental and health safety research concerning synthetic nanomaterials, focusing on their environmental distribution, movement, and transformation, as well as their toxicological attributes. In 2007, the EPA in the United States released the Nanotechnology White Paper, which comprehensively addressed the risks to ecological toxicity and human health presented by synthetic nanomaterials. Following this, in 2009, the EPA issued the “Nanomaterials Research Strategy,” aimed at guiding scientists and regulatory bodies in investigating the possible environmental and health hazards associated with synthetic nanomaterials. In 2011, the President's Office along with the National Science and Technology Council released a White paper titled “Nanotechnology Environment, Health, and Safety Research Strategy,” incorporating nanotoxicology into the national research agenda. In the same year, the FDA published preliminary guidance to assist companies in determining the applicability of nanomaterials to their products.

The European Union has also conducted research on the environmental and biosafety assessments of synthetic nanomaterials. In 2005, the Scientific Committee on Emerging and Newly Identified Health Risks (SCENIHR) of the European Commission released “Artificial Products and by-products of Nanotechnology,” providing a systematic review of the toxicity, environmental impacts, and potential health dangers posed by nanomaterials.

In 2007, discussions commenced in Europe regarding the adaptation of the existing REACH regulation (Registration, Evaluation, Authorisation, and Restriction of Chemicals) for application to nanomaterials. Moreover, the updated European cosmetic regulations mandated that starting from 2013, any products containing synthetic nanomaterials must clearly label them as such.

Overall, for regulatory purposes, an initial clear definition was necessary. Factors to be considered in defining *nano* include: (i) size, (ii) size distribution, (iii) specific surface area, (iv) surface modification, (v) other physicochemical properties, (vi) whether the nanomaterials are organic or inorganic, (vii) nanocomposites, (viii) durability, (ix) whether the nanomaterials are manufactured or natural.

Size is considered the primary criterion when defining nanomaterials. The European Commission Recommendation from 10th June 2022, regarding nanomaterial definitions, reinforces this by adhering to the 2010 SCENIHR opinion, defining 'nanoscale' as ranging from 1 to 100 nm. Similarly, the FDA's 2014 Guidance for Industry on nanotechnology applications specifies that for a product to be considered under nanotechnology, it should be engineered with at least one dimension, or have internal or surface structures, within the nanoscale range of approximately 1 nm to 100 nm. Additionally, the NMPA's 2021 Guiding Principles for medical devices using nanomaterials describe nanomaterials as having any external dimension or internal/surface structure in the nanoscale, specifically between 1 and 100 nm, a range which often, but not always, presents unique properties not seen in larger scales. Despite the global consensus on the 100 nm threshold, it is emphasised that this measurement is a guideline rather than a strict indicator of toxicity or safety. Materials smaller than 100 nm are not inherently toxic, nor are those larger necessarily safe. As stated in the “Scientific Basis for the Definition of the Term ‘Nanomaterial’”, the term ‘nanomaterial’ simply refers to a material categorised by the size of its parts and does not inherently indicate a specific risk or hazard. The designation ‘nano’ does not automatically mean the material possesses novel properties compared to its larger or smaller counterparts. However, the size of a material does affect its biodistribution and distribution kinetics within organisms and ecosystems, a statement that is widely agreed upon in the scientific community.

This leads to a secondary criterion for defining nanomaterials, the “change of physicochemical properties”. Both the FDA and NMPA guidelines address whether a material or finished product is designed to demonstrate properties or effects, including physical, chemical, or biological, attributable to its dimensions, even if these dimensions are beyond the nanoscale range, extending up to one micrometer (1000 nm).

However, it seems the EU regulatory body has not set a similar standard, which we believe is due to an intentional emphasis on NPs rather than nanostructures, such as coatings, as indicated in clause 11: “The definition should not apply to large, solid products or components, even when they possess an internal or surface structure at the nanoscale, such as coatings, some ceramic materials, and complex nanocomponents, including nanoporous and nanocomposite materials.” This implies that while there is a consensus on the nano dimension being defined as 1 to 100 nm, regulations across different regions are not completely aligned regarding whether zero-dimensional (e.g., QDs), one-dimensional (e.g., nanotubes, nanofibers), or two-dimensional (e.g., surface coatings with nano thickness or dispersed nanosheets) materials should be uniformly regulated under the same criteria or framework.

Currently, among the various parameters considered for defining nanomaterials, the size requirement has been commonly addressed in many regions, whilst the EU has uniquely mentioned the specific surface area as a reference, and the FDA and NMPA have highlighted unique physicochemical properties. Another aspect that requires clarification is whether the materials are “manufactured or incidentally produced”. The EU’s Clause 6 expressly states that the nanomaterial definition should encompass natural, incidental, or manufactured materials, mirroring the stance in China where NMPA includes incidental nanomaterials under its regulatory framework. Conversely, the FDA’s guidelines specifically employ the term “engineered” to differentiate between deliberately modified nanotechnology products and those that might inadvertently contain nanoscale materials, pointing out that incidental NPs in conventionally manufactured products fall outside the scope of the guidance. It is also noteworthy that ISO 80004 series standards are dedicated to engineered nanotechnologies, including coatings among others.

**Fig. 34:**
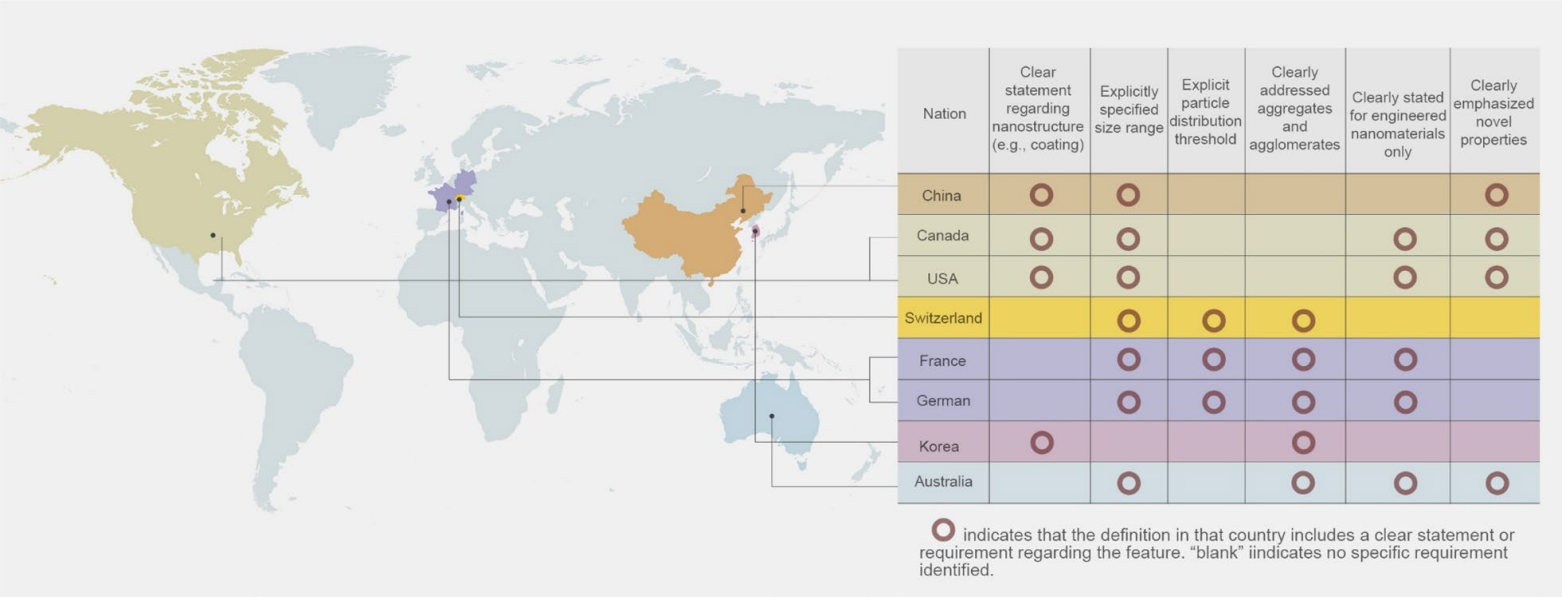
Schematic representation of the overall assessment of environmental safety risks induced by nanomaterials, and the variety in the final state and distribution of nanomaterials cause by environmental processes, leading to the complexity of ecotoxicity caused by NPs

As of now, there are noticeable differences and ambiguities in the regulatory boundaries of nanotechnology, which could lead to confusion among companies without a solid scientific background, relying solely on regulatory texts. Nevertheless, a definitive compilation of terms would still be beneficial. The Joint Research Centre of the European Commission provides a summary of the core elements found in nanomaterial definitions across global organisations, and an analysis illustrating the similarities and distinctions in the regulatory definitions of nanomaterials across Europe, the United States, and China, and other major nations is presented in Fig. 34.

Although regulatory stipulations vary from one nation to another, this does not imply that potential risks, such as nano-structured coatings in the EU or accidentally produced NPs in the US, should be disregarded; instead, these should be addressed through a framework designed for risk analysis papers. In fact, a case-by-case methodology for the safety evaluation and risk assessment of nanomaterials has been advocated by various authorities (EFSA in 2009, FDA in 2007, SCENIHR in 2009), since deducing risks from one nanomaterial to another is not deemed practical, even if they share the same basic chemical composition. The provision of our interpretation of both the scientific basis and the regulations is aimed at assisting companies and professionals in acquiring a more defined direction for this type of risk assessment.

In the field of medicine and medical device registration, the adoption of a harmonised biological risk evaluation framework based on ISO 10993, which prioritises a risk-analysis approach over rigid testing protocols, indicates that the absence of precise definitions is unlikely to impede the innovative use of nanotechnology in the development of global healthcare products. For the safety assessment of specific nanomaterials, the European Parliament's REACH framework provides a versatile method for hazard evaluation and risk characterisation, making it particularly suitable for nanomaterials. REACH also promotes the use of computational methods, such as Quantitative Structure–Activity Relationships (QSARs), which has facilitated significant advancements in risk assessment despite the scarcity of experimental data. Moreover, the regulatory landscape is progressively aligning with the view that vertebrate animal testing should be employed only as a last resort, advocating for alternative testing approaches. However, to further enhance regulatory frameworks, there is a critical need to develop standardised nanomaterial characterisation data, employing harmonised testing standards and ensuring consistent reporting of endpoints. Currently, the availability of test data from the industrial sector is constrained by financial limitations and technological competition.

To improve the regulatory framework for nanomaterials globally, it is crucial to implement several key strategies that enhance clarity, consistency, and scientific rigor. First, establishing a unified global database for nanomaterials would facilitate the consolidation of definitions, classifications, uses, and risk assessments from different regions. Such a database would provide a centralised resource that fosters information sharing and regulatory harmonisation, enabling companies to better navigate and comply with various regional regulations. Second, developing standardised tools and methods for the risk assessment of nanomaterials is essential. Standardisation would not only streamline the evaluation processes across different regulatory bodies but also ensure that risk assessments are comparable and consistent globally. This would help mitigate discrepancies in regulatory practices and provide a more coherent approach to understanding and managing the potential risks associated with nanomaterials. Third, introducing a dynamic regulatory mechanism that evolves in response to technological advancements and emerging scientific research is vital. Regulations should be adaptable to accommodate new findings and innovations in nanotechnology, ensuring that they remain relevant and scientifically sound. This approach would prevent regulations from becoming outdated and inadequate, thus maintaining a high standard of safety and efficacy. Fourth, promoting interdisciplinary research collaboration is necessary for a comprehensive understanding of nanomaterials' behaviour and their potential environmental and health impacts. By integrating insights from fields such as chemistry, materials science, biology, and toxicology, regulatory strategies can be more effectively tailored to address the complex nature of nanomaterials. Finally, developing clear guidance documents and technical standards would address current ambiguities within existing regulations. This includes providing explicit definitions and regulatory requirements for incidentally produced nanomaterials, which would reduce confusion among companies and ensure a more straightforward interpretation of regulations.

Implementing these improvements would enhance the efficiency and effectiveness of nanomaterial regulation, fostering safer and more sustainable development of nanotechnology on a global scale.

## Conclusions and outlook

Nanotechnology is based on a variety of materials including organic, inorganic, carbon-based, and composite. As dimensions are reduced to the nanometer scale, materials scientists provide a range of design options such as discrete particles, unique shapes, or nanostructured coatings, among others. These designs showcase distinctive benefits as innovative nanomedicines, functioning as drug carriers or therapeutic agents, or when incorporated to improve the performance of NMDs, such as enhancing detection capabilities, antibacterial properties, and so forth. Yet, when entering the market, a comprehensive examination of product lifecycle risks associated with nanotoxicity reveals numerous safety concerns, and the potential mechanisms were explored here.

The safety risks associated with nanotechnology in healthcare are multifaceted, often influenced by factors such as surface reactivity, size, and shape of nanomaterials. These same attributes, crucial for the efficacy of nanomedicine, also pose potential safety hazards. Understanding and addressing these risks require a focus on surface characteristics and their deviations from the original state.

Environmental considerations are paramount throughout the lifecycle of nanotechnology products. While traditional hazard evaluation methods can be adapted for nano-specific aspects, the aging process of nanomaterials significantly impacts their environmental fate, emphasising the need for comprehensive assessments.

Moreover, the complex nature of nanomaterials necessitates tailored risk assessments that account for individual material properties and toxicological profiles. A nuanced approach, aligned with regulatory standards, would enhance risk management strategies and ensure the responsible use of nanotechnology in healthcare.

The evolution of nanotechnology in healthcare underscores the critical need for close collaboration among researchers, industry professionals, regulatory bodies, and healthcare practitioners. Successfully translating research advancements into market-ready applications requires prioritizing comprehensive risk assessment strategies, particularly with regard to long-term toxicity, which remains both the costliest and most critical aspect. Given the complexity of this research area, advancing the safe and effective integration of nanotechnology into healthcare hinges on robust interdisciplinary collaboration. By working together, we can harness the transformative potential of nanotechnology while safeguarding human health and environmental sustainability. This will involve the development of a more comprehensive regulatory framework, informed by cutting-edge scientific insights and supported by transparent communication across all sectors. Through these efforts, we can ensure the safe, effective, and sustainable deployment of nanotechnology in healthcare.

## Data Availability

No datasets were generated or analysed during the current study.

## Abbreviations

510(k): 510(k) of the food, drug and cosmetic act
ADA: Adenosine deaminase
Ag: Silver
AJ: Adherent junction
Al: Aluminum
ALD: Atomic layer deposition
ATP: Adenosine triphosphate
Au: Gold
BET: Brunauer–Emmett–Teller
BIA-ALCL: Breast implant-associated anaplastic large cell lymphoma
Bi: Bismuth
BMVEC: Pristine graphene on brain microvascular endothelial cell
BP: Black phosphorus
C_60_: Fullerenes
CAGR: Compound annual growth rate
Cd: Cadmium
CDs: Carbon dots
CdTe: Cadmium telluride
CeO_2_: Cerium oxide
CNA35: Collagen-binding adhesion protein 35
CFs: Carbon nanofibers
CKD: Chronic kidney disease
CNTs: Carbon nanotubes
CoFe_2_O_4_: Cobalt ferrite
CT: Computerised tomography
Cu: Copper
CuO: Copper oxide
CVD: Chemical vapor deposition
DOX: Doxorubicin
EFSA: European Food Safety Authority
EMA: European Medicines Agency
EPA: Environmental Protection Agency
EPO: Erythropoietin
EPR: Permeability and retention
EPS: Extracellular polymeric substance
EU: European Union
EUDAMED: European Database on Medical Devices
FDA: The United States Food and Drug Administration
Fe: Iron
Fe_3_O_4_: Iron oxide
FFF: Field-flow fractionation
FMDV: Foot-and-mouth disease virus
GBM: Glioblastoma multiforme
Gd: Gadolinium
Gel: Gelatin
Gd: Gadolinium
GH: Growth hormone
GJ: Gap junction
Gly: Glycyrrhizic acid
GO: Graphene oxide
GQDs: Graphene quantum dots
Gr: Graphene
HA: Hydroxyapatite
HIV-1: Human immunodeficiency virus
ICD: Immunogenic cell death
INF: Alpha interferon
ISC: Intersystem crossing
KS: Kaposi’s sarcoma
La_2_O_3_: Lanthanum oxide
L-cysteine: Chiral amino acid
LipSiNs: Lithiated porous silicon nanowires
MgAl_2_O_4_: Magnesium aluminum oxide
MgO: Magnesium oxide
MIP NPs: Molecularly imprinted polymeric nanoparticles
Mn: Manganese
MOF: Metal–organic frameworks
MPS: Mononuclear phagocytosis system
MRI: Magnetic resonance imaging
MS: Multiple sclerosis
MSNs: Mesoporous silica nanoparticles
NGPE: N-glutaryl phosphatidylethanolamine
NIH: The United States National Institutes of Health
NLM: National Library of Medicine
NMDs: Nanotechnology-based medical devices
NMPA: National Medical Products Administration
NO: Nitric oxide
NPs: Nanoparticles
NSCLC: Non-small-cell lung cancer
OECD: The Organisation for Economic Co-operation and Development
Pb: Lead
PDT: Photodynamic therapy
PET: Positron emission tomography
PETE: Polyethylene terephthalate
PGA: Poly (glycolic acid)
PIII: Plasma-immersion ion implantation
PIA: Polysaccharide intercellular adhesin
PLA: Poly (lactic acid)
PLGA: Poly (lactic-co-glycolide acid)
PMA: Premarket approval
PMONPs: Periodic mesoporous silica nanoparticles
PNIPAAm: Poly (*N*-isopropylacrylamide)
PRRSV: Porcine reproductive and respiratory syndrome virus
PS: Photosensitiser
pSiNPs: Porous silicon nanoparticles
PTT: Photothermal therapy
PU: Polyurethane
PVA: Polyvinyl alcohol
PVP: Polyvinylpyrrolidone
QS: Quorum sensing
QSARs: Quantitative structure–activity relationships
R&D: Research and development
REACH: Registration evaluation, authorisation, and restriction of chemicals
RNS: Reactive nitrogen species
ROS: Reactive oxygen species
RRMS: Relapsing remitting multiple sclerosis
SBA2: Silica-based glass particles
SCENIHR: Scientific committee on emerging and newly identified health risks
SERS: Surface-enhanced Raman spectroscopy
spICP-MS: Single-particle inductively coupled plasma mass spectrometry
SF: Silk fibroin
SPARC: Secreted protein acidic and rich in cysteine
SPIONs: Superparamagnetic iron oxide nanoparticle
Sr: Strontium
TCP: Tricalcium phosphate
TEM: Transmission electron microscopy
TiO_2_: Titanium dioxide
TJ: Tight junction
TPE: Tetraphenylethene
TTR: Transthyretin
V_2_O_5_: Vanadium pentoxide
VE: Vascular endothelial
W: Tungsten
Zn: Zinc
ZnO: Zinc oxide
ZrO_2_: Zirconium dioxide
